# A rational pre-catalyst design for bis-phosphine mono-oxide palladium catalyzed reactions†
†Electronic supplementary information (ESI) available: Detailed experimental procedures and characterization data for all new compounds. CCDC 1512973–1512975. For ESI and crystallographic data in CIF or other electronic format see DOI: 10.1039/c6sc05472b



**DOI:** 10.1039/c6sc05472b

**Published:** 2017-01-19

**Authors:** Yining Ji, Hongming Li, Alan M. Hyde, Qinghao Chen, Kevin M. Belyk, Katrina W. Lexa, Jingjun Yin, Edward C. Sherer, R. Thomas Williamson, Andrew Brunskill, Sumei Ren, Louis-Charles Campeau, Ian W. Davies, Rebecca T. Ruck

**Affiliations:** a Department of Process Research & Development , Merck & Co., Inc. , Rahway , New Jersey 07065 , USA . Email: yining.jichen@merck.com

## Abstract

Significant catalyst loading reduction and increased reaction robustness have been achieved for a Pd-catalyzed asymmetric intramolecular C–N coupling through comprehensive mechanistic studies. Detailed kinetic, spectroscopic, and crystallographic analyses revealed that the mono-oxidation of the bis-phosphine ligand is critical for a successful transformation. ^31^P NMR studies provided an understanding of the inefficient activation of the Pd(OAc)_2_/(*R*,*R*)-QuinoxP* pre-catalyst to form the active bis-phosphine mono-oxide–Pd(0) catalyst with competitive formation of a less active (*R*,*R*)-QuinoxP*·PdBr_2_ complex. Based on these detailed mechanistic studies, a new series of bis-phosphine mono-oxides (BPMO)-ligated Pd(ii) pre-catalysts have been rationally developed that allow for reliable and complete catalyst activation which should have general utility in academic and industrial settings.

## Abstract 2

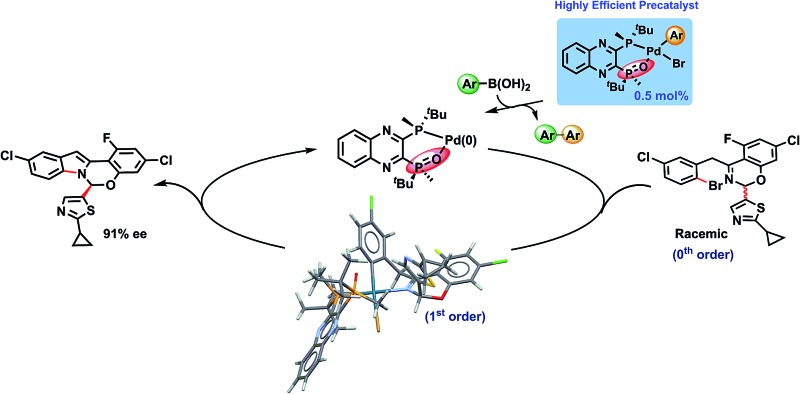
Detailed mechanistic studies of a Pd-catalyzed asymmetric C–N coupling led to a rational design of a new series of bis-phosphine mono-oxides ligated Pd(ii) pre-catalysts that allow for reliable and complete catalyst activation.

## Introduction

Since Noyori's introduction of BINAP for the Ru-catalyzed asymmetric hydrogenation of ketones,^1^ chiral bis-phosphines have arguably emerged as the most ubiquitous privileged ligand structure in asymmetric catalysis.^2^ In contrast, chiral bis-phosphine mono-oxides (BPMO) have found far less frequent application, presumably due to their limited availability and a dearth of information on their reactivity.^3^ The unique combination of a stronger (P) and weaker (P=O) donor within the same ligand provides the flexibility to act as either bi- or monodentate when bound to transition metals. The hemi-labile character of the BPMO ligands plays an important role in catalysis, since it can provide an open coordination site on the metal center as needed. In fact, different reactivities have been observed with the utilization of BPMO ligands compared to their cognate bidentate bis-phosphine ligands.^4^ Significantly, in several examples, the bis-phosphine mono-oxide provides the opposite^5^ or enhanced^6^ enantioselection in asymmetric synthesis. In other cases, the BPMO ligands have also proved to be catalytically superior compared to the parent bis-phosphine.^7,8^ In these instances, the metal bis-phosphine ligand combinations have proven ineffective as pre-catalysts, since the ligand needs to undergo phosphine mono-oxidation to generate the active metal–BPMO catalyst. Therefore, the requirement for oxidation has profound implications for catalysis and new asymmetric reaction design in particular, where both hypothesis-driven design and serendipity are always in play. A robust pre-catalyst platform for easy conversion of bis-phosphines to bis-phosphine mono-oxides in catalytic reactions would enable facile and definitive study of these promising ligands in novel asymmetric transformations.

**Scheme 1:**
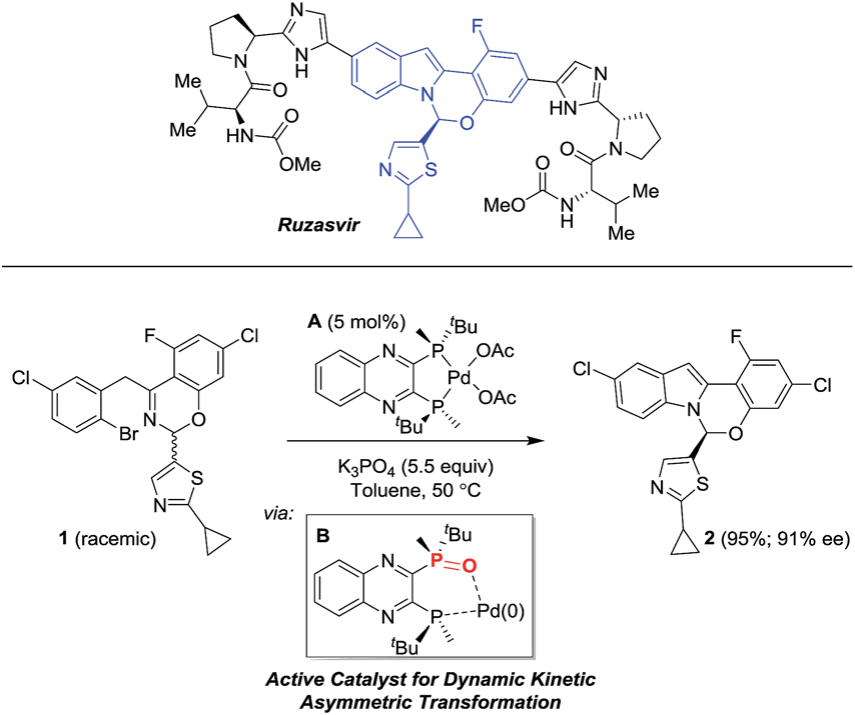
Pd-catalyzed asymmetric synthesis of the benzoxazino-indole core of ruzasvir.

We recently reported^9^ a novel, enantioselective hemiaminal synthesis *via* a palladium-catalyzed C–N coupling using chiral bis-phosphine ligands (Scheme 1) that has been used in the preparation of ruzasvir, a clinically relevant hepatitis C virus NS5A inhibitor.^10^

**Fig. 1:**
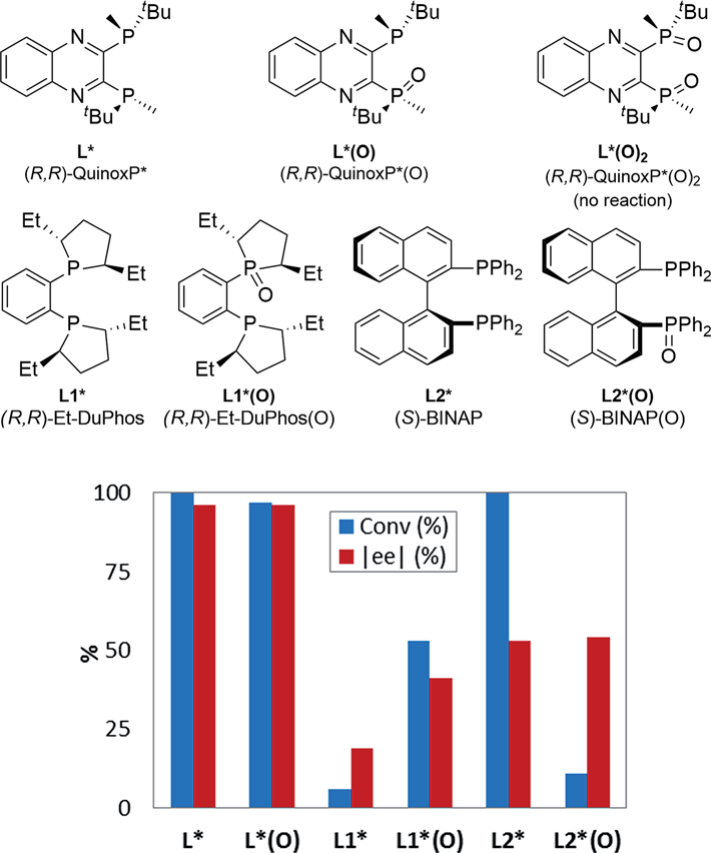
Comparison of reaction conversion and enantioselectivity of different ligands with respect to their parent mono-oxidized form for the reaction in Scheme 1. Note: in screening mode, the pre-catalyst is formed *in situ* from a mixture of Pd(OAc)_2_ and the corresponding bis-phosphine ligand.

In this Pd-catalyzed process, we also observed that the *in situ* mono-oxidation of the bis-phosphine ligand (*R*,*R*)-QuinoxP* (abbreviated as L*) to access the active catalyst L*(O)Pd(0) **B** from the pre-catalyst L*Pd(OAc)_2_ **A** was critical for effective catalysis. However, different ligands displayed diverse behaviors as the bis-phosphine *versus* their mono-oxide (Fig. 1). While (*R*,*R*)-QuinoxP* and the parent mono-oxide gave similar reaction conversions under screening conditions, (*R*,*R*)-Et-DuPhos proved to be inferior to its respective mono-oxide derivative and finally, fully reversed reactivity was observed for (*S*)-BINAP/(*S*)-BINAP(O) system. The inefficient *in situ* oxidation of (*R*,*R*)-Et-DuPhos to form its mono-oxide counterpart and a possible over-oxidation of BINAP(O) leading to the inactive bis-oxide^5,8^ during the reduction of palladium(ii) likely account for the differential reaction performance.^11^

Experimental nuances, such as “age” time^3*e*^ during the pre-catalyst preparation, order of addition of the reagents, ligand/metal ratio^7a,8b^ and the reaction conditions, in general, could all affect the efficiency of the *in situ* oxidation and significantly impact both reaction performance and outcome. Therefore, interpreting the actual intrinsic reactivity of these ligands based solely on the screening results is potentially misleading. In theory, a detailed investigation of the efficiency of pre-catalyst activation and mechanism would be required prior to evaluation of any results, which would undermine the inherent value of high throughput experimentation (HTE).

In this article, we report a systematic kinetic and spectroscopic investigation into the asymmetric C–N coupling reaction shown in Scheme 1. We gained insight into the efficiency of pre-catalyst activation and potential deactivated catalyst states. This deeper understanding enabled the successful development of more effective palladium–BPMO pre-catalysts and an optimized reaction with significantly reduced catalyst loading and increased reaction robustness. Based on these studies, we anticipate that use of these pre-catalysts will also enable a reliable assessment of the innate reactivity of BPMOs and facilitate widespread employment of this under-utilized class of ligands.

## Results and discussion

### Initial kinetic analysis

**Fig. 2:**
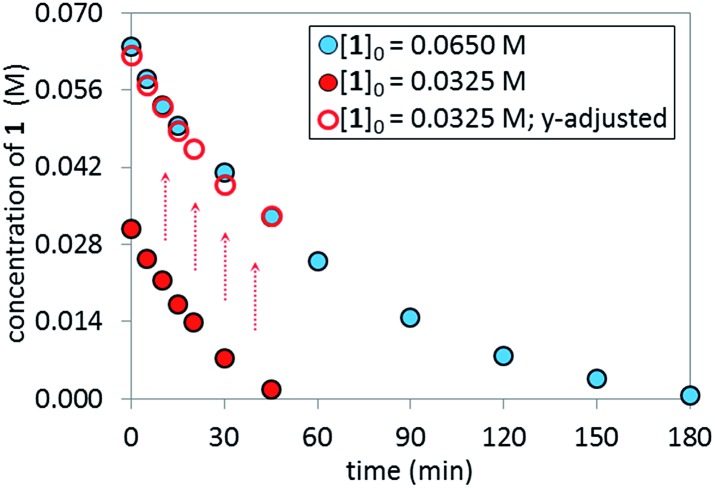
Temporal concentration profiles monitored by ^1^H NMR spectroscopy for the reaction of Scheme 1. [**1**]_0_ as noted; [**A**]_0_ = 3.25 mM; K_3_PO_4_ (5.5 equiv. based on [**1**]_0_ = 0.0650 M and 11.0 equiv. based on [**1**]_0_ = 0.0325 M).

Reaction Progress Kinetic Analysis (RPKA)^12^ of temporal concentration profiles allows kinetic dependences to be determined with a minimal number of carefully designed experiments. Aliquots were withdrawn at regular time intervals and analyzed by ^1^H NMR spectroscopy to track the rate of disappearance of ArBr **1**. Comparison of the slopes of reaction profiles with [**1**] plotted as a function of time with different values of [**1**]_0_ allows assessment of the concentration dependence of ArBr **1**. The slope of the two reactions under different [**1**]_0_ appeared similar, confirmed by comparing these reaction profiles using an “adjusted” profile, as shown in Fig. 2. A simple graphical manipulation moves the reaction with half concentration (red circles) data vertically upward, as indicated by the red arrows. Although these two reactions exhibit different concentrations of **1** at any given point in time, comparable slopes on plots of [**1**] *vs.* time confirmed equal reaction rates. Thus, the data in Fig. 2 enable the conclusion that the reaction must be zeroth-order in substrate **1**.

The current reaction system (Scheme 1) is heterogeneous due to the low solubility of K_3_PO_4_ in toluene. While we see rate acceleration with increasing amount of phosphate (see ESI†), the fact that the reaction exhibited positive first order kinetics in concentration of L*·Pd(OAc)_2_ precursor **A** (see ESI†) indicates that we are not operating under mass transfer limited conditions. The base appears to play two main roles in the system: (1) in combination with water, it facilitates reduction of Pd(ii) to Pd(0) with concomitant phosphine oxidation^13^ and (2) it serves to deprotonate substrate **1** at the benzylic position allowing turnover of the catalytic cycle. The observed rate increase with more base present could be rationalized by either higher surface area and/or increased water content.^14^

### Catalyst robustness

**Fig. 3:**
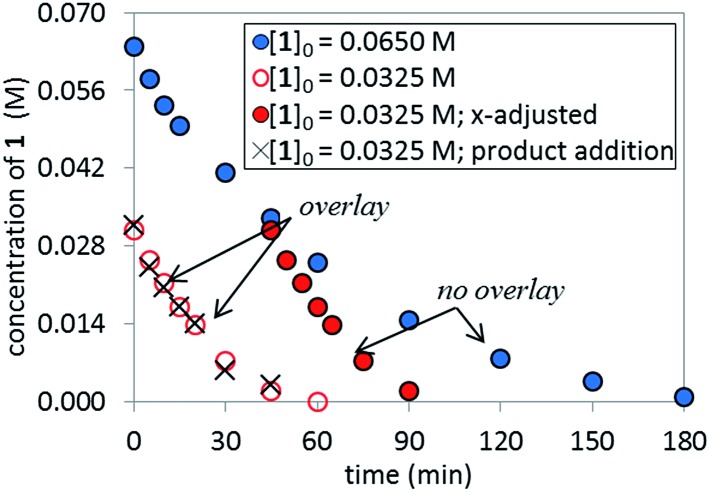
Temporal concentration profiles monitored by ^1^H NMR spectroscopy for the reaction of Scheme 1. [**1**]_0_ as noted; [**A**]_0_ = 3.25 mM; K_3_PO_4_ (5.5 equiv. based on [**1**]_0_ = 0.0650 M and 11.0 equiv. based on [**1**]_0_ = 0.0325 M). Product addition conditions: [**1**]_0_ = 0.0325 M, [**2**]_0_ = 0.030 M.

The temporal concentration profiles in Fig. 2 also provide useful information about catalyst stability. The concentration of ArBr **1** at the beginning of the reaction in red circles was adjusted to be identical to those of a standard reaction (blue circles) after *ca.* 45 minutes (Fig. 3). The lack of overlay in the time-adjusted profiles suggests either catalyst deactivation or product inhibition is occurring. However, by adding the appropriate quantity of product **2** (0.030 M) to mimic the time-adjusted point, excellent superposition was observed, suggesting that it does not interfere with catalyst turnover. Additionally, the rate of the reaction (red circles) was not affected by the addition of KBr or K_2_HPO_4_,^15^ the two inorganic products generated in the reaction mixture (see ESI†). Cumulatively, these results point to a reduction in active catalyst concentration later in the reaction course.

### Identification of resting state of the catalyst

**Scheme 2:**
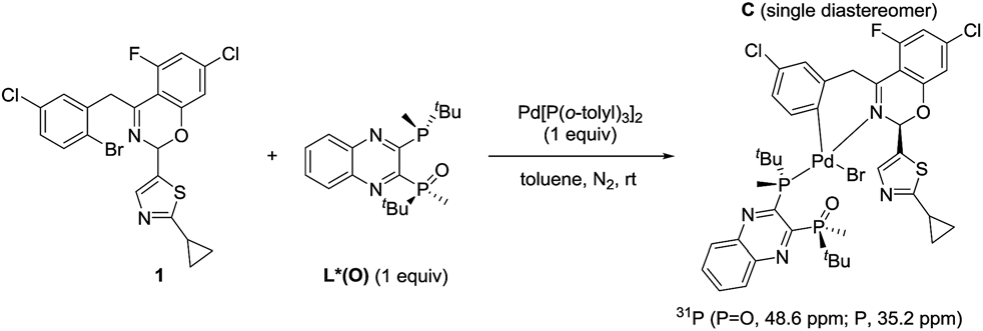
Synthesis of the oxidative addition complex **C**.

**Fig. 4:**
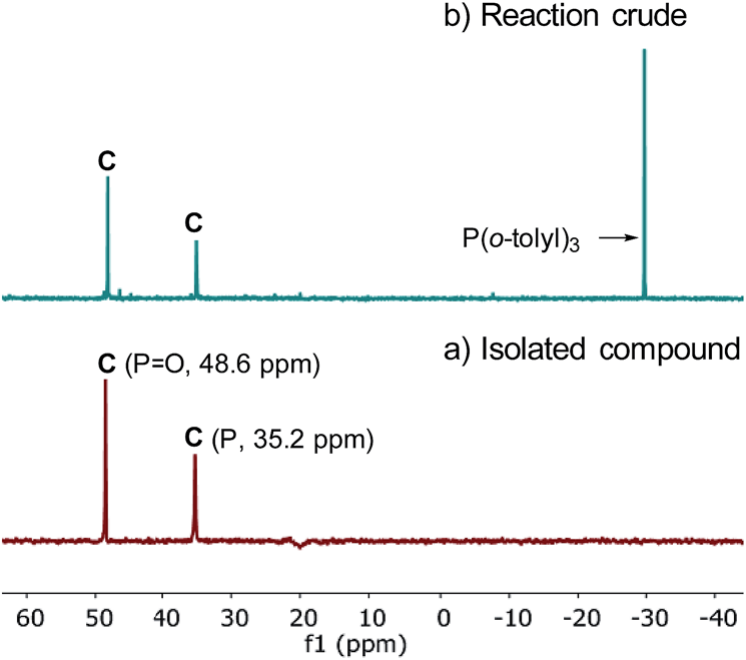
202 MHz ^31^P NMR spectra of (a) the isolated complex **C** and (b) the crude reaction mixture.

The observation of zeroth-order dependence on ArBr **1** suggests that a stable oxidative addition adduct may accumulate and serve as the resting state for the catalytic cycle. To investigate this, we independently synthesized this complex in a stoichiometric reaction with ArBr **1**, (*R*,*R*)-QuinoxP*(O) (L*(O)) and Pd[P(*o*-tolyl)_3_]_2_ (Scheme 2). Interestingly, the crude ^31^P NMR spectrum showed a single pair of phosphorus resonances at *δ* 48.6 and 35.2 ppm, assigned to P=O and P of a single diastereomer of complex **C**, respectively (Fig. 4). Purification by flash chromatography afforded complex **C** as a yellow solid. Unfortunately, we failed to obtain high quality crystals of **C** despite repeated attempts.

**Scheme 3:**
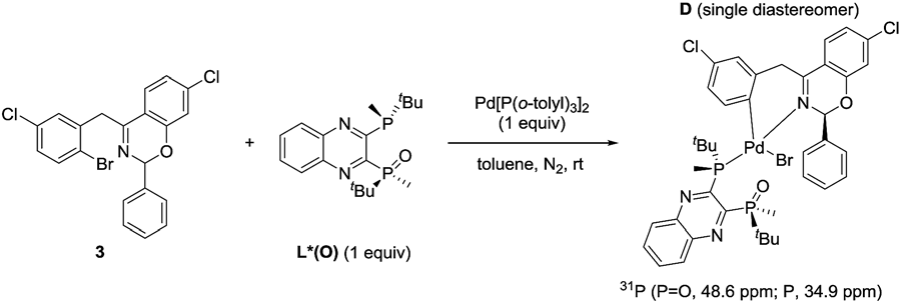
Synthesis of the oxidative addition complex **D**.

**Fig. 5:**
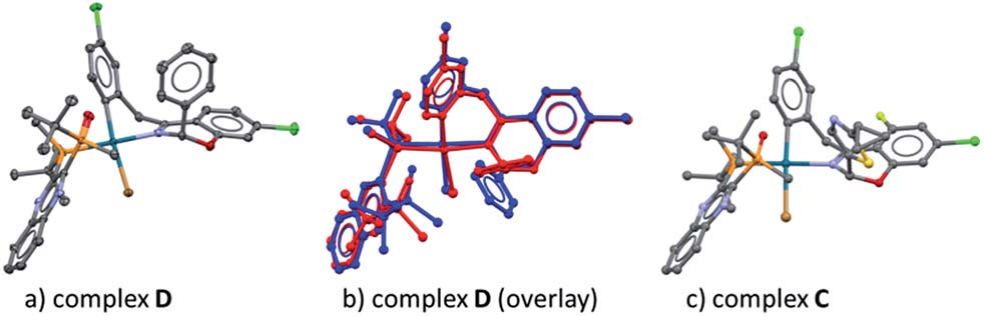
(a) X-ray diffraction structure of complex **D**; (b) overlay between the NMR-DFT modeling (blue) and the X-ray structure (red) of complex **D**; (c) NMR-DFT modeling structure of complex **C**.

In our previous work, we reported that the analogous substrate **3** (Scheme 3) gave the same enantiomer of the corresponding product when subjected to the identical reaction conditions.^9^ Treatment of ArBr **3** with L*(O) and Pd[P((*o*-tolyl)_3_)_2_] also afforded a single pair of characteristic ^31^P NMR resonances at *δ* 48.6 and 34.9 ppm that correspond to the phosphine oxide and phosphine moieties of the BPMO, respectively (see ESI†). Gratifyingly, the isolated complex **D** allowed the growth of X-ray quality single crystals by slow evaporation from toluene. The crystal structure obtained (Fig. 5a) corroborated the structure obtained from NMR spectroscopic and computational modeling analysis (Fig. 5b). In light of these results, we were confident to apply the combination of NMR and DFT modeling techniques to characterize complex **C**, which proved to possess an almost identical structure as complex **D** (Fig. 5c).

**Scheme 4:**
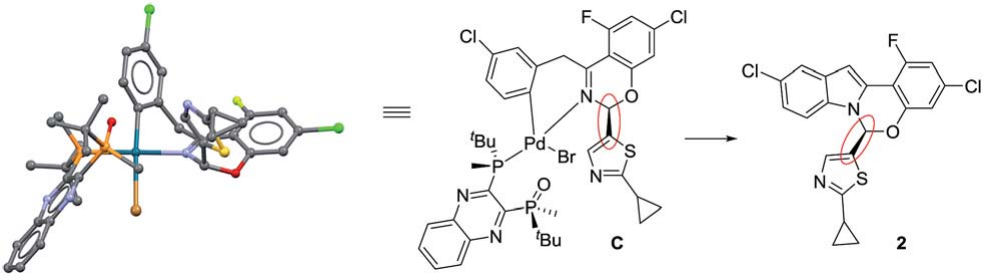
Comparison of complex **C** and product **2**.

These complexes feature the Pd atom in a square planar arrangement, with the arene and bromide in a trans-relationship to each other. The bis-phosphine mono-oxide is directly ligated to the metal center through the phosphorus atom on one side, leaving the phosphine-oxide oxygen 3.125 Å from the Pd center. The imine-type nitrogen is ligated to the metal center, forming a concave boat conformation and, consequently, forcing the nearby rings into proximity. This binding mode facilitates the aromatic group at the hemiaminal position moving to the axial position to avoid the steric repulsion from the bulky ligand. In both complexes **C** and **D**, the observed absolute hemiaminal stereochemistry was consistent with the desired enantiomer of the product **2** (Scheme 4).

### Catalytic competence of complex **C**

**Fig. 6:**
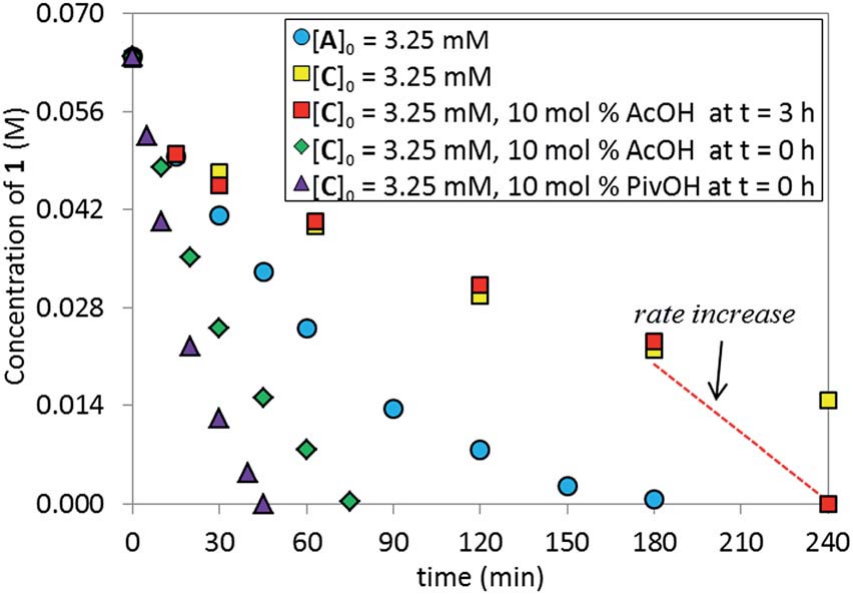
Temporal concentration profiles monitored by ^1^H NMR spectroscopy for the reaction of Scheme 1. [**1**]_0_ = 0.0650 M; [**C**]_0_ = 3.25 mM; [**A**]_0_ = 3.25 mM; 5.5 equiv. of K_3_PO_4_; the amounts of AcOH and PivOH as noted.

We tested the catalytic performance of complex **C** in the standard C–N coupling reaction. Strikingly, complex **C** on its own proved less active (Fig. 6, yellow squares) than the pre-catalyst L*·Pd(OAc)_2_ **A** (blue circles). More interestingly, when the reaction represented by the yellow squares was repeated but 10 mol% of AcOH was added at *t* = 3 h, a dramatic rate increase was observed from that point onward, revealing that *in situ* formed potassium acetate could play an important role in the catalytic cycle. As expected, the addition of 10 mol% AcOH from *t*_0_ provided a higher overall reaction rate (green diamonds) than the standard reaction using the pre-catalyst **A** (blue circles). The use of pivalic acid to generate the more basic and more soluble potassium pivalate *in situ* provided even further rate enhancement (purple triangles). These results highlight the potential role of the carboxylate as an anionic ligand that mediates the intermolecular C–H bond cleavage and serves as a proton shuttle, transferring the hydrogen atom between the substrate and the phosphate base.^16^

**Fig. 7:**
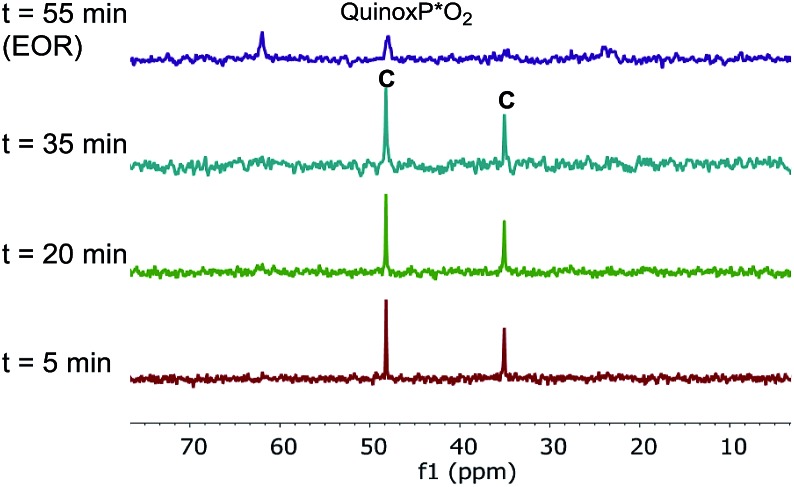
202 MHz ^31^P NMR spectra of aliquots taken at noted times during the reaction of Scheme 1 (complex **C** used as the catalyst, 10 mol% AcOH added). All spectra were recorded by diluting an aliquot of the reaction mixture in toluene-*d*_8_.

**Fig. 8:**
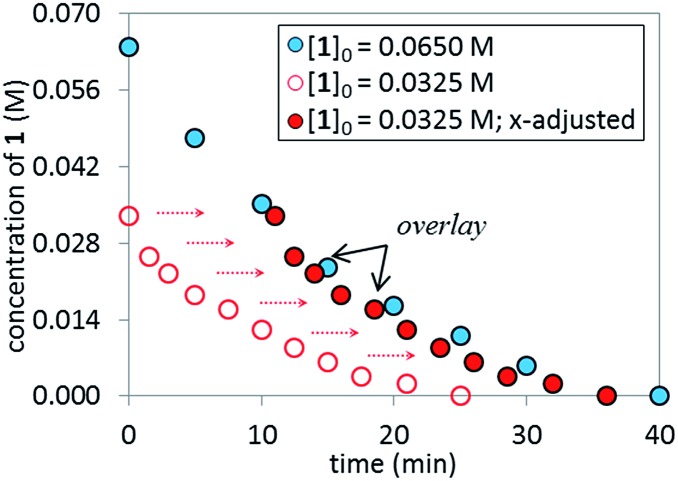
Temporal concentration profiles monitored by ^1^H NMR spectroscopy for the reaction of Scheme 1 using 5 mol% of complex **C** as the catalyst. [**1**]_0_ as noted; [**C**]_0_ = 3.25 mM; 10 mol% PivOH; K_3_PO_4_ (5.5 equiv. based on [**1**]_0_ = 0.0650 M and 11.0 equiv. based on [**1**]_0_ = 0.0325 M).

The superior catalytic performance of complex **C** (in the presence of an appropriate amount of carboxylate) could be an indication of an inefficient activation of pre-catalyst **A**. To investigate this hypothesis, ^31^P NMR spectra for both catalyst systems were compared over the course of the two reactions. As shown in Fig. 7, for complex **C**, a clean ^31^P NMR spectra shows the two characteristic ^31^P resonances were maintained throughout the reaction course. Good superposition from the time-adjusted point for two experiments that differ in initial concentration of substrate but share the same catalyst concentration suggests that neither catalyst deactivation nor product inhibition processes are significant under these conditions (Fig. 8). The kinetic behavior of the catalyst appears to be robust, consistent with the catalyst remaining viable throughout the reaction. It is worth noting that, under these conditions, the reaction appeared to exhibit saturation kinetics in [**1**] with the initial rate of the two reactions only matching up to 20% conversion (see ESI†).

**Fig. 9:**
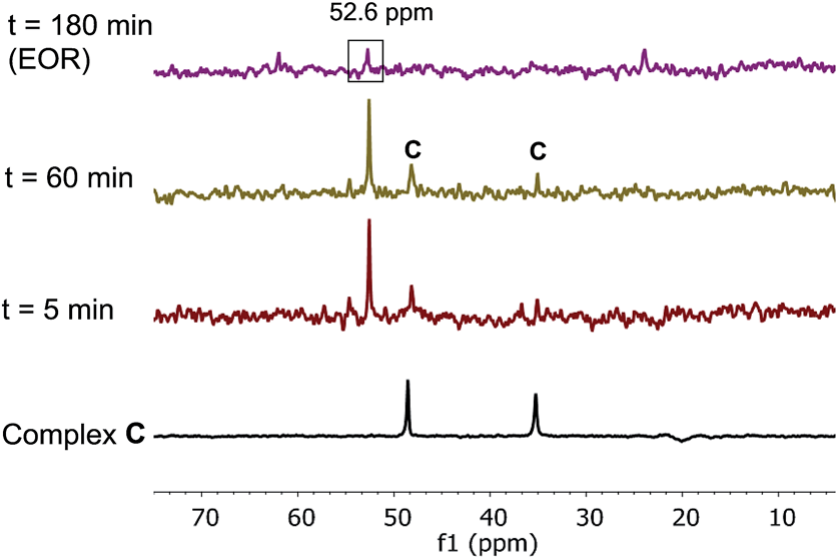
202 MHz ^31^P NMR spectra of aliquots taken at noted times during the reaction of Scheme 1. All spectra were recorded by diluting an aliquot of the reaction mixture in toluene-*d*_8_.

In contrast, we found that pre-catalyst **A**, L*·Pd(OAc)_2_, was only partially activated under standard reactions conditions. In the ^31^P NMR spectrum over the course of the reaction, we observed a minor species showing phosphorus signals at *δ* 48.6 and 34.9 ppm, corresponding to the oxidative addition complex **C** (Fig. 9). A major phosphorus resonance was also observed at 52.6 ppm, very close to the 52.3 ppm resonance in pre-catalyst **A**. This new species showed very high stability under the reaction conditions and was even present at the end of the reaction (*t* = 180 min).^17^

**Fig. 10:**
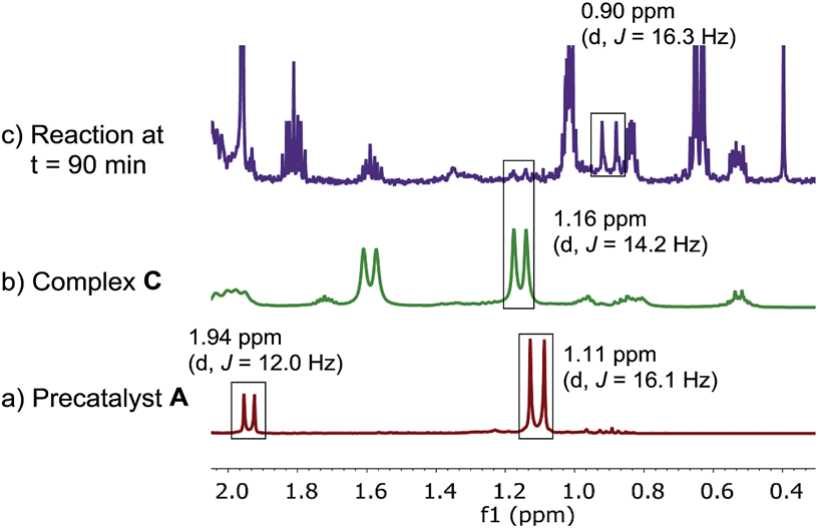
500 MHz ^1^H NMR spectra of (a) pre-catalyst **A**; (b) complex **C**, (c) aliquot taken at *t* = 15 min of reaction at Scheme 1. All spectra were recorded in toluene-*d*_8_.

Because of the ambiguous ^31^P NMR spectra, we turned to the ^1^H NMR (Fig. 10c) to deconvolute the species. We identified the doublet at 1.16 ppm belonging to the *tert*-butyl group of the minor catalytic species, complex **C**. However, the *tert*-butyl resonances of the major palladium species showed different chemical shift (0.9 ppm) *vs.* the pre-catalyst **A** (1.11 ppm). Furthermore, the doublet corresponding to the methyl group of the ligand moiety at 1.94 ppm in the pre-catalyst **A** was not present at the same chemical shift in the spectrum of the reaction mixture.

**Fig. 11:**
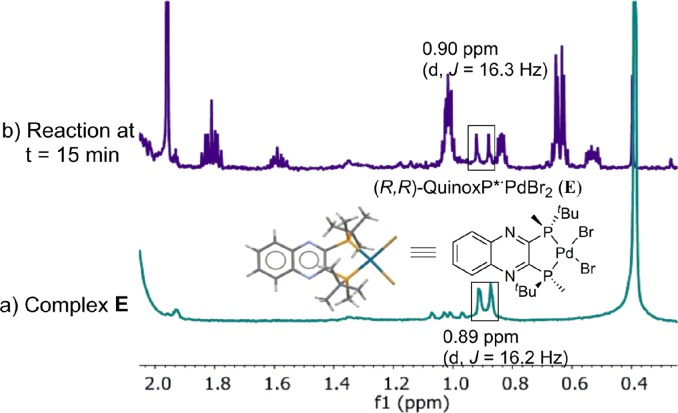
500 MHz ^1^H NMR spectra of (a) L*PdBr_2_, **E**; (b) aliquot taken at *t* = 15 min of reaction at Scheme 1. All spectra were recorded in toluene-*d*_8_.

The high stability allowed us to isolate this persistent species, and the structure was unambiguously established by X-ray diffraction analysis as L*PdBr_2_ **E**,^18^ originating from an anion exchange between acetate and bromide. The newly discovered complex **E** was found to match the major species present in the reaction mixture in both ^31^P (52.5 *vs.* 52.6 ppm) and ^1^H NMR spectra (Fig. 11).

Based on quantitative ^1^H and ^31^P NMR analyses of a standard reaction at *ca.* 20% conversion (see ESI†), the observed species account for >90% of the total phosphorus-containing Pd species and, of that, only 27% is complex **C**. The prevalence of complex **E** likely accounts for the lack of overlay observed between the experiments that were designed to test the catalyst robustness (*vide supra*).

**Fig. 12:**
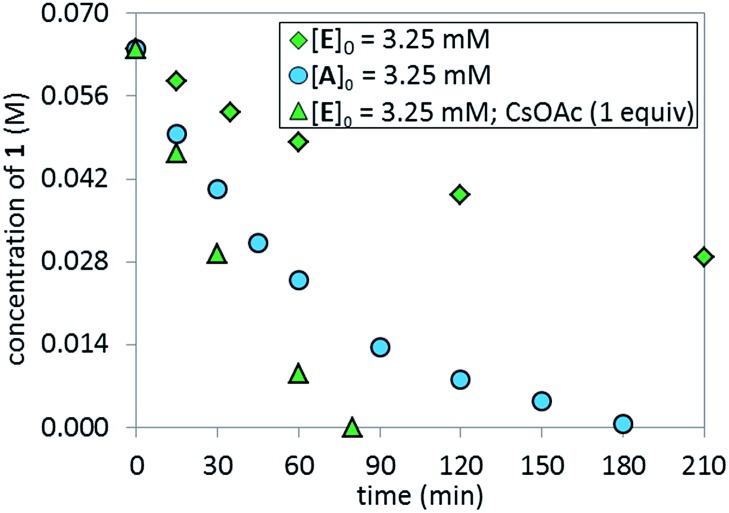
Comparison of temporal concentration profiles monitored by ^1^H NMR spectroscopy for the reaction of Scheme 1 using different catalyst systems, complex **A** *versus* complex **E** (with/without CsOAc). [**1**]_0_ = 0.065 M; [**A**]_0_ = 3.25 mM; [**E**]_0_ = 3.25 mM; 5.5 equiv. of K_3_PO_4_; CsOAc as noted.

**Fig. 13:**
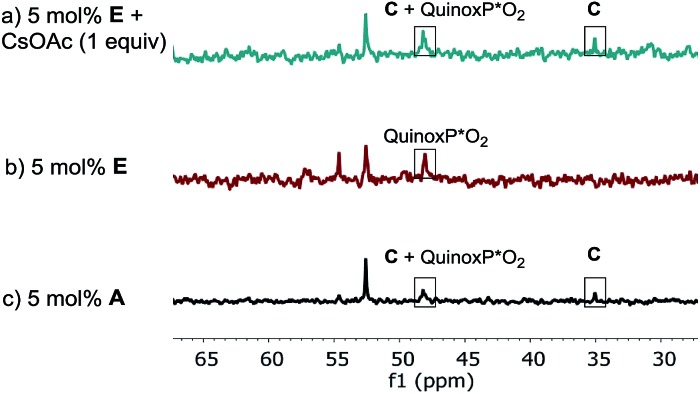
202 MHz ^31^P NMR spectra of aliquots taken at *t* = 15 min for the reaction of Scheme 1: (a) 5 mol% complex **E**; 1 equivalent of CsOAc (b) 5 mol% complex **E**; (c) 5 mol% of pre-catalyst **A**. All spectra were recorded by diluting an aliquot of the reaction mixture in toluene-*d*_8_.

The lower reaction rate obtained with pre-catalyst **A** compared to oxidative addition complex **C** suggests that the formation of complex **E** is detrimental to the reaction. This hypothesis was confirmed by comparing the kinetic profiles obtained using pre-catalyst **E** and **A** (Fig. 12, green diamonds *vs.* blue circles, respectively). The lower reaction rate provided by complex **E** is attributable to its inferior reactivity toward activation to form the active BPMO–Pd(0) complex than pre-catalyst **A**. Notably, when using complex **E** as the catalyst, the addition of an excess of CsOAc (1 equiv. with respect to **1**) to drive the equilibrium from **E** to **A** increased the reaction rate four-fold (green triangles). ^31^P NMR analysis at 15 min indicated a higher concentration of the oxidative addition complex **C**, likely associated with a shift of the equilibrium toward pre-catalyst **A** by CsOAc (Fig. 13).

### Inhibition by free QuinoxP* ligand

The competence of complex **C** in the reaction suggests that generating mono-oxide complex **B** is the key to effective catalysis. Furthermore, strong inhibition was observed when an additional 5 mol% of free (*R*,*R*)-QuinoxP* ligand was added, achieving *ca.* 10% of the desired product formation in 3 hours (see ESI†).^8^

**Scheme 5:**
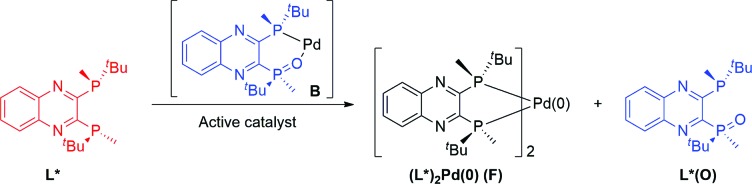
Interaction of active catalyst **B** with free ligand (*R*,*R*)-QuinoxP*.

**Fig. 14:**
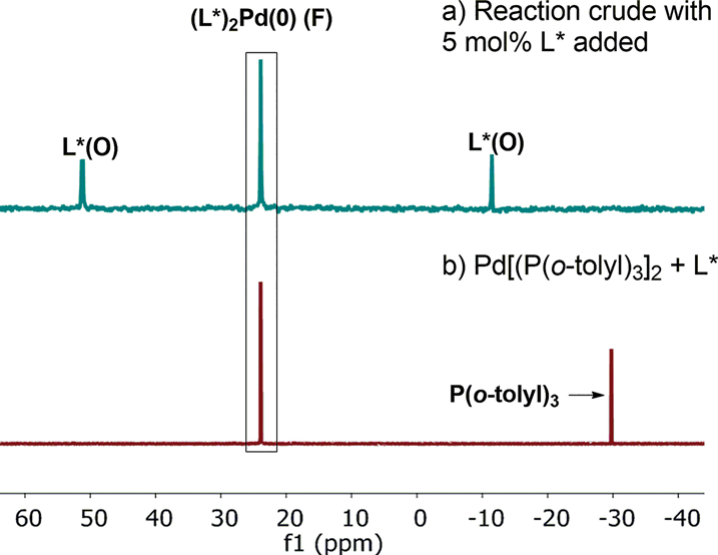
202 MHz ^31^P NMR spectra of (a) aliquot taken at *t* = 180 min of Scheme 1 in the presence of excess 5 mol% of L*; (b) Pd[P(*o*-tolyl)_3_]_2_ : L* (1 : 1). All spectra were recorded in toluene-*d*_8_.

^31^P NMR analysis of the reaction mixture under these conditions showed that species **C** is not present, while ligand L*(O) and a new species identified by mass spectroscopy as (L*)_2_Pd(0) (**F**) were formed (Fig. 14a). Free L* readily displaces L*(O) to produce inactive species **F** (Scheme 5) that could also be formed upon simple treatment of L* and Pd[P(*o*-tolyl)_3_]_2_ (Fig. 14b).

### Rate-determining step/kinetic isotopic effect (KIE)

**Scheme 6:**
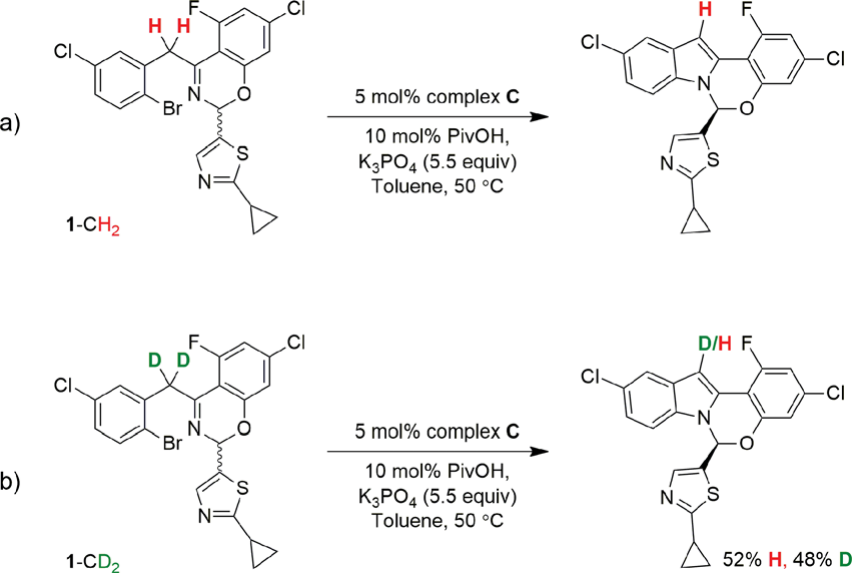
Parallel kinetic isotopic experiments.

**Fig. 15:**
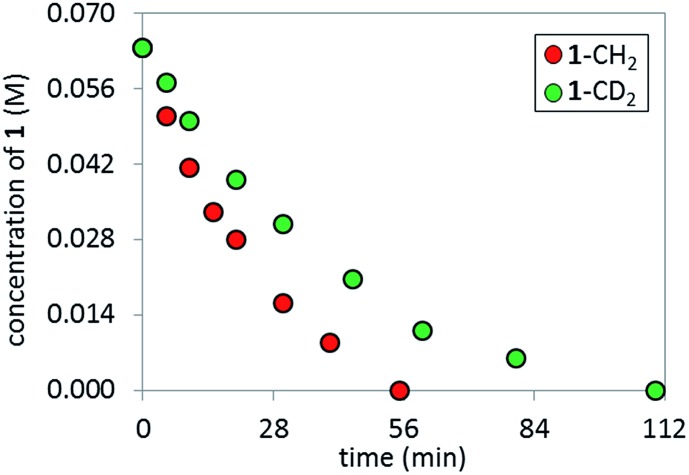
Temporal product concentration profiles monitored by ^1^H NMR spectroscopy for the reaction carried out by using H- and D-**1** at the benzylic position in separate reactions [**1**-CH_2_]_0_ = 0.065 M; [**1**-CD_2_]_0_ = 0.065 M; [**C**]_0_ = 3.25 mM; 10 mol% PivOH and 5.5 equiv. of K_3_PO_4_.

In order to perform KIE studies, we prepared compound **1**-CD_2_ that was bis-deuterated at the benzylic methylene position. Under standard reaction conditions but in the absence of catalyst, the deuterium atoms at the benzylic position proved labile, with *ca.* 32% proton^19^ incorporation within 3 h by ^1^H NMR spectroscopy (see ESI†). Despite the lability of these protons, the parallel reactions (Scheme 6) still provided a *k*_H_/*k*_D_ ratio of 1.8 from initial rates (Fig. 15). The observed kinetic isotope effect is in accordance with the positive kinetic order in [K_3_PO_4_] as well as with the rate acceleration produced by the addition of carboxylates. Furthermore, when carrying out the reaction using complex **C** as catalyst, the replacement of K_3_PO_4_ by K_2_HPO_4_ as base proved detrimental (see ESI†). These data suggest that the formation of the complex **C** precedes an irreversible, rate-limiting step that involves C–H bond cleavage.^20^

### Enantio-determining step

**Scheme 7:**
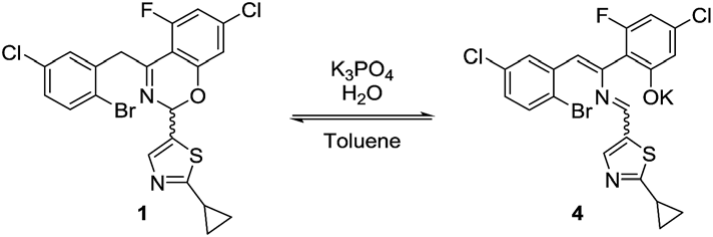
Racemization of **1** through open form **4**.

**Fig. 16:**
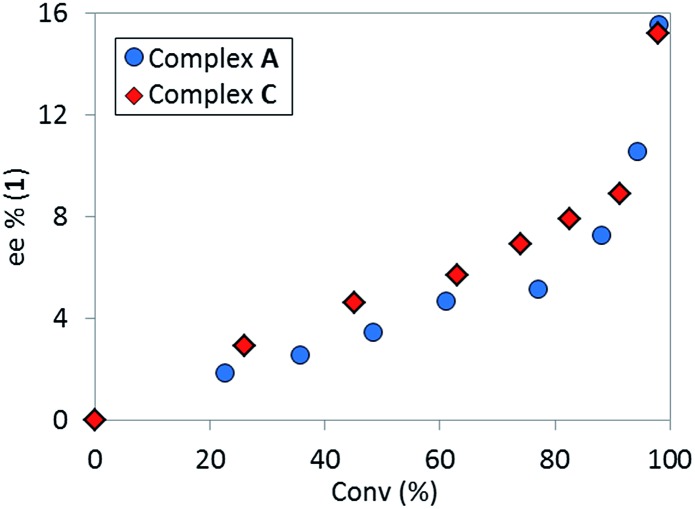
Temporal enantioselectivity of the remaining substrate **1** *versus* reaction conversion for the reaction in Scheme 1 using pre-catalyst **A** or complex **C** as catalyst.

The high diastereoselectivity of the oxidative addition complex observed by ^31^P NMR spectroscopy appears consistent with the formation of complex **C** as the enantio-determining step of the catalytic cycle. Although racemization of ArBr **1** through the open form **4** (Scheme 7) is induced by the base under reaction conditions, we observed a slight enantiomeric enrichment of the remaining starting material over time, reaching 16% ee at around 95% conversion (Fig. 16). This supports the proposed preference of the active catalyst **B** toward one enantiomer of the starting aryl bromide. A positive order in the concentration of ArBr **1** at earlier conversion when using a greater concentration of active catalyst (*i.e.* using 5 mol% of complex **C**, see ESI†) further supports this argument. With a higher concentration of active catalyst **B**, progression of the reaction starts depleting the concentration of the productive (*S*)-**1**, causing the reaction to become positive order in **1** since the rate of racemization is not fast enough to maintain a racemic mixture.

**Fig. 17:**
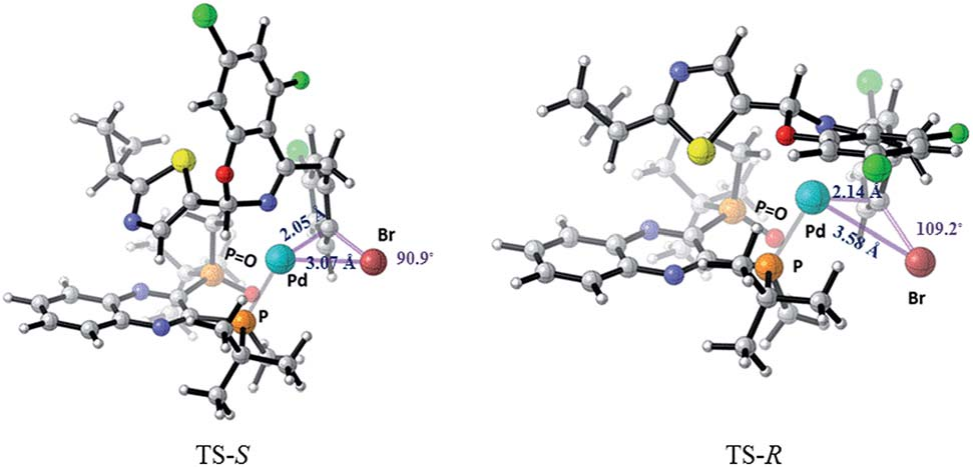
Transition states for the oxidative addition step in formation of **C** and *R*-**C**.

Computational studies^21^ of the formation of complex **C** were carried out using M06/6-31+G(d) with SDD for Pd and Br in Gaussian 09. Solvation in toluene was accounted for with the SMD solvent model (Fig. 17). The ΔΔ*G* ^‡^ (*ca.* 1.5 kcal mol^–1^) favors the formation of the desired complex **C** (*S* configuration at the hemiaminal position) over the undesired diastereomer (*R*-**C**). Complex **C** is also favored thermodynamically over the undesired diastereomer *R*-**C** by 3.67 kcal mol^–1^. The *R*-**C** substrate must undergo re-puckering at the benzylic methylene in order to alleviate steric clashes during formation of the oxidative addition complex. As a result, the TS-*R* is a later transition state than TS-*S*, and can be described by longer distances and wider angles between the reacting atoms. The Pd–C bond being formed is 2.14 Å in the TS-*R* and 2.05 Å in TS-*S*, and the Pd–Br bond being formed is 3.58 Å in TS-*R* and only 3.07 Å in TS-*S*. Also, the Br–C–Pd angle is sharper in the TS-*S* than the TS-*R*, with angles of 90.9° and 109.2° respectively.

**Scheme 8:**
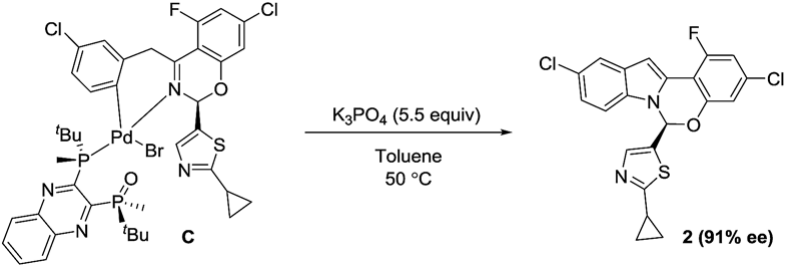
Stoichiometric synthesis of product **2** from the oxidative addition complex **C**.

**Fig. 18:**
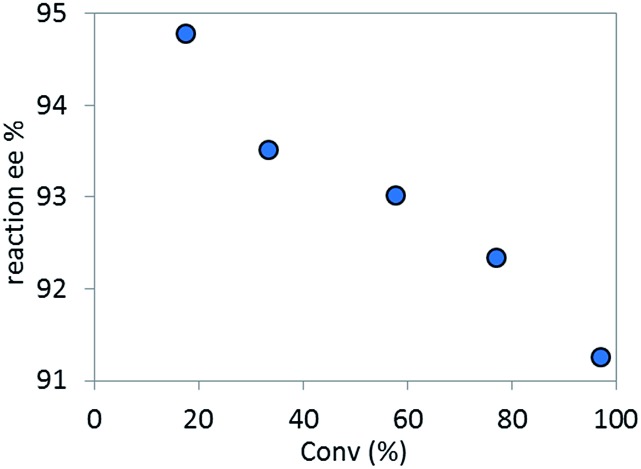
Temporal reaction ee monitored over time for the stoichiometric reaction of complex **C** with 5.5 equivalents of K_3_PO_4_.

Based on the high diastereoselectivity observed experimentally, correspondingly high product ee (>98%) would be expected. However, 91% ee is obtained under catalytic conditions. Further, reacting a single diastereomer of complex **C** with 5.5 equivalents of K_3_PO_4_ at the standard reaction temperature (50 °C, Scheme 8), gave a slight product ee erosion (from 95% ee^22^ to 91% ee at *ca.* 10% and 98% conversions, respectively) (Fig. 18). Notably, a very similar ee reduction was also observed under standard catalytic conditions (see ESI†). Product **2** was shown to be configurationally stable to base at the reaction temperature (see ESI†). Therefore, the ee erosion should derive from some downstream catalytic step following the formation of complex **C**.

**Scheme 9:**
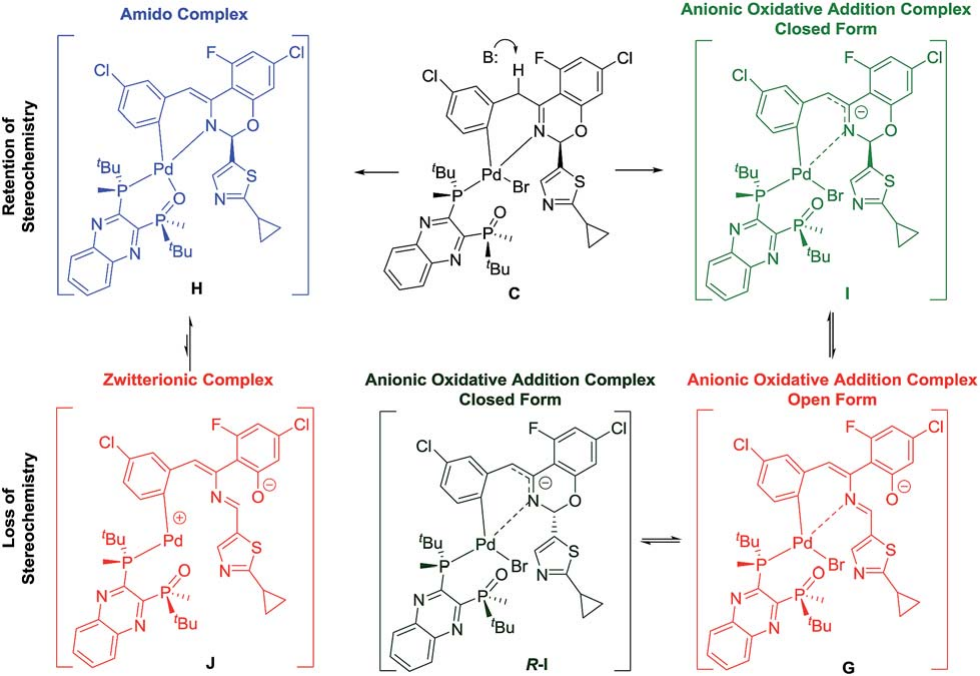
Different deprotonation pathways of complex **C**.

**Scheme 10:**
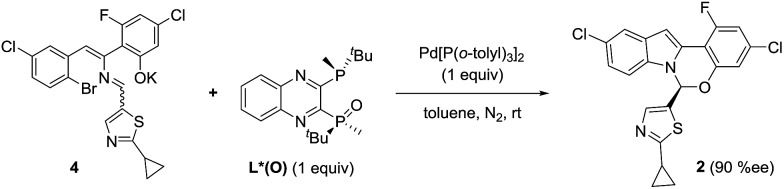
Product **2** formation from open form **4**.

Two plausible intermediates could be derived from the deprotonation of complex **C** (Scheme 10), namely: (a) complex **H** with retention of the stereochemistry at the hemiaminal position, from which stereochemical integrity is maintained and/or; (b) complex **G** in which the stereochemical information at the hemiaminal center is lost through ring opening. Along the deprotonation pathway, DFT calculations indicated a stable intermediate, complex **I** (Scheme 9), could be formed where C–H bond cleavage has occurred but the bromide remains bound. Calculations of interconversion through the open form **G** indicated both **C** and *R*-**C** were accessible, with **I** being thermodynamically preferred relative to *R*-**I** when closing from the open form **G** (Table 1). Furthermore, our simulations showed that while complex **G** is stable and energetically accessible, interconversion between the **C** and *R*-**C** form through the zwitterion **J** is less likely due to the high thermodynamic penalty (Table 1) and dramatic structural rearrangement required.

**Table 1:** Computationally determined free energy (kcal mol^–1^) difference between lowest-energy intermediate pairs in both the gas-phase or in presence of toluene

Complex interconversion	ΔΔ*G* ^r^ (kcal mol^–1^) gas phase	ΔΔ*G* ^r^ (kcal mol^–1^) toluene
**H** → **J**	+20.85	+17.27
**I** → **G**	+10.27	+11.60
**I** → *R*-**I**	+3.71	+4.95
**I**(K^+^) → **G**(K^+^)	–1.43	–6.49

In order to examine if complex **G** could be a competent intermediate, we treated the open form **4** with Pd[P(*o*-tolyl)_3_]_2_ and L*(O) (Scheme 10). The reaction provided the desired product in 90% ee. Presumably, the transformation occurred through the intermediacy of complex **G**. To account for the high observed enantioselectivity, the ring closure of complex **G** leading to **H** to allow the reductive elimination to take place should occur with high diastereoselectivity, presumably aided by the stereochemical information relay from the chiral ligand. Notably, since no proton abstraction was required, the reaction occurred instantaneously at room temperature.

Based on the above results and in consonance with the slight reaction ee change as the reaction progresses, two different enantio-determining steps, namely, the diastereoselective formation of complex **C** and the ring closure of complex **G** to form complex **H**, appear operational.

While the formation of **C** is expected to be highly diastereoselective, the possible competing side reactions representing the formation of **G**, either directly from **C** or through trapping of **B** by the open form **4**, likely explain the observed lower ee. Our computational studies further supported the feasibility of both pathways.

### Design of improved pre-catalysts

**Scheme 11:**
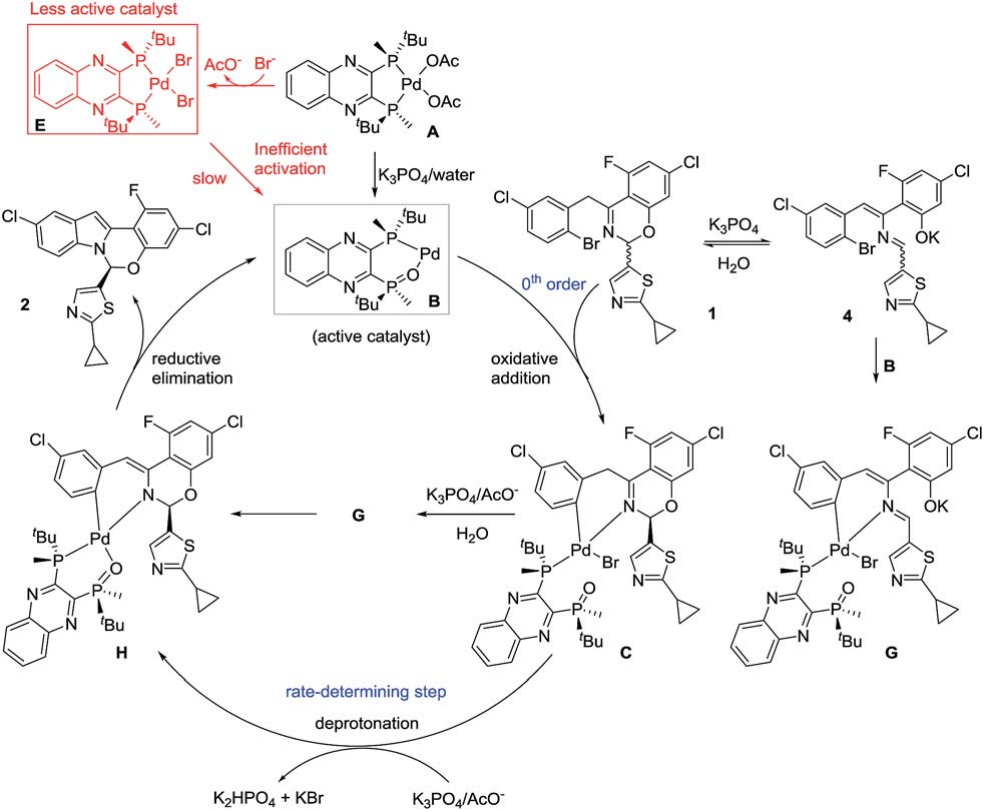
Proposed mechanism for the reaction of Scheme 1 highlighting critical species and catalyst poisons.

Based on the collective mechanistic insights from the above studies, a full mechanistic proposal is summarized in Scheme 11. Inefficient activation of pre-catalyst **A** to form active catalyst **B** largely leads to formation of complex L*·PdBr_2_ **E** and <30% of starting Pd entering the catalytic cycle. Prevention of the formation of complex **E** and quantitative generation of active catalyst **B** appear critical for reducing the catalyst loading and increasing robustness of this asymmetric catalytic reaction. The undesired pathway leading to complex **E** would be avoided if the oxidative addition complex **C** were used as the pre-catalyst. However, the formation of complex **C** from L*(O), Pd[P(*o*-tolyl)_3_]_2_ and ArBr **1** is inefficient, albeit under unoptimized conditions (40% yield). More significantly, L*(O) was synthesized from the free ligand L* in a 3-step sequence with an overall 67% isolated yield.^9^ Therefore, any advantage gained in catalytic performance from the oxidative addition complex **C** would be offset by its tedious preparation.

Active catalyst **B** could be formed from a mixture of free ligand L*, Pd(OAc)_2_, water and base, but the complex proved unstable and quickly decomposed. Alternatively, trapping with ArBr **1** was complicated by cyclization of complex **C** in the presence of base preventing its isolation. Based on this knowledge, we envisioned that we could use simple aryl halides to trap active catalyst **B** as stable oxidative addition complexes that would only release active L*(O)–Pd(0) catalyst **B** after a promoted reductive elimination.

**Scheme 12:**
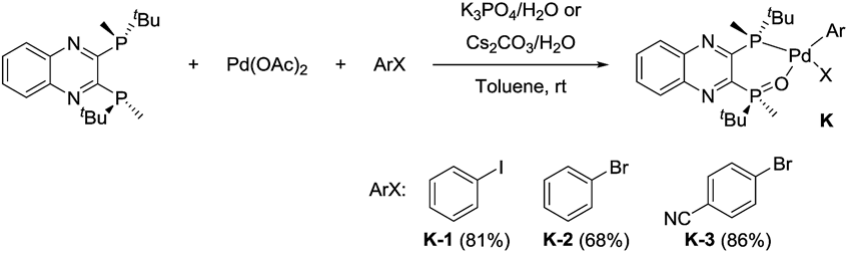
Synthesis of L*(O)–PdArX complexes **K**.

We identified a series of aryl halides with relatively high reactivity toward oxidative addition,^23^ including iodobenzene, bromobenzene and *p*-bromobenzonitrile, that cleanly afforded the desired oxidative complexes **K** ^24^ (Scheme 12). These complexes were shown to be air-stable and could be purified by silica gel chromatography. Chlorobenzene failed to undergo oxidative addition. The introduction of an electron-withdrawing substituent (*i.e.* nitrile) increased the reactivity with respect to bromobenzene and afforded the corresponding oxidative addition complex (**K-3**) in an increased 86% yield on 10 g scale. The installation of a *para*-nitrile group also allowed for direct isolation by crystallization from toluene, a highly desired feature from a practical perspective.

**Scheme 13:**
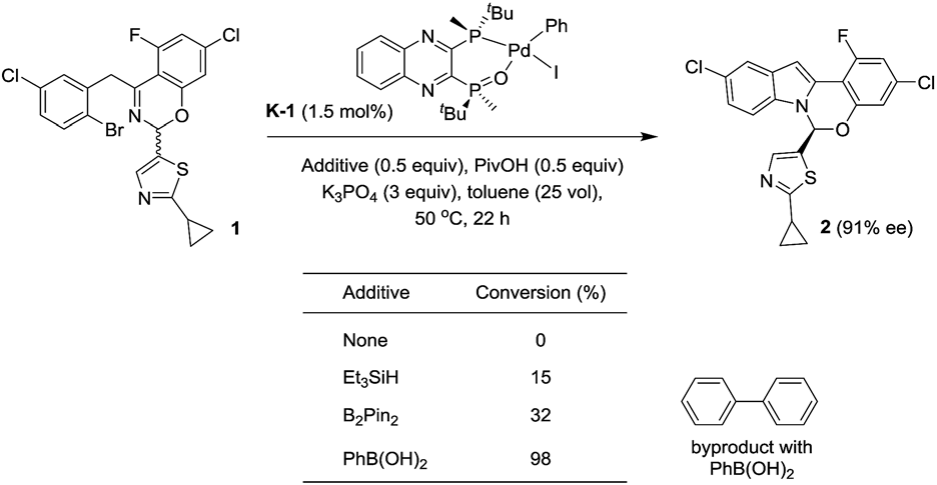
Activation of the pre-catalyst **K-1**.

With these newly synthesized oxidative addition complexes in hand, we evaluated their performance as pre-catalysts in the standard C–N coupling reaction (Scheme 13). As expected, utilizing iodobenzene complex **K-1** as catalyst, in the absence of additive, failed to produce any product. Using triethylsilane as a reductant, 15% conversion was obtained at 1.5 mol% catalyst loading, providing proof of concept for our activation hypothesis. Higher conversion (32%) was observed on introduction of bis-pinacolborane, and employing of phenylboronic acid proved optimal, giving 98% conversion. The observation of biphenyl formation (1.5 mol%, in accordance with the catalyst loading) in the latter case confirmed activation proceeded *via* Suzuki–Miyaura coupling. In accord with our hypothesis, the use of these pre-catalysts did not alter the enantioselectivity of the reaction, and we observed the expected oxidative addition complex **C** during the course of the reaction.

Unfortunately, the use of phenylboronic acid as a reductant led to low reaction conversion at lower catalyst loading (11% conversion at 0.3 mol%). Therefore, we screened a range of arylboronic acids with the *p*-bromobenzonitrile complex **K-3** to identify a superior additive (see ESI†). Both steric and electronic properties of the boronic acid were shown to play critical roles in the efficiency of the activation. *o*-Tolylboronic acids bearing electron-donating groups at the *para*-position to increase nucleophilicity of the boronic acids^25^ were found to be optimal. Utilizing 0.3 mol% of the pre-catalyst **K-3**, 84% conversion to product was achieved after 28 h in the presence of 4-methoxy-2-tolylboronic acid.

**Scheme 14:**
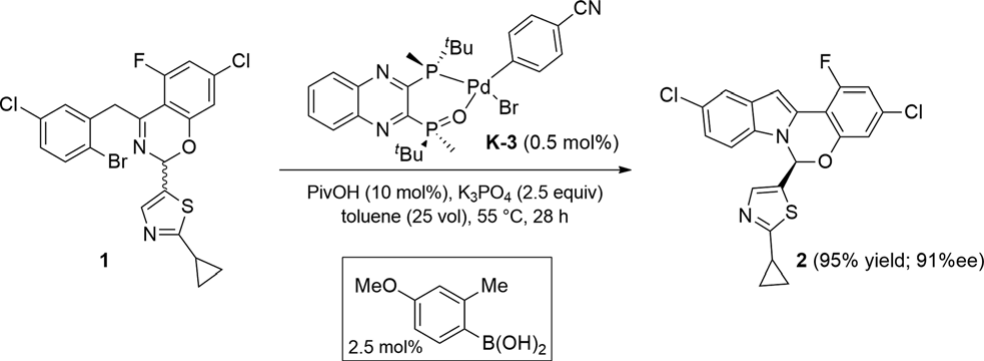
Improved reaction conditions.

The use of these new series of Pd(ii) pre-catalysts activated with addition of arylboronic acids provides the highest catalytic performance, delivering maximal on-cycle palladium in the active catalyst form L*(O)–Pd(0) **B**. Notably, as was expected from our previous kinetic data, PivOH addition was also required to achieve the desired reactivity. Using pre-catalyst **K-3** and 4-methoxy-2-tolylboronic acid as additive, 100% conversion to the desired product was achieved with as low as 0.5 mol% catalyst loading, a 10-fold reduction in catalyst loading while maintaining the required high product enantioselectivity (Scheme 14).

## Conclusions

Our mechanistic studies related to a dynamic asymmetric C–N coupling revealed an inefficient pre-catalyst activation process to convert the inactive Pd(ii)–bis-phosphine complex into the active Pd(0)–BPMO species. Given the integral design element required for hypothesis-driven high throughput experimentation, the guarantee of pre-catalyst activation is paramount. The incompetent pre-catalyst activation in our chemistry established a critical need to develop a superior entry into Pd–BPMO catalysis. We envisioned that improving this particular reaction would have broad impact on the field of catalysis. With this in mind, we have developed a new class of air-stable bis-phosphine mono-oxide Pd pre-catalysts (BPMO–Pd(ii)) that are easily prepared *via* oxidative addition from the readily available bis-phosphine ligand, Pd(OAc)_2_, and any of a series of aryl halides.^26^ In order to deliver BPMO–Pd(0) from these pre-catalysts, we discovered an efficient catalyst activation protocol *via in situ* Suzuki–Miyaura reaction with an arylboronic acid partner. This new pre-catalyst design successfully addressed the inefficient *in situ* mono-oxidation of the bis-phosphine ligand to BPMO, protected it from anionic ligand exchange with halide, and controlled the ligand/Pd ratio. The system under study greatly benefited from the use of these new pre-catalysts, achieving an impressive 10-fold reduction of catalyst loading from 5 to 0.5 mol% while maintaining the same yield and enantioselectivity. The use of these rationally designed pre-catalysts also provided improved process robustness, with a significant impact on the overall reaction cost and the sustainability of the chemical transformation. Furthermore, the development of this simple protocol obviates the need for a separate synthesis and isolation of BPMO ligands^27^ and provides efficient access to a new class of BPMO–Pd(ii) pre-catalysts.^28^ The facile accessibility of these pre-catalysts will enable their explicit screening affording a better assessment of their inherent reactivity, especially in HTE format, and could lead to the discovery of novel asymmetric transformations.
