# Supplementary material for: A rational pre-catalyst design for bis-phosphine mono-oxide palladium catalyzed reactions
Source: Chem Sci. 2017 Jan 19;8(4):2841–51. doi: 10.1039/c6sc05472b (PMC5427683; doi:10.1039/c6sc05472b)
Supplement: Supplementary file 1 [file SC-008-C6SC05472B-s001.pdf]

Electronic Supplementary Material (ESI) for Chemical Science.
This journal is © The Royal Society of Chemistry 2017

Supporting Information

A Rational Pre-Catalyst Design for Bis-Phosphine Mono-Oxide Palladium Catalyzed Reactions

Yining Ji,* Hongming Li, Alan M. Hyde, Qinghao Chen, Kevin M. Belyk, Katrina W. Lexa, Jingjun Yin, Edward C. Sherer, Robert T. Williamson, Andrew Brunskill, Sumei Ren, Louis-Charles Campeau, Ian W. Davies and Rebecca T. Ruck

Department of Process Research & Development, Merck & Co. Inc., Rahway, New Jersey 07065, United States

E-mail: yining.jichen@merck.com

Contents:

Contents	Page
1. General remarks	S-2
2. General experimental procedure for kinetic experiments	S-3
3. Kinetic data with (*R,R*)-QuinoxP*Pd$(OAc)_2$ **A**	S-4
4. Kinetic data with complex **C**	S-9
5. ^{1}H NMR spectra over reaction course for the different catalyst systems	S-12
6. Quantification of catalytic species for the standard reaction at t = 15 min	S-14
7. Stereochemical stability of the product **2**	S-15
8. Synthesis of ((*R,R*)-QuinoxP*$)_2$Pd(0) **F**	S-16
9. Screening of boronic acids using pre-catalyst **K**-3	S-17
10. Kinetic data with substrate **3**	S-18
11. Buchwald G3/G4 type precatalyst with QuinoxP*(O)	S-20
12. Experimental procedures and Characterization	S-21
13. ^{1}H and ^{13}C NMR spectra	S-32
14. X-ray crystallography data	S-45
15. Structural determination of complex **C** and **D** by NMR	S-52
16. DFT calculations	S-66

1. General remarks

Solvents: Solvents were purchased from Sigma Aldrich, anhydrous, sure-seal quality, and used with no further purification. Chloroform-d (d, 99.8%) was purchased from Cambridge Isotope Laboratories, Inc. Toluene-d_8 (d, +99%) and dichloromethane-d_2 (d, 99.8%) were purchased from Acros Organics.

Materials: All reagents were used as purchased from commercial suppliers. The ligands and bases were purchased from commercial sources.

Methods: All experiments described were carried out under a nitrogen atmosphere inside of a MBraun glove box operating with a constant N_2-purge.

Analysis: SFC Analysis was conducted on a Waters Acquity UPC2 instrument. Mettler-Toledo AX-204 analytical balance was used for the weighings. Reactions were monitored using a Waters Acquity UPLC-MS equipped with an SQD detector with electrospray S4 ionization (ESI) source in the positive mode. High resolution mass spectra were recorded on a Xevo G2 QTof (Waters MS Technologies) mass spectrometer by electrospray ionization. Optical rotations were measured on an Autopol V plus automatic polarimeter by Rudolph Research Analytical, and data are reported as $[\alpha]_D^{25}$ (concentration in g/100 mL, solvent).

^{1}H, ^{13}C NMR, ^{19}F and ^{31}P NMR spectra were acquired on Bruker DRX-500 and AMX-400 instruments, and calibrated using residual non-deuterated solvent as an internal reference. The following abbreviations (or combinations thereof) are used to explain the multiplicities: s = singlet, d = doublet, t = triplet, m = multiplet, quin = quintuplet, br = broad. In order to obtain quantitative data we measured the 90 degree flip angle (P1/4) and the longitudinal relaxation time (T1) for trimethoxybenzene in toluene. Both **1** and **2** have relaxation times longer than 2.7 s (relaxation time for the CH signal of the trimethoxybenzene in toluene). Therefore, in the kinetic studies, to assure that all signal integrations are quantitative, a relaxation delay (**D1**) of 15 s was used.

2. General experimental procedure for kinetic experiments

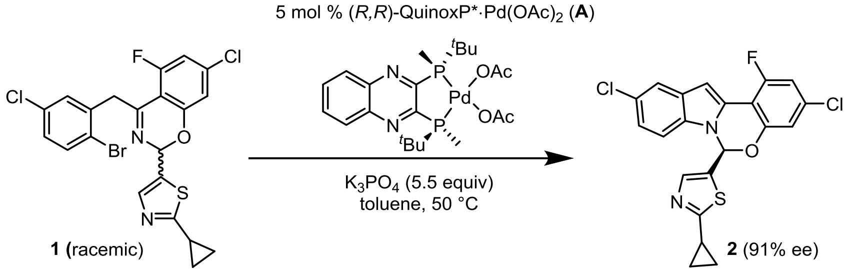

Standard Conditions: To a 2 mL volumetric flask was charged **1** (80.7 mg) and trimethoxybenzene (10 mg). The mixture was dissolved with pre-degassed toluene. This stock solution (1.65 mL) was charged into an 8 mL vial containing 151.7 mg of K_3PO_4. The vial was then placed in a preheated plate at 50 °C. The reaction was initiated by addition of 0.35 mL of a stock solution of (*R,R*)-QuinoxP*·Pd(OAc)$_2$ pre-catalyst **A** (10.4 mg).

Reaction progress was monitored by ^{1}H NMR spectroscopy analysis of withdrawn aliquots. The reaction was sampled by withdrawing of ca. 50 μL of the reaction solution and diluting with 0.4 mL of toluene-d_8. The final reaction concentration of **1** and **2** were determined by ^{1}H NMR spectroscopy analysis.

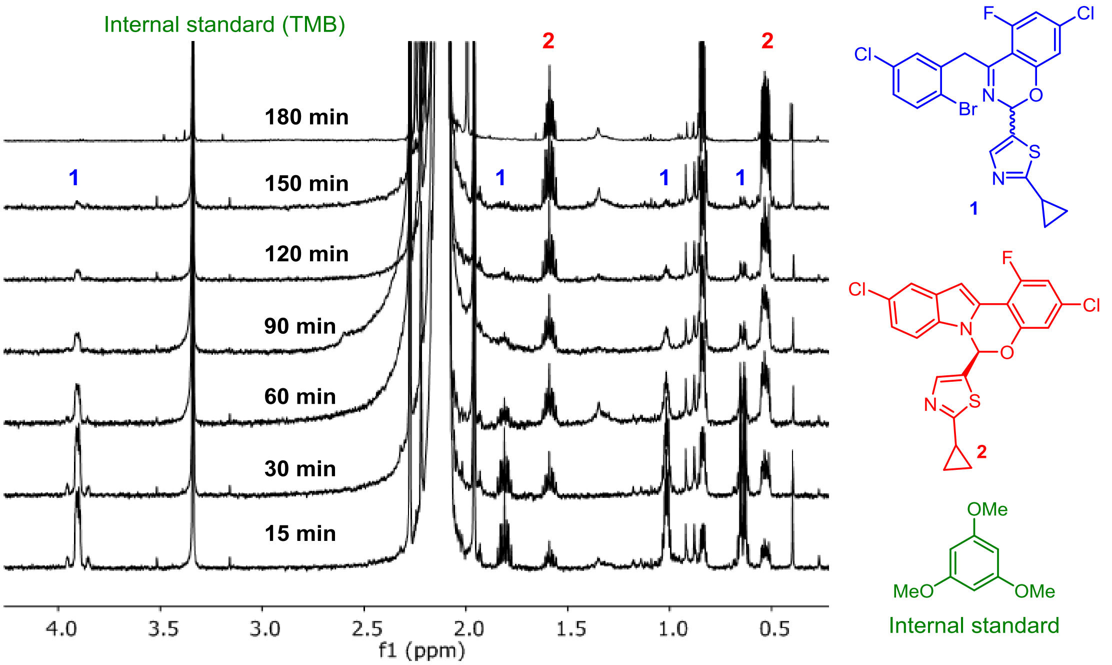

Fig. S1. 500 MHz ^{1}H NMR spectra of samples taken at different time intervals over the reaction course at standard reaction conditions.

3. Kinetic data with (*R*,*R*)-QuinoxP*·Pd(OAc)$_2$ A

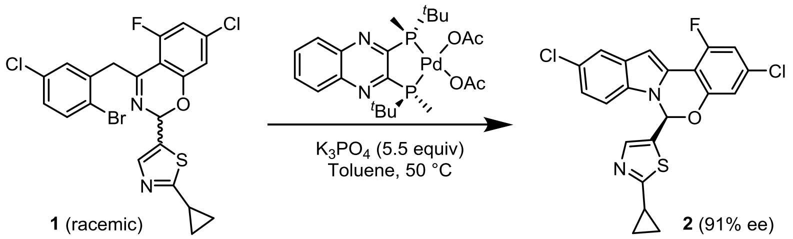

Zero order kinetics in [1]

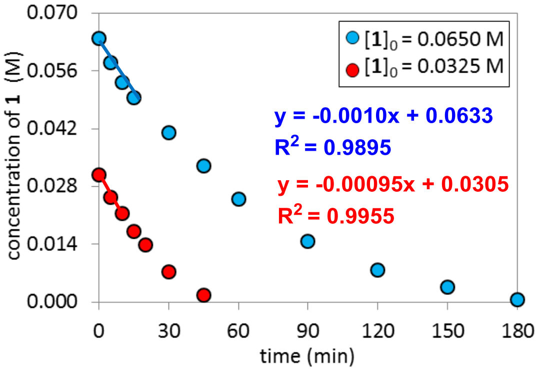

Fig. S2. Temporal concentration profiles monitored by ^{1}H NMR spectroscopy for the reaction of Scheme 1. [**1**]$_0$ as noted; [**A**]$_0$ = 3.25 mM; 5.5 equivalents of K_3PO_4 (based on [**1**]$_0$ = 0.065 M). The similar initial rates obtained for the two reactions provided a zero-order kinetics in [**1**].

Positive order kinetics in [K_3PO_4]

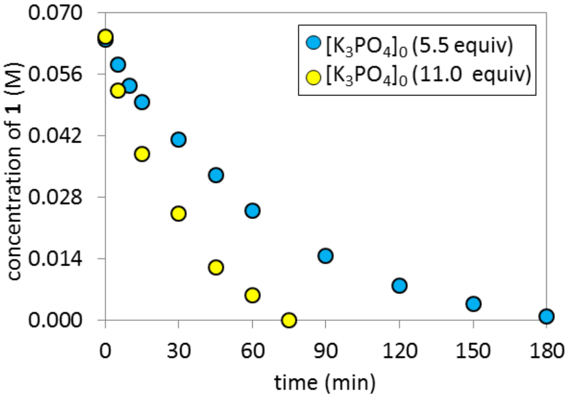

Fig. S3. Temporal concentration profiles monitored by ^{1}H NMR spectroscopy for the reaction of Scheme 1. [**1**]$_0$ = 0.065 M; [**A**]$_0$ = 3.25 mM; equivalents of K_3PO_4 as noted. Positive order in [K_3PO_4] has been observed since higher amount of K_3PO_4 produced faster consumption of **1** (yellow circles).

First order kinetics in [(R,R)-QuinoxP*·Pd(OAc)$_2$] A

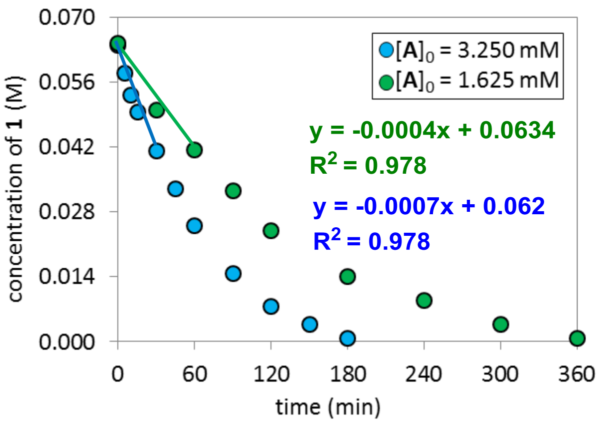

Fig. S4. Temporal concentration profiles monitored by ^{1}H NMR spectroscopy for the standard reaction. [**1**]$_0$ = 0.065 M; 5.5 equivalents of K_3PO_4; [**A**] as noted. The initial rates for the two reactions provided a first-order kinetics in [**A**].

Product inhibition experiments

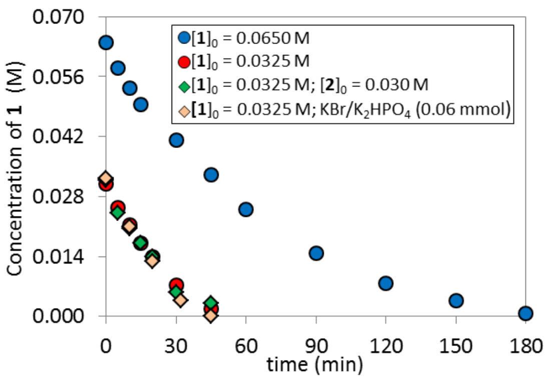

Fig. S5. Temporal concentration profiles monitored by ^{1}H NMR spectroscopy for the standard reaction. [**1**]$_0$ as noted; [**A**] = 3.25 mM; 5.5 equivalents of K_3PO_4 (based on [**1**]$_0$ = 0.065 M). Product addition conditions: [**1**]$_0$ = 0.0325 M, [**2**]$_0$ = 0.030 M and [**1**]$_0$ = 0.0325 M, KBr/K_2HPO_4 (0.06 mmol). No product inhibition was observed since the reaction rate was not altered by the addition of either **2** or a mixture of KBr/K_2HPO_4.

Deuterium loss of 1·CD_2 under reaction conditions.

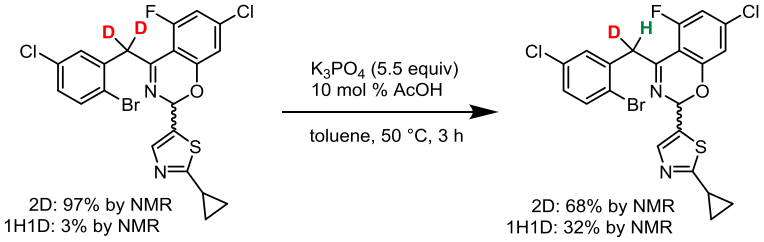

Parallel kinetic isotopic experiments

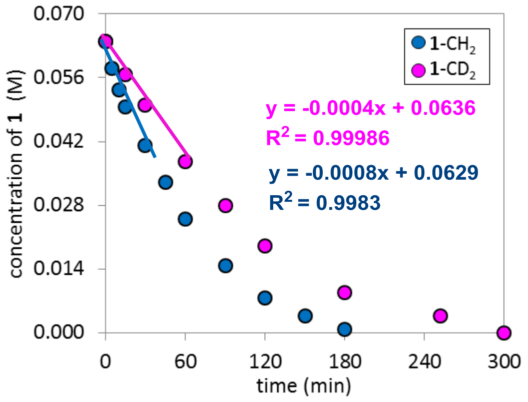

$$\mathrm{KIE} = \frac{Rate\ \mathbf{1}\cdot\mathrm{CH_2}}{Rate\ \mathbf{1}\cdot\mathrm{CD_2}} = \frac{0.0008}{0.0004} = 2$$

Fig. S6. Temporal concentration profiles monitored by ^{1}H NMR spectroscopy for the standard reaction carried out by using **1**·CH_2 and **1**·CD_2 in separate reactions vessels. Reaction conditions: [**1**·CH_2]$_0$ = 0.065 M (blue circles) and [**1**·CD_2]$_0$ = 0.065 M (pink circles); [**A**] = 3.25 mM; 5.5 equivalents of K_3PO_4; the initial rates for the two parallel reactions gave a KIE of 2.0.

Inhibition by free QuinoxP ligand*

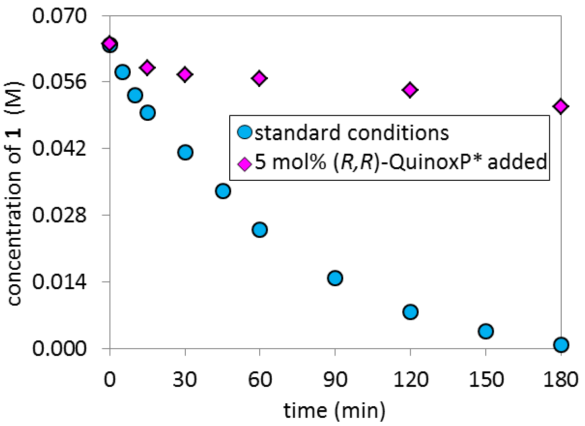

Fig. S7. Kinetic profiles monitored by ^{1}H NMR spectroscopy for the reaction of Scheme 1. (a) Standard conditions; [**1**]$_0$ = 0.065 M; [**C**]$_0$ = 3.25 mM; 5.5 equivalents of K_3PO_4; b) [**1**]$_0$ = 0.065 M; [**C**]$_0$ = 3.25 mM; [L*]$_0$ = 3.25 mM; 5.5 equivalents of K_3PO_4. Strong inhibition was observed when an additional equivalent of free (*R,R*)-QuinoxP* ligand was added.

K_3PO_4 versus K_2HPO_4

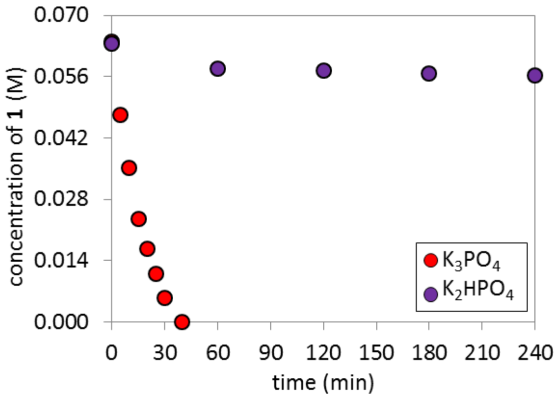

Fig. S8. Temporal concentration profiles monitored by ^{1}H NMR spectroscopy for the reaction of Scheme 1 using 5 mol% of complex **C** as the catalyst. [**1**]$_0$ as noted; [**C**]$_0$ = 3.25 mM; 10 mol% PivOH; 5.5 equivalents of K_3PO_4 or K_2HPO_4. K_3PO_4 has been proved to be a superior base for this transformation.

Tracking the ee of product and remaining sm over time

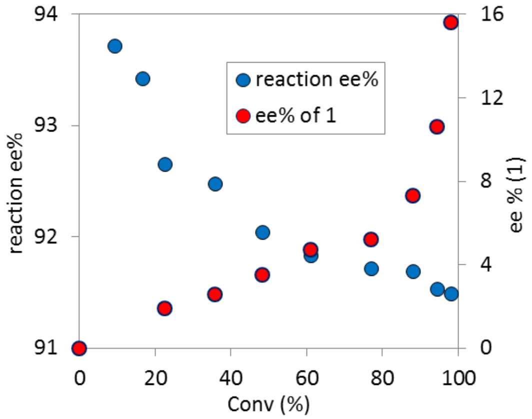

Fig. S9. Temporal reaction ee of **2** and remaining **1** for the standard reaction. [**1**]$_0$ = 0.065 M; [**A**] = 3.25 mM; 5.5 equivalents of K_3PO_4.

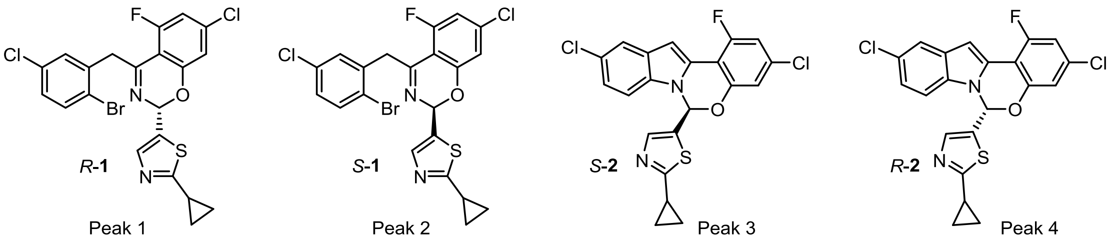

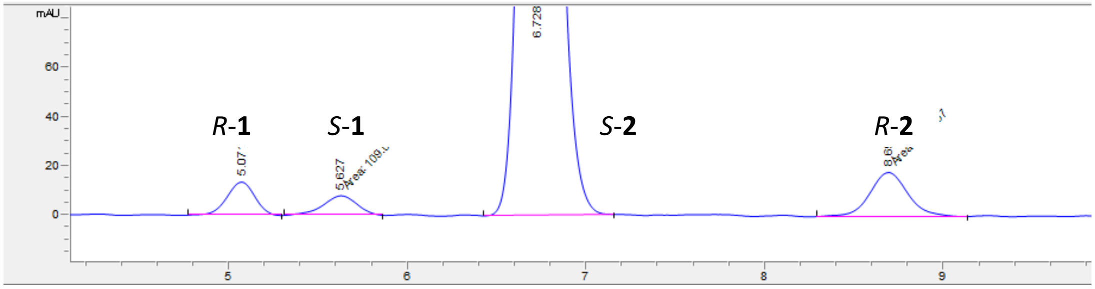

Fig. S10. Chromatograms showing the chiral separation of **1** and **2**. Method: OJ-H Column; 4.6 x 250 mm, 5 μm; MPA:MPB = CO_2:0.25% IBA (isobutanol) in IPA (isopropanol) = 80:20 for 10 min @ 220 nm.

4. Kinetic data with complex C

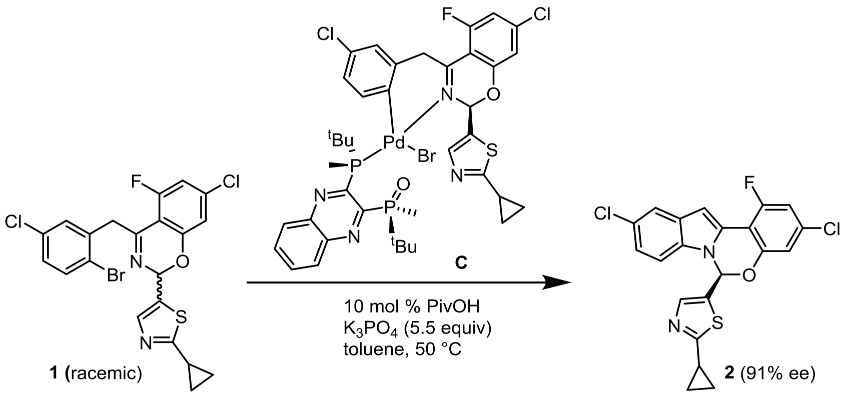

Complex C: catalyst robustness

The temporal concentration of **1** at the beginning of reaction (red hollow circles) is identical to a reaction with a higher starting concentration running for ca. 11 min (blue circles) (Fig. S11). Comparison of these two reaction profiles from a point of identical substrate concentration is possible by shifting the curve with hollow red circles in time, as shown by the red arrows to give the adjusted curve shown in red circles. The overlay between the two kinetic profiles from the time-adjusted point is a good indicator of high catalyst stability.

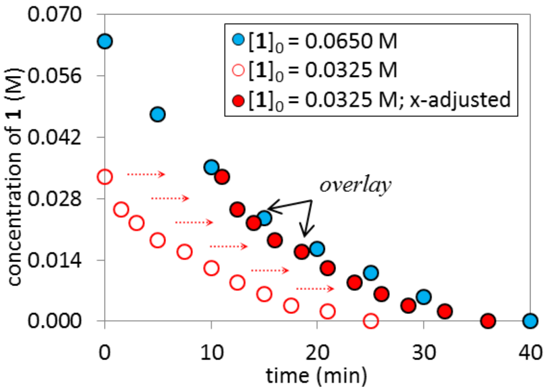

Fig. S11. Temporal concentration profiles monitored by ^{1}H NMR spectroscopy for the standard reaction using complex **C** as the catalyst. Reaction conditions: [**1**]$_0$ as noted; [**C**] = 3.25 mM; 5.5 equivalents of K_3PO_4 (based on [**1**]$_0$ = 0.065 M); 10 mol % PivOH.

First order kinetics in [C]

Having demonstrated catalyst robustness, the catalyst order was determined by using the normalized time scale method.[1] The two plots of [**1**] against a normalized time scale, $t[cat]_T^n$, for two reactions carried out at different catalyst loadings showed overlay for n = 1.

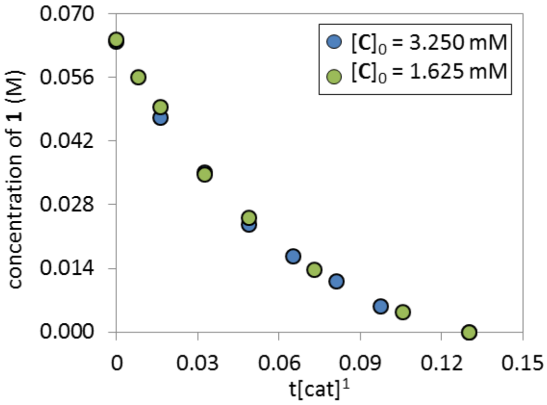

Fig. S12. Temporal concentration profiles monitored by ^{1}H NMR spectroscopy for the standard reaction. [**1**]$_0$ = 0.065 M; 5.5 equivalents of K_3PO_4; [**C**] as noted; 10 mol % PivOH.

Saturation kinetics in [1]

Using 5 mol % of complex **C**, the reaction showed saturation kinetics in [**1**], the initial rates matched up to 20% conversion for the two reactions that differed on their initial concentrations by half.

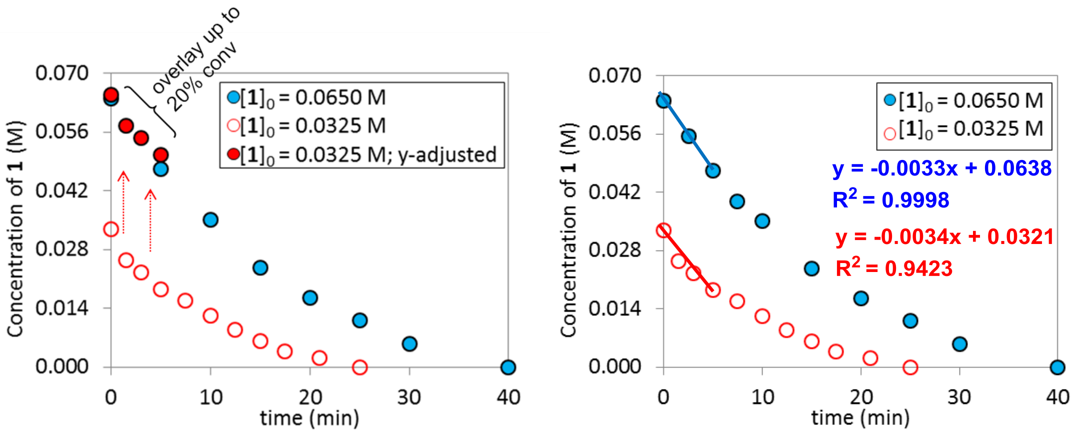

Fig. S13. Temporal concentration profiles monitored by ^{1}H NMR spectroscopy for the standard reaction. [**1**]$_0$ as noted; [**C**] = 3.25 mM; 5.5 equiv of K_3PO_4 (based on [**1**]$_0$ = 0.065 M); 10 mol % PivOH.

[1] Bures, A. J. *Angew. Chem., Int. Ed.* **2015**, *54*, 1270.

Parallel kinetic isotopic experiments

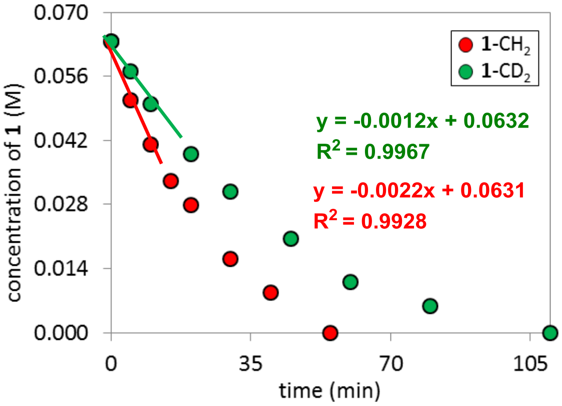

$$\mathrm{KIE} = \frac{Rate\ \mathbf{1}\cdot \mathrm{CH_2}}{Rate\ \mathbf{1}\cdot \mathrm{CD_2}} = \frac{0.0022}{0.0012} = 1.8$$

Fig. S14. Temporal concentration profiles monitored by ^{1}H NMR spectroscopy for the standard reaction carried out by using **1**·CH_2 and **1**·CD_2 in separate reactions vessels. Reaction conditions: [**1**·CH_2]$_0$ = 0.065 M (red circles) and [**1**·CD_2]$_0$ = 0.065 M (green circles); [**C**] = 3.25 mM; 5.5 equivalents of K_3PO_4; 10 mol % PivOH. The initial rates for the two parallel reactions gave a KIE of 1.8.

Tracking the ee of the remaining 1 over time

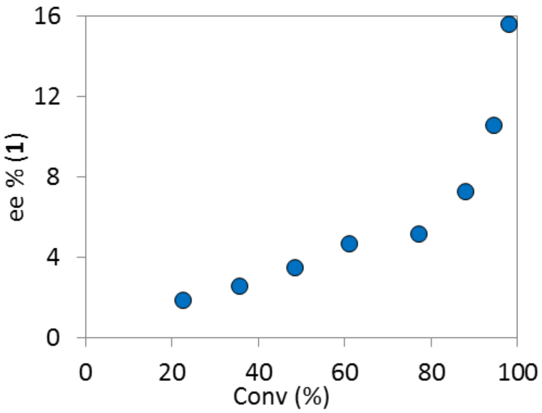

Fig. S15. Temporal ee of the remaining **1** monitored for the standard reaction. [**1**]$_0$ = 0.065 M; [**C**] = 3.25 mM; 5.5 equivalents of K_3PO_4; 10 mol % PivOH.

5. ^{1}H NMR over the reaction course for the different catalyst systems

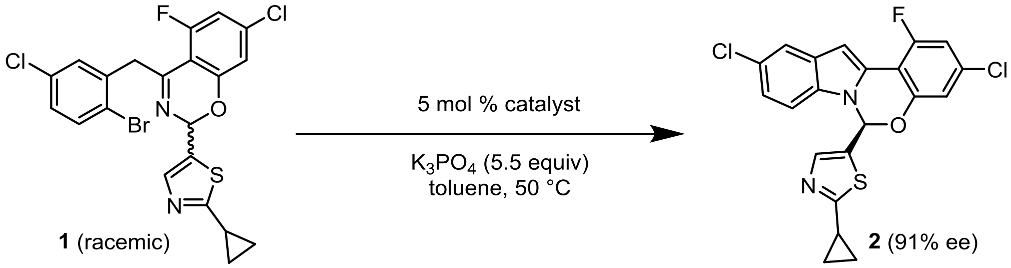

Catalyst:

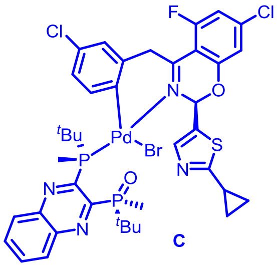

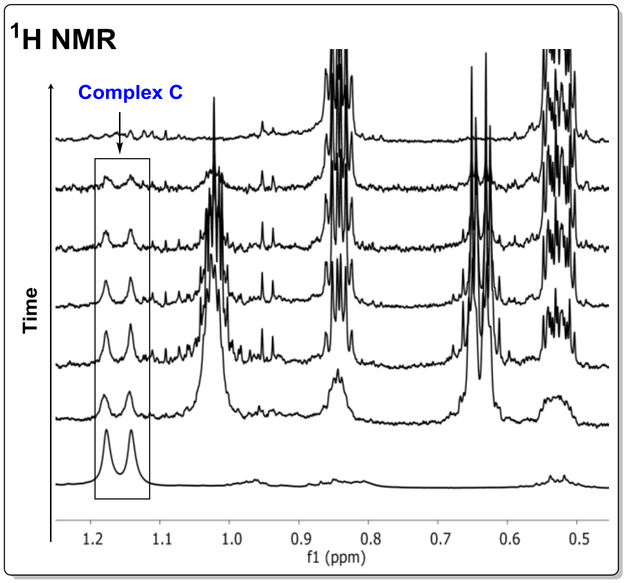

Catalyst:

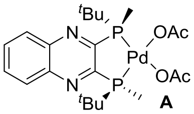

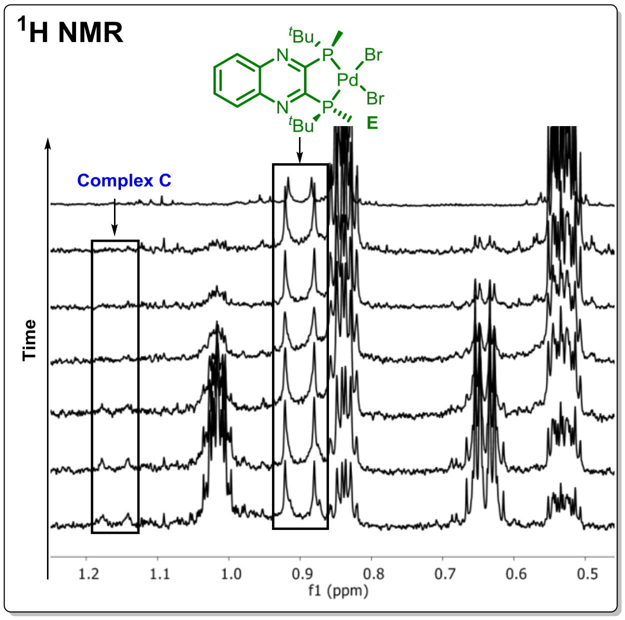

Catalyst:

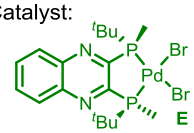

tBu
N
P
Br
Pd
Br
N
P
tBu
E

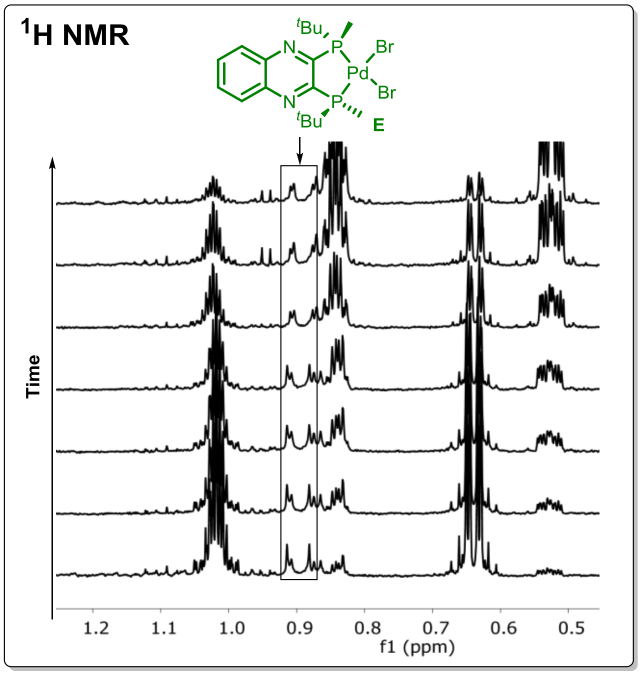

1H NMR
tBu
N
P
Br
Pd
Br
N
P
tBu
E
Time
1.2
1.1
1.0
0.9
0.8
0.7
0.6
0.5
f1 (ppm)

6. Quantification of catalytic species for the standard reaction at t = 15 min

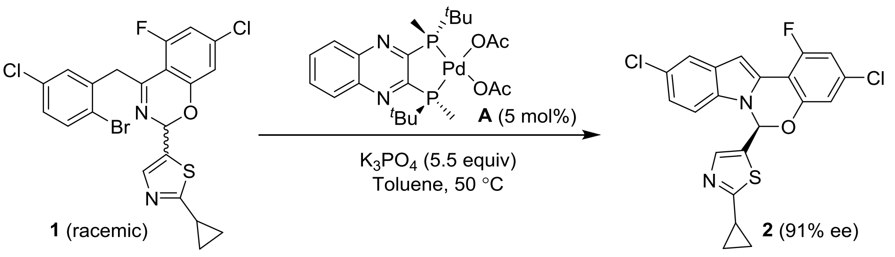

^{1}H NMR

1 (integral per proton: 2/2H = 1)
Complex **C** (integral per proton: 0.15/9H = 0.016)
QuinoxP*$PdBr_2$ (integral per proton: 0.32/9H = 0.036)

QuinoxP*$PdBr_2$: Complex **C** (~ 70:30)
(QuinoxP*$PdBr_2$ + Complex **C**) : **1** (~ 0.052:1)

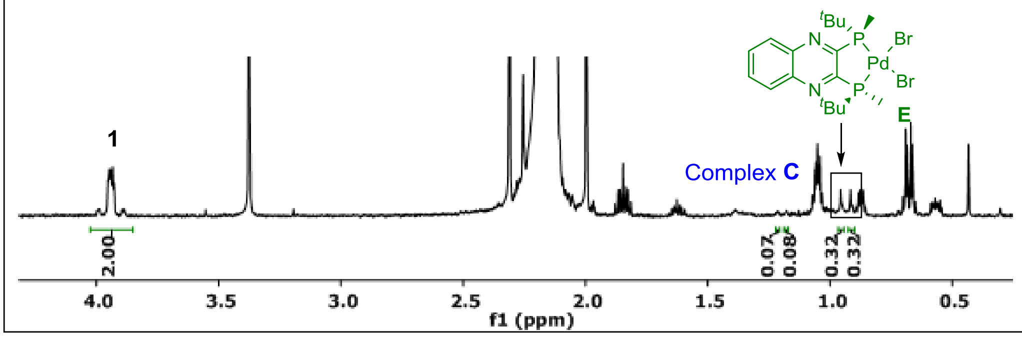

^{31}P NMR

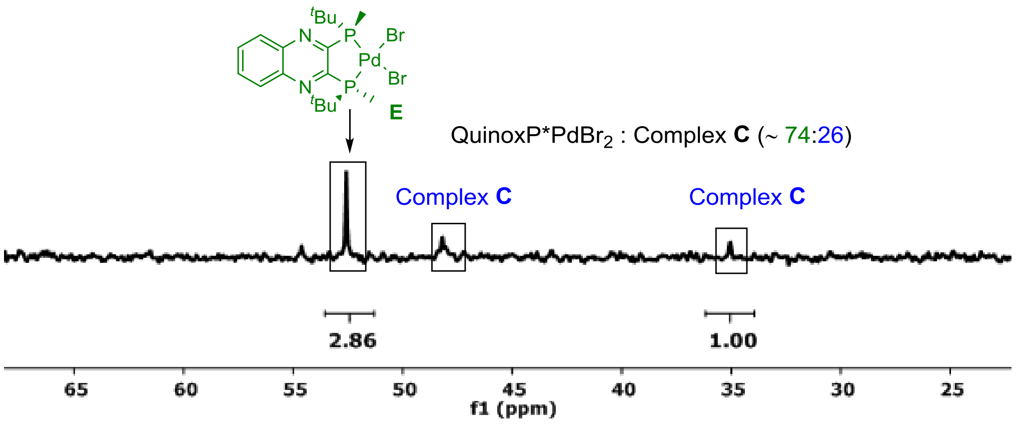

7. Stereochemical stability of the product 2

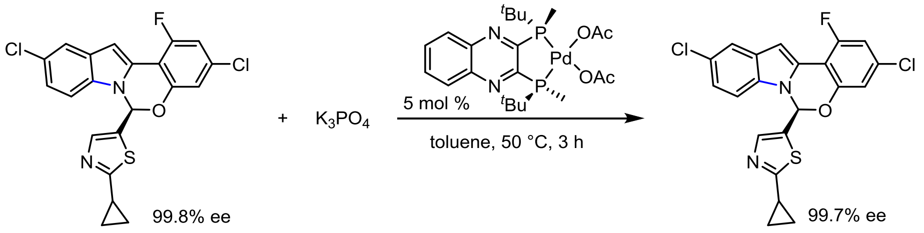

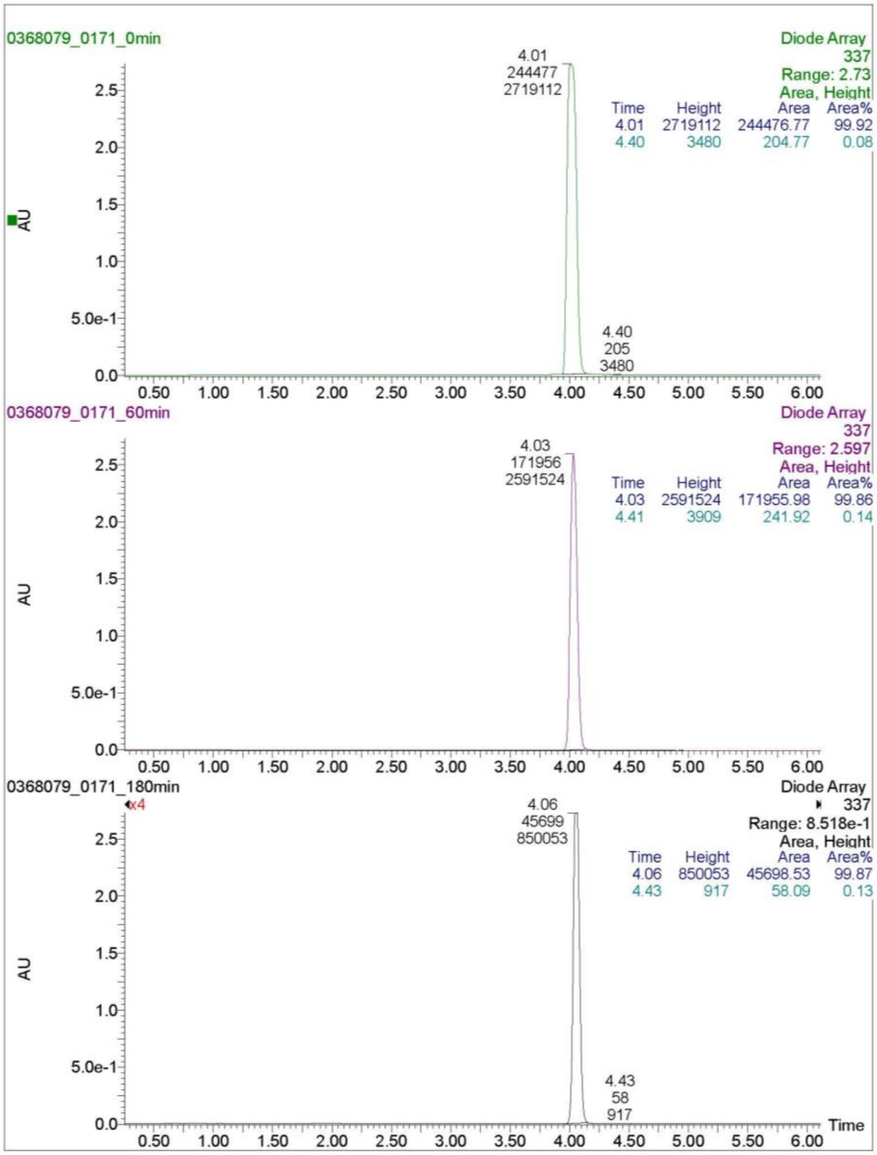

8. Synthesis of ((*R,R*)-QuinoxP*)$_2$Pd(0) F

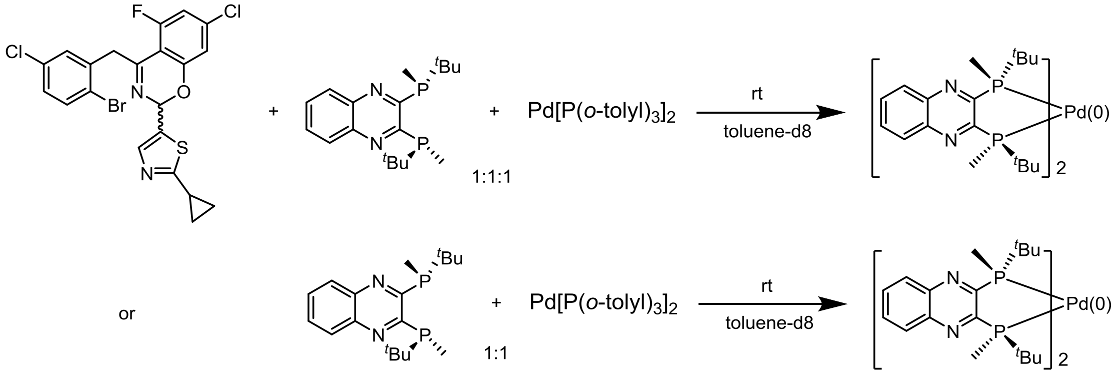

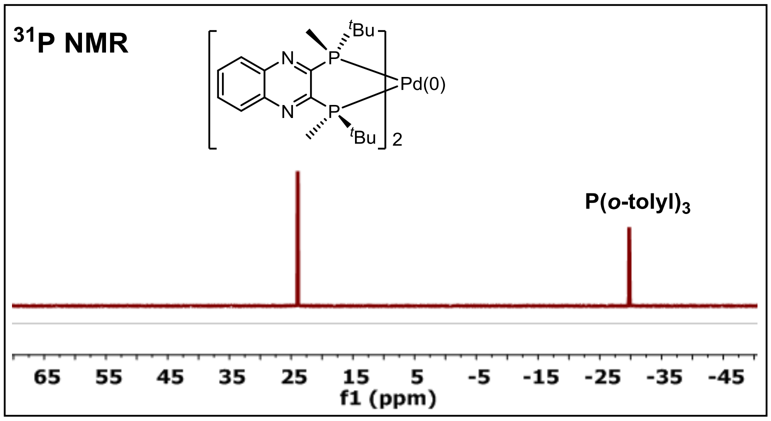

Mass spectroscopy Analysis

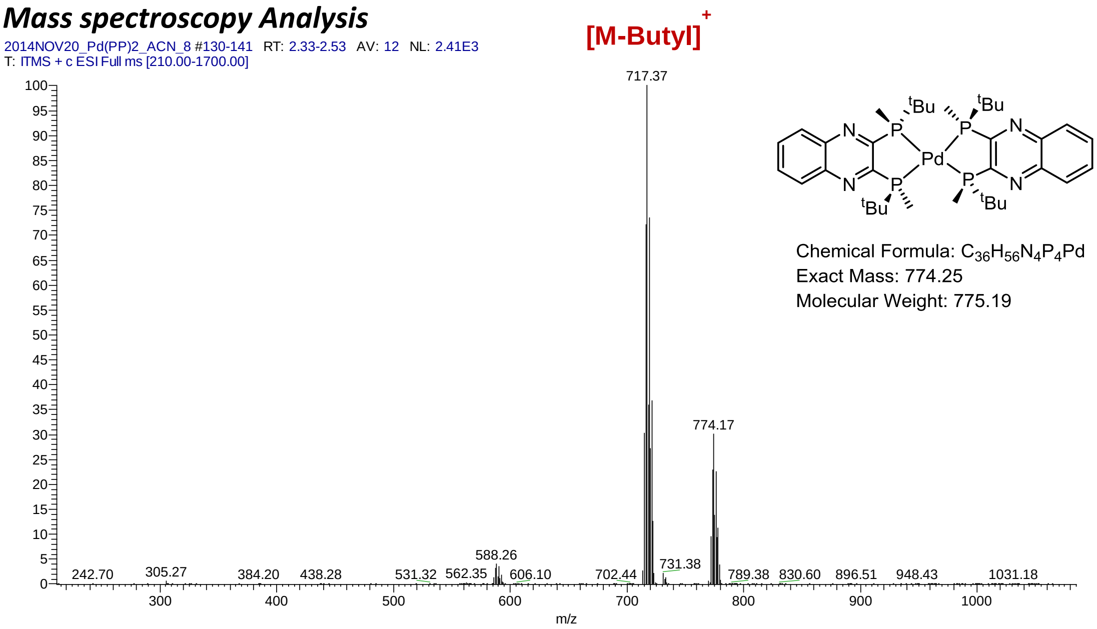

9. Screening of boronic acids using pre-catalyst K-3

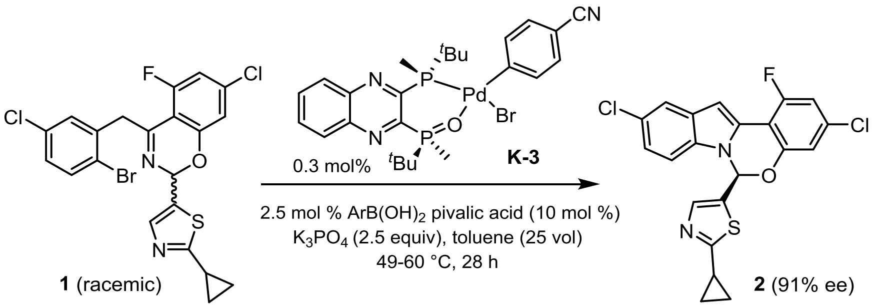

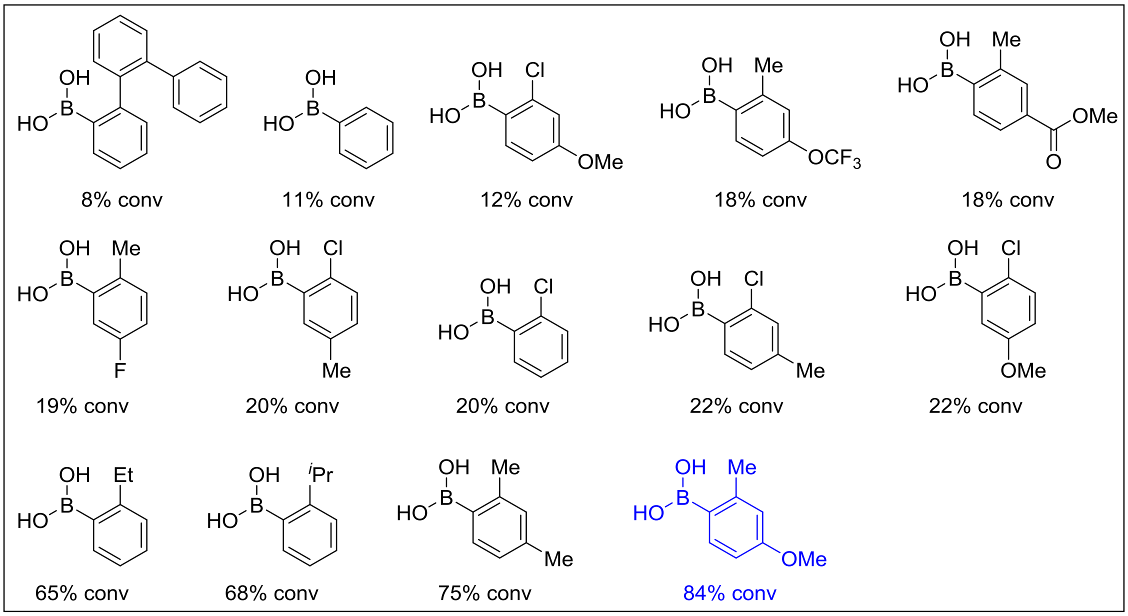

Experimental Procedure: Under a nitrogen atmosphere was added K_3PO_4 (12.7 mg, 60 μmol, 2.5 equiv) of to each of 96 reaction vials (glass vial dimensions: 0.65 mm diameter x 30 mm height) contained in a 96-well aluminum reaction block. Each vial was equipped with a 1.98 mm diameter x 4.80 mm length super tumble stir dowel. To each reaction vial was then added 24 μL (2.4 μmol) of a 0.1 M solution of pivalic acid in toluene, 30 μL (0.6 μmol) of a 0.02 M solution of the desired boronic acid in toluene, and 155 μL (24 μmol, 12.3 mg) of a 0.155 M solution of ArBr **1**. The resulting suspensions were agitated for about 5 min at ambient temperature and then 72 μL of a solution of the desired precatalyst **K-3** (0.072 μmol, 0.3 mol % Pd) was added. The reaction vials were sealed with a Teflon-lined cover and the resulting mixtures were agitated at 400 rpm on a tumble stirrer at 53 °C (external block temperature) for 19 h. After this time, a 5 μL aliquot of each reaction was diluted into 0.6 mL of isopropanol for UPLC-MS analysis.

10. Catalytic species with substrate 3

Synthesis of complex D – Crude ^{31}P NMR

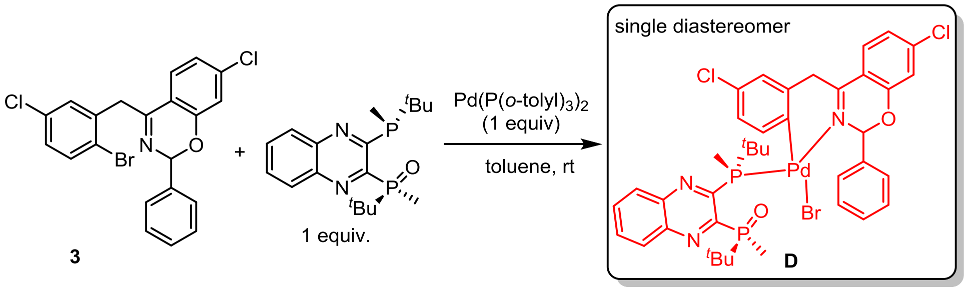

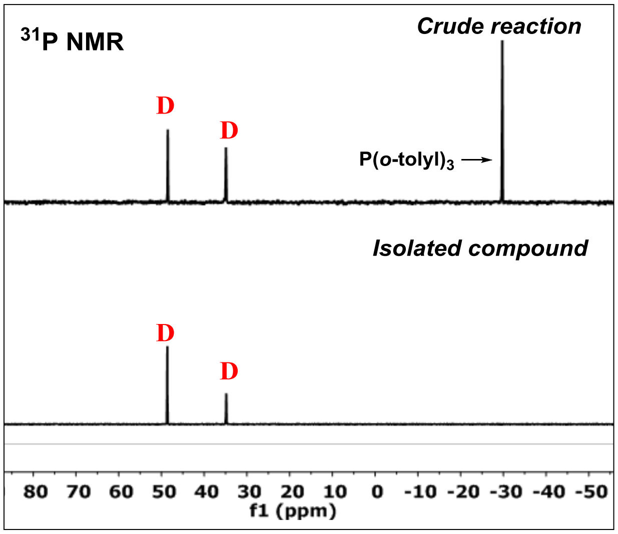

^{1}H NMR over reaction course

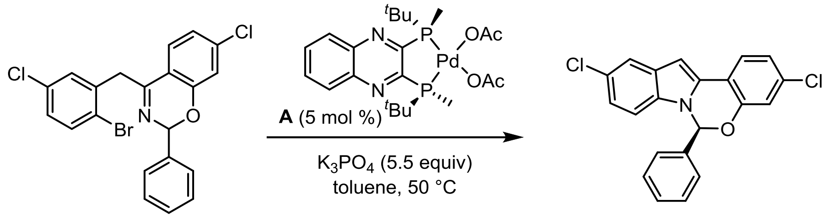

3 (racemic, 0.065 M)

Catalyst:

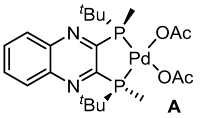

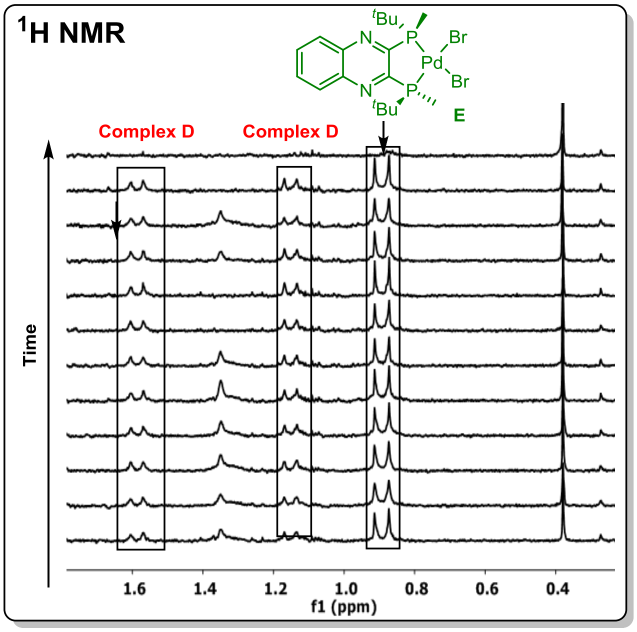

11. Buchwald G3/G4 type pre-catalyst with QuinoxP*(O)

Buchwald G3/G4 type pre-catalysts with BPMO have been prepared as shown in the scheme below:

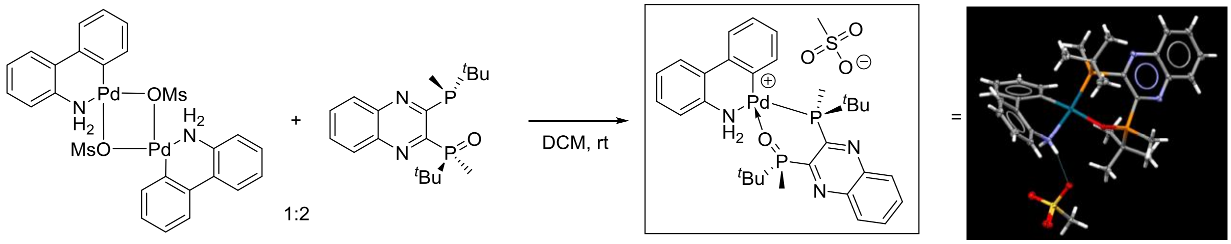

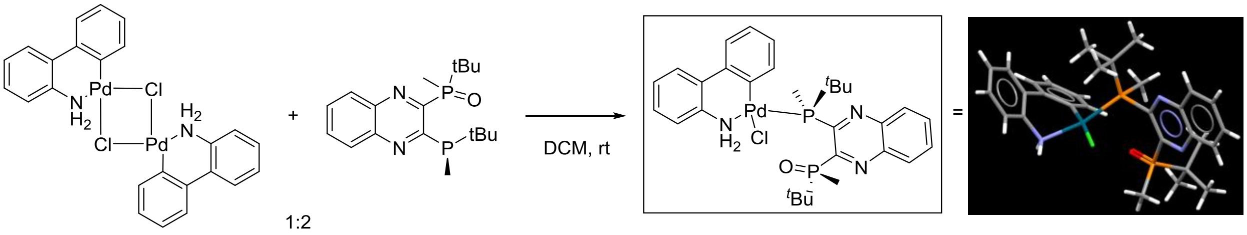

Both pre-catalysts resulted inactive in the current system, which further emphasizes the utility of catalyst type K-**3**.

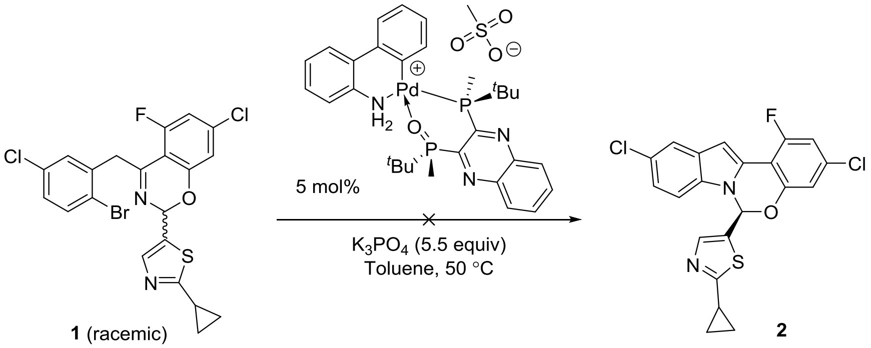

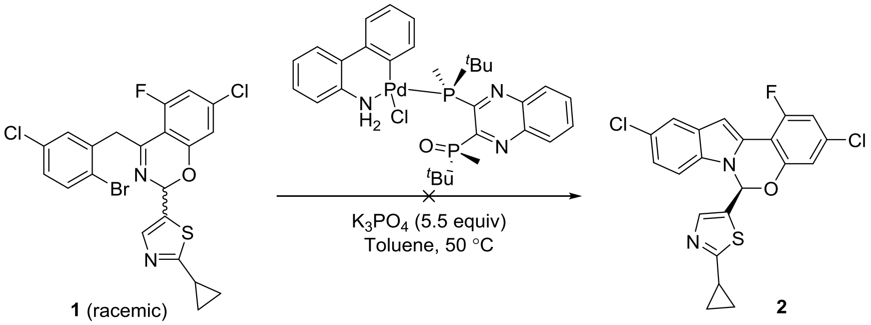

12. Experimental Procedures and Characterization

1. The synthetic route to 2

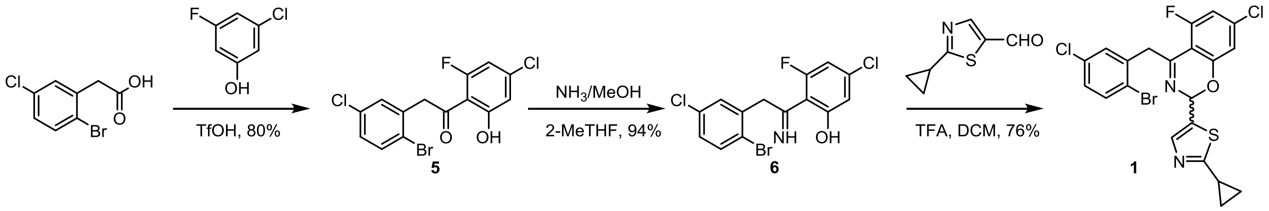

1.1 Synthesis of ketone **5**

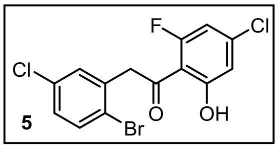

3-Chloro-5-fluorophenol (98 wt%, 10.0 g, 66.9 mmol) and 2-(2-bromo-5-chlorophenyl)acetic acid (20.02 g, 80.0 mmol) were mixed with trifluoromethanesulfonic acid (91 mL) and heated to 58 °C under a N_2 atmosphere. After 16 hours, the mixture was cooled to ambient temperature and slowly added to isopropanol (500 mL) cooled in an ice/water bath over the course of 20 min. The resulting slurry was diluted by adding water (125 mL) over 10 min. After aging for 30 min in the ice/water bath, the mixture was filtered and the solid was washed with 4:1 isopropanol/water (50 mL). The solid was dried under vacuum to provide compound **5** (20.14 g, 53.3 mmol, 80% yield) as a white solid: ^{1}H NMR (400 MHz, chloroform-*d*) δ 12.53 (s, 1H, OH), 7.54 (d, J = 8.4 Hz, 1H, CH), 7.22 (d, J = 2.5 Hz, 1H, CH), 7.19 (dd, J = 8.5, 2.6 Hz, 1H, CH), 6.86 (dd, J = 2.1, 1.4 Hz, 1H, CH), 6.72 (dd, J = 11.7, 2.1 Hz, 1H, CH), 4.43 (d, J = 4.1 Hz, 2H, CH_2) ppm. ^{13}C NMR (101 MHz, chloroform-*d*) δ 199.91 (d, J = 5.3 Hz, C=O), 164.52 (d, J = 2.5 Hz, C), 163.21 (d, J = 249.4 Hz, C), 142.65 (d, J = 15.9 Hz, C), 135.78 (d, J = 3.9 Hz, C), 133.90 (CH), 133.64 (C), 131.89 (CH), 129.37 (CH), 123.46 (C), 115.19 (d, J = 3.4 Hz, CH), 108.48 (d, J = 14.9 Hz, C), 107.93 (d, J = 28.2 Hz, CH), 50.60 (d, J = 13.7 Hz, CH_2) ppm. ^{19}F NMR (376 MHz, chloroform-*d*) δ –103.2 ppm. HRMS-ESI *m/z* calcd. for $C_{14}H_7BrCl_2FO_2^-$: 374.8991, found 374.8986 [*M*-H]$^-$.

1.2 Synthesis of imine **6** from **5**

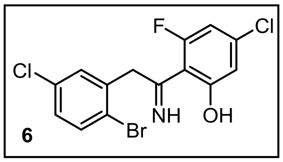

To a 3-neck round bottom flask was charged ketone **5** (5.0 g, 13.2 mmol), MeOH (25 mL), and NH_3 in MeOH (7 N, 18.9 mL, 132 mmol) to

give a clear yellow solution. After aging at room temperature for 30 min, the solution was seeded with product **6** and aged for 19 h. The mixture was then evaporated to remove ~10 mL of solvent. The slurry was filtered and the cake was washed with MeOH (10 mL). Drying gave 4.7 g product as a bright yellow solid in 94% yield (a sample was analyzed and found to contain a 97.5:2.5 ratio of **6** : **5**): ^{1}H NMR (400 MHz, chloroform-*d*) δ 7.58 – 7.62 (m, 1H, CH), 7.23 – 7.28 (m, 2H, CH), 6.87 (br, 1H, CH), 6.57 (dd, *J* = 12.6, 2.1 Hz, 1H, CH), 4.34 (d, *J* = 3.9 Hz, 2H, CH_2) ppm. ^{13}C NMR (126 MHz, chloroform-*d*) δ 175.85 (d, *J* = 4.2 Hz, C=N), 166.93 (d, *J* = 6.9 Hz, C), 162.99 (d, *J* = 255.6 Hz, C), 139.38 (d, *J* = 16.7 Hz, C), 134.90 (d, *J* = 4.3 Hz, C), 134.82 (CH), 134.50 (C), 132.51 (CH), 130.35 (CH), 123.92 (C), 115.77 (d, *J* = 3.3 Hz, CH), 106.21 (d, *J* = 11.5 Hz, C), 105.85 (d, *J* = 28.6 Hz, CH), 46.06 (d, *J* = 15.6 Hz, CH_2) ppm. ^{19}F NMR (471 MHz, chloroform-*d*) δ –105.7 ppm. HRMS-ESI *m/z* calcd. for $C_{14}H_{10}BrCl_2FNO^+$: 375.9307, found 375.9314 [*M*+H]$^+$.

1.3 Synthesis of **1** from **6**

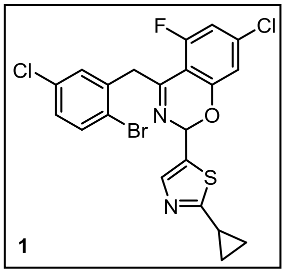

To a dry 1000 mL flask was charged activated 4 Å MS (55.0 g) followed by compound **6** (25.0 g, 66.3 mmol); 2-cyclopropylthiazole-5-carbaldehyde (20.32 g, 133 mmol) and DCM (275 mL). The slurry was cooled by ice-water and then charged with 2,2,2-trifluoroacetic acid (15.3 mL, 199 mmol) over 10 min while keeping the batch < 10 °C. The mixture was allowed to stir at room temperature for about 20 h. The reaction mixture was cooled using ice-water, and triethylamine (28.8 mL, 206 mmol) was added *via* a syringe pump (temp batch < 15 °C). The reaction mixture was then filtered to remove MS, and washed with DCM. The organic layer was washed sequentially with water (60 mL), citric acid (5% aq, 60 mL), brine (5 wt %, 100 mL), $NaHCO_3$ (2 wt %, 70 mL), brine (2 wt %, 70 mL), dried over $MgSO_4$, filtered and concentrated *in vacuo*. The resulting residue was taken up into 270 mL ethyl acetate at 45 ^{o}C, the solution was seeded and then concentrated at 200 psi to about 3-4 volumes. Heptane (200 mL) was added over 2 h and the resulting solution was cooled to 25 ^{o}C. The solid formed was filtered and washed with ethyl acetate/heptane = 1/3 (2 x 40 mL) to provide a crude product that was recrystallized from ethyl acetate/heptane (1/2) to provide

compound **1** (26.0 g, 50.8 mmol, 77%) as a light yellow solid: ^{1}H NMR (500 MHz, chloroform-*d*) δ 7.55 (s, 1H, CH), 7.52 (d, J = 8.5 Hz, 1H, CH), 7.22 (d, J = 2.5 Hz, 1H, CH), 7.14 (dd, J = 8.5, 2.5 Hz, 1H, CH), 6.82 (s, 1H, CH), 6.78 (dd, J = 10.4, 2.0 Hz, 1H, CH), 6.62 (s, 1H, CH), 4.28 (d, J = 17.4 Hz, 2H, CH_2), 4.21 (d, J = 17.4 Hz, 2H, CH_2), 2.32 – 2.24 (m, 1H, CH), 1.17 – 1.10 (m, 2H, CH_2), 1.10 – 1.04 (m, 2H, CH_2) ppm. ^{13}C NMR (126 MHz, chloroform-*d*) δ 175.42 (C), 159.98 (d, J = 6.1 Hz, C), 159.35 (d, J = 257.4 Hz, C), 156.05 (d, J = 7.1 Hz, C), 141.43 (CH), 139.72 (d, J = 14.1 Hz, C), 137.94 (d, J = 2.2 Hz, C), 133.64 (CH), 133.21 (C), 133.10 (C), 131.09 (CH), 128.55 (CH), 123.30 (C), 113.80 (d, J = 3.6 Hz, CH), 110.83 (d, J = 27.3 Hz, CH), 106.31 (d, J = 16.4 Hz, C), 83.88 (CH), 43.69 (d, J = 8.8 Hz, CH_2), 14.58 (CH), 11.28 (CH_2), 11.25 (CH_2) ppm. ^{19}F NMR (376 MHz, chloroform-*d*) δ –107.8 ppm. HRMS-ESI *m/z* calcd. for $C_{21}H_{15}BrCl_2FN_2OS^+$: 510.9449, found 510.9438 [M+H]$^+$.

1.4 Synthesis of **2** from **1**

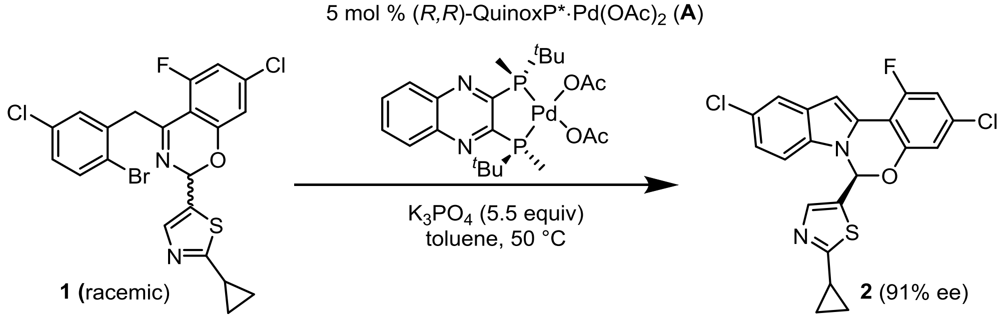

Inside of a nitrogen-filled glove box, pre-degassed anhydrous toluene (1.65 mL) was added to an 8 mL vial containing **1** (66.6 mg, 0.130 mmol) and K_3PO_4 (151.7 mg, 0.715 mmol, 5.5 equiv). The vial was placed into a preheated plate at 50 °C. The reaction was initiated by addition of 0.35 mL of a stock solution of (*R,R*)-QuinoxP*·Pd(OAc)$_2$ pre-catalyst **A** (10.4 mg in 1 mL volumetric flask). After 3 h, the mixture was cooled down to ambient temperature and filtered. The filtrate was concentrated and then purified by column chromatography using a gradient of hexanes:EtOAc to afford **2** as a white solid (53 mg, 0.122 mmol, 95% yield): $[\alpha]_D^{25}$ –9.4° (*c* 2.02, acetone, 98% ee); ^{1}H NMR (400 MHz, chloroform-*d*) δ 7.66 (dd, J = 2.0, 0.6 Hz, 1H, CH), 7.40 (d, J = 0.9 Hz, 1H, CH), 7.20 (dd, J = 8.7, 2.0 Hz, 1H, CH), 7.08 (d, J = 8.7 Hz, 2H, CH), 7.05 (d, J = 0.9

Hz, 1H, CH), 7.04 (dd, *J* = 3.5, 0.8 Hz, 1H, CH), 6.94 (dd, *J* = 9.7, 1.9 Hz, 1H, CH), 6.91 (dd, *J* = 1.9, 1.2 Hz, 1H, CH), 2.22 – 2.16 (m, 1H, CH), 1.14 – 1.06 (m, 2H, CH_2), 1.04 – 0.99 (m, 2H, CH_2) ppm. ^{13}C NMR (101 MHz, chloroform-*d*) δ 177.06 (C), 158.98 (d, *J* = 255.5 Hz, C), 149.68 (d, *J* = 7.8 Hz, C), 141.24 (CH), 134.56 (d, *J* = 12.9 Hz, C), 132.52 (C), 131.30 (C), 130.43 (C), 127.27 (C), 126.24 (d, *J* = 2.0 Hz, C), 123.98 (CH), 121.06 (CH), 114.74 (d, *J* = 3.7 Hz, CH), 111.87 (d, *J* = 24.1 Hz, CH), 110.17 (CH), 106.43 (d, *J* = 17.4 Hz, C), 102.65 (d, *J* = 9.5 Hz, CH), 79.39 (CH), 14.81 (CH), 11.79 (CH_2), 11.74 (CH_2) ppm. ^{19}F NMR (376 MHz, chloroform-*d*) δ –112.4 ppm. HRMS-ESI *m/z* calcd. for $C_{21}H_{14}Cl_2FN_2OS^+$: 431.0188, found 431.0191 $[M+H]^+$.

2. Synthesis of 1·CD₂ from 1

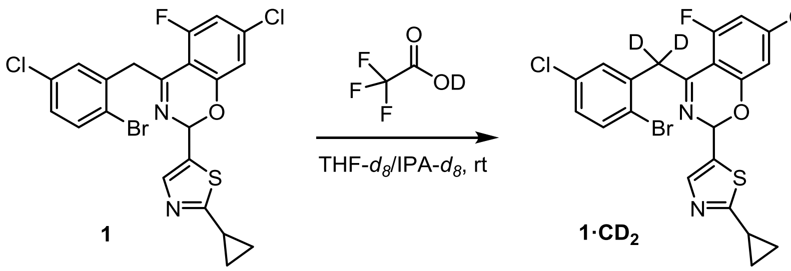

To a solution of **1** (1.0 g, 1.95 mmol) in 10 mL of THF-d_8 and 3.0 mL of IPA-d_8 was added 0.4 mL of TFA-*d*. The solution was left to stir at room temperature for 3 h. The solvent was then removed under vacuum. The oil obtained was dried under vacuum for 1 h and diluted with 6 mL of D_2O, 4 mL of IPA-d_8 and 600 μL of DCl (37% in D_2O) and stirred for 5 min. The solution was extracted with 40 mL of CH_2Cl_2. The organic layer was dried over Na_2SO_4, treated with 2.0 g of Na_2CO_3, and stirred for 5 min. The solids were filtered and the organic solvent was concentrated. CH_3CN (10 mL) was added to the oil and then evaporated. The resulted solid was then suspended in CH_3CN (10 mL) and stirred at ambient temperature for 10 min. The solvent was removed under reduced pressure until ~8 ml was left over and the resulting solids were filtered and washed with CH_3CN (2 x 1 mL), then dried under vacuum to afford the desired product **1·CD₂** (700 mg, 1.37 mmol, 70% yield, 97% deuterium incorporation) as a white solid: ^1H NMR (500 MHz, DMSO-d_6) δ 7.64 (d, *J* = 8.5 Hz, 1H, CH), 7.56 (d, *J* = 0.9 Hz, 1H, CH), 7.51 (d, *J* = 2.6 Hz, 1H, CH), 7.29 (dd, *J* = 8.5, 2.7 Hz, 1H, CH), 7.26 (dd, *J* = 10.7, 2.0 Hz, 1H, CH), 7.09 (dd, *J*

= 2.0, 1.0 Hz, 1H, CH), 6.87 (d, J = 0.9 Hz, 1H), 2.39 – 2.33 (m, 1H), 1.11 – 1.06 (m, 1H), 0.94 – 0.91 (m, 1H) ppm. ^{13}C NMR (126 MHz, DMSO-d_6) δ 174.80 (C), 159.93 (d, J = 6.0 Hz, C), 159.42 (d, J = 255.8 Hz, C), 156.03 (d, J = 7.4 Hz, C), 142.10 (CH), 139.08 (d, J = 14.6 Hz, C), 138.95 (d, J = 2.4 Hz, C), 134.14 (CH), 133.44 (C), 132.47 (CH), 132.10 (CH), 129.00 (CH), 123.81 (C), 114.10 (d, J = 3.4 Hz, CH), 111.26 (d, J = 27.4 Hz, CH), 106.74 (d, J = 16.7 Hz, C), 83.40 (CH), 40.72 (quin, J = 9.6 Hz, CD_2), 14.50 (CH), 11.52 (CH_2), 11.49 (CH_2) ppm. ^{19}F NMR (471 MHz, DMSO-d_6) δ –107.2 ppm. HRMS-ESI m/z calcd. for $C_{21}H_{13}D_2BrCl_2FN_2OS^+$: 512.9575, found 512.9597 $[M+H]^+$.

3. Synthesis of 4 from 1

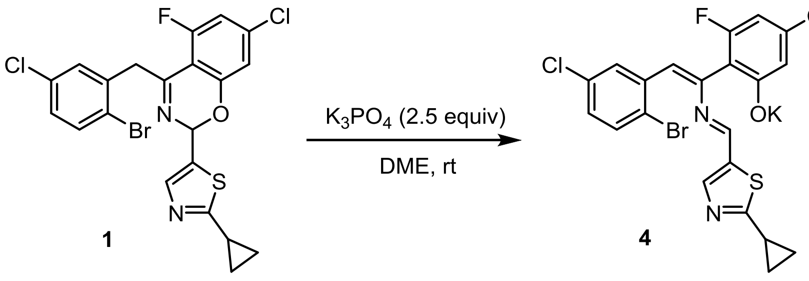

A mixture of **1** (1.70 g, 3.32 mmol) and K_3PO_4 (1.76 g, 8.3 mmol, 2.5 equiv) in 20 mL of DME was left to stir at room temperature overnight. The slurry was then filtered, the solids rinsed with DME, and the solution concentrated under vacuum. The obtained solid was recrystallized from THF/heptane (1:2, 30 mL). The yellow solids were filtered and dried with a stream of nitrogen (1.1 g, 60% yield): ^{1}H NMR (500 MHz, DMSO-d_6) δ 8.45 (d, J = 2.6 Hz, 1H), 8.19 (s, 1H, CH), 8.06 (s, 1H, CH), 7.65 (d, J = 8.5 Hz, 1H, CH), 7.25 (dd, J = 8.6, 2.7 Hz, 1H, CH), 6.36 (s, 1H, CH), 6.30 (d, J = 13.6 Hz, 1H, CH), 6.25 – 6.14 (br, 1H, CH), 2.50 – 2.42 (m, 1H, CH), 1.22 – 1.18 (m, 2H, CH_2), 1.08 – 1.01 (m, 2H, CH_2) ppm. ^{13}C NMR (126 MHz, DMSO-d_6) δ 177.59 (d, J = 3.5 Hz, C), 166.41 (C), 160.82 (d, J = 245.6 Hz, C), 151.26 (CH), 147.86 (CH), 144.07 (d, J = 21.1 Hz, C), 137.08 (C), 136.78 (d, J = 2.7 Hz, C), 133.66 (CH), 133.38 (d, J = 17.0 Hz, C), 132.71 (CH), 131.75 (C), 127.96 (CH), 123.96 (d, J = 8.8 Hz, CH), 121.93 (C), 114.02 (d, J = 10.6 Hz, CH), 111.34 (d, J = 13.5 Hz, C), 98.52 (CH), 14.84 (CH), 12.00 (2 CH_2) ppm. ^{19}F NMR (471 MHz, DMSO-d_6) δ –113.7 ppm. HRMS-ESI m/z calcd. for $C_{21}H_{15}BrCl_2FN_2OS^+$: 510.9450, found 510.9457 $[M\text{-}K+2H]^+$.

4. Synthesis of pre-catalysts

4.1 Synthesis of (*R,R*)-QuinoxP*Pd(OAc)$_2$, **A**

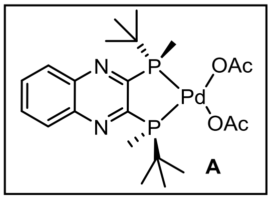

In an 8 mL vial, a mixture of Pd(OAc)$_2$ (300 mg, 1.34 mmol) and (*R,R*)-QuinoxP* (448 mg, 1.34 mmol) was dissolved in DCM (1.8 mL). The mixture was left to stir for 10 min. Heptane (6 mL) was slowly added causing solids to crash out from the solution. The reaction was stirred for 1 h at ambient temperature. The solids were then filtered and rinsed with *n*-hexane. Solids of light olive color were obtained (0.63 g, 1.12 mmol, 83 % yield): ^{1}H NMR (400 MHz, toluene-*d*$_8$) δ 7.81 (dd, *J* = 6.5, 3.5 Hz, 2H, CH), 7.19 (dd, *J* = 6.5, 3.4 Hz, 2H, CH), 2.20 (s, 6H, CH$_3$), 1.94 (d, *J* = 12.0 Hz, 6H, CH$_3$), 1.11 (d, *J* = 16.1 Hz, 18H, CH$_3$) ppm. ^{13}C NMR (151 MHz, toluene-*d*$_8$) δ 176.46 (C=O), 156.58 (t, *J* = 54.9 Hz, C), 142.70 (t, *J* = 3.9 Hz, C), 132.67 (CH), 130.10 (CH), 36.88 – 35.47 (m, C), 26.85 (CH$_3$), 24.23 (CH$_3$), 7.14 – 5.57 (m, CH$_3$) ppm. ^{31}P NMR (202.5 MHz, toluene-*d*$_8$) δ 52.3 ppm. HRMS-ESI *m/z* calcd. for $C_{20}H_{31}N_2O_2P_2Pd^+$: 499.0890, found 499.0881 [*M*-OAc]$^+$.

4.2 Synthesis of (*R,R*)-QuinoxP*PdBr$_2$, **E**

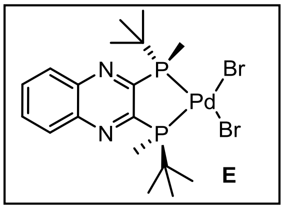

To a solution of (*R,R*)-QuinoxP* (168 mg, 0.502 mmol) and ArBr **1** (1.03 g, 2.01 mmol) in toluene (35 mL) was added Pd(OAc)$_2$ (113 mg, 0.502 mmol). The reaction mixture was heated at 50 °C overnight. A precipitate that had formed was filtered under and washed with toluene to afford a pale yellow solid (270 mg, 89 % yield): ^{1}H NMR (500 MHz, chloroform-*d*) δ 8.35 (dd, *J* = 6.5, 3.5 Hz, 2H), 8.07 (dd, *J* = 6.5, 3.4 Hz, 2H), 2.38 (d, *J* = 11.7 Hz, 6H), 1.24 (d, *J* = 16.5 Hz, 18H) ppm. ^{13}C NMR (126 MHz, chloroform-*d*) δ 154.51 (t, *J* = 56.0 Hz, C), 142.72 (t, *J* = 3.9 Hz, C), 133.48 (CH), 130.34 (CH), 37.70 – 35.43 (m, C), 28.50 (t, *J* = 2.1 Hz, CH$_3$), 8.01 – 7.49 (m, CH$_3$) ppm. ^{31}P NMR (202.5 MHz, chloroform-*d*) δ 54.9 ppm. HRMS-ESI *m/z* calcd. for $C_{18}H_{28}BrN_2P_2Pd^+$: 518.9940, found 518.9950 [*M*-Br]$^+$.

5. Synthesis of oxidative addition complexes

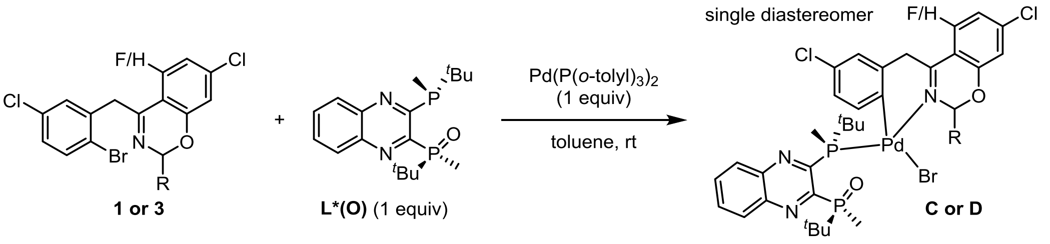

5.1 General procedure for synthesis of complexes **C/D**

A mixture of ArBr **1** (50 mg, 0.098 mmol) or **3** (44 mg, 0.098 mmol), (*R,R*)-QuinoxP*(O) (34.2 mg, 0.098 mmol) and Pd[P(*o*-tolyl)$_3$]$_2$ (70 mg, 0.098 mmol) was placed in an 8 mL vial inside of the glove box. 2 mL of toluene was added to the mixture. An initial dark green slurry was observed, which becomes a dark yellow-brown solution after stirring for an hour. The solvent was removed under vacuum. To the thick red-brown oil was added TBME and *n*-hexane (1:1) causing a dark yellow solid to crash out. The solid was filtered and washed (3x) with a mixture of TBME and *n*-hexane (1:1). The solid was further purified by column chromatography using a gradient of hexanes:EtOAc to afford a yellow solid (34 mg, 40% yield) for **C** and (65 mg, 72% yield) for **D**.

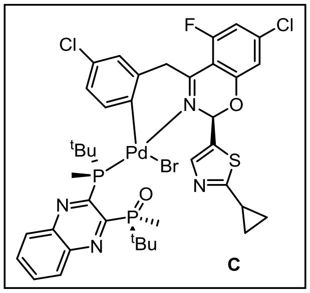

^{1}H NMR (500 MHz, toluene-d_8) δ 8.55 (br, 1H, CH), 8.47 (s, 1H, CH), 7.77 (d, J = 7.8 Hz, 1H, CH), 7.73 (d, J = 8.0 Hz, 1H, CH), 7.6 (s, 1H, CH), 6.95 – 7.20 (m, 4H, CH), 6.4 (s, 1H, CH), 6.05 (dd, J = 11.0, 2.0 Hz, 1H, CH), 4.16 (d, J = 13.7 Hz, 1H, CH$_2$), 4.05 (d, J = 14.4 Hz, 1H, CH$_2$), 2.00 (d, J = 13.1 Hz, 3H, CH$_3$), 1.95 (d, J = 10.8 Hz, 3H, CH$_3$), 1.67-1.74 (m, 1H, CH), 1.57 (d, J = 14.6 Hz, 9H, CH$_3$), 1.14 (d, J = 14.2 Hz, 9H, CH$_3$), 0.91 – 0.98 (m, 1H, CH$_2$), 0.75 – 0.87 (m, 1H, CH$_2$), 0.43 – 0.56 (m, 2H, CH$_2$) ppm. ^{13}C NMR (126 MHz, toluene-d_8) δ 174.42 (C), 165.89 (dd, J = 5.7, 2.9 Hz, C), 161.14 (dd, J = 110.2, 19.2 Hz, C), 159.74 (d, J = 259.8 Hz, C), 157.20 (dd, J = 47.8, 20.7 Hz, C), 155.06 (d, J = 5.6 Hz, C), 152.24 (d, J = 2.0 Hz, CH), 143.17 (CH), 141.30 (d, J = 14.4 Hz, C), 139.42 (d, J = 9.0 Hz, CH), 139.34 (d, J = 16.6 Hz, C), 138.81 (C), 138.69 (d, J = 2.4 Hz, C), 138.61 (d, J = 2.4 Hz, C), 131.35 (CH), 130.84 (CH), 130.64 (C), 130.36 (C), 129.46 (CH), 129.27 (CH), 128.45 (CH), 114.29 (d, J = 3.5 Hz, CH), 111.24 (d, J = 33.2 Hz, CH),

106.40 (dd, J = 13.1, 3.2 Hz, C), 85.19 (d, J = 1.8 Hz, CH), 47.47 (d, J = 10.5 Hz, CH_2), 35.90 (d, J = 25.9 Hz, C), 34.94 (d, J = 68.5 Hz, C), 28.92 (d, J = 4.8 Hz, CH_3), 29.59 (CH_3), 14.63 (CH), 12.87 (d, J = 66.4 Hz, CH_3), 11.26 (CH_2), 11.13 (CH_2), 7.82 (d, J = 28.0 Hz, CH_3) ppm. ^{19}F NMR (376 MHz, toluene-d_8) δ –105.7 ppm; ^{31}P NMR (202 MHz, toluene-d_8) δ 48.6 (P=O) and 35.2 (P) ppm. HRMS-ESI m/z calcd. for $C_{39}H_{42}Cl_2FN_4O_2P_2PdS^+$: 885.0905, found 885.0916 [M-Br]$^+$.

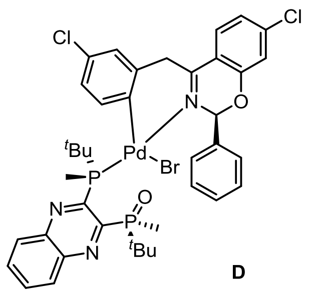

D

^{1}H NMR (500 MHz, toluene-d_8) δ 8.60 (dd, J = 8.4, 3.2 Hz, 1H, CH), 8.38 (s, 1H, CH), 7.81 – 7.76 (m, 1H, CH), 7.74 – 7.71 (m, 1H, CH), 7.66 (d, J = 7.7 Hz, 2H, CH), 7.25 – 7.16 (m, 2H, CH), 6.88 (t, J = 7.4 Hz, 1H, CH), 6.68 (d, J = 2.0 Hz, 1H, CH), 6.60 (d, J = 8.5 Hz, 1H, CH), 6.43 (dd, J = 8.5, 2.1 Hz, 1H, CH), 3.90 (d, J = 14.5 Hz, 1H, CH_2), 3.61 (d, J = 14.5 Hz, 1H, CH_2), 1.96 (d, J = 10.7 Hz, 3H, CH_3), 1.88 (d, J = 13.2 Hz, 3H, CH_3), 1.59 (d, J = 14.6 Hz, 9H, CH_3), 1.15 (d, J = 14.2 Hz, 9H, CH_3) ppm; 4 CH signals are missing due to solvent overlap. ^{13}C NMR (126 MHz, toluene-d_8) δ 167.01 (d, J = 3.0 Hz, C), 161.36 (dd, J = 47.2, 20.5 Hz, C), 157.25 (dd, J = 109.4, 19.3 Hz, C), 154.87 (C), 152.98 (d, J = 2.7 Hz, C), 140.88 (C), 139.30 (CH), 139.21 (d, J = 8.8 Hz, C), 138.66 (dd, J = 9.7, 2.5 Hz, C), 138.61 (C), 131.31 (CH), 130.80 (CH), 130.00 (C), 129.44 (CH), 127.42 (2CH), 126.72 (CH), 125.60 (CH), 122.22 (CH), 117.83 (CH), 116.29 (d, J = 3.2 Hz, C), 88.73 (CH), 45.01 (CH_2), 35.78 (d, J = 25.8 Hz, C), 34.91 (d, J = 68.6 Hz, C), 28.89 (d, J = 4.3 Hz, CH_3), 25.52 (CH_3), 12.77 (d, J = 66.3 Hz, CH_3), 7.88 (d, J = 27.4 Hz, CH_3) ppm; 1 C and 5 CH signals are missing due to solvent overlap. ^{31}P NMR (202 MHz, toluene-d_8) δ 48.6 (P=O) and 35.2 (P) ppm. HRMS-ESI m/z calcd. for $C_{39}H_{42}Cl_2N_3O_2P_2Pd^+$: 820.1169, found 820.1163 [M-Br]$^+$.

5.2 General procedure for synthesis of complexes **K**

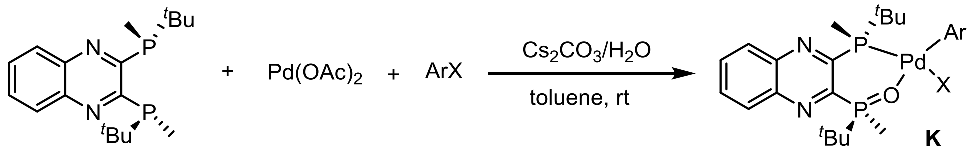

Inside of the glove box, a solution of $Pd(OAc)_2$ (134 mg, 0.598 mmol), (*R,R*)-QuinoxP* (200 mg, 0.598 mmol) and ArX (1.495 mmol) in degassed toluene (10 mL) was stirred for 1 h at room temperature. To the solution was then charged Cs_2CO_3 (974 mg, 2.99 mmol) and water (0.054 g, 2.99 mmol). The mixture was stirred at ambient temperature overnight. The mixture was then filtered and concentrated to dryness.

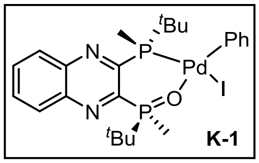

The residue was purified by flash chromatography (ethyl acetate/hexanes: 2/1) to give a light brown solid (0.32 g, 81% yield): ^{1}H NMR (500 MHz, chloroform-*d*) δ 8.22 – 8.14 (m, 2H, CH), 8.00 – 7.94 (m, 2H, CH), 7.39 – 7.36 (m, 1H, CH), 7.14 – 7.12 (m, 1H, CH), 6.93 (t, *J* = 7.5 Hz, 1H, CH), 6.88 (t, *J* = 7.5 Hz, 1H, CH), 6.76 (tt, *J* = 7.2, 1.2 Hz, 1H, CH), 1.99 (d, *J* = 12.9 Hz, 3H, CH_3), 1.60 (d, *J* = 16.0 Hz, 9H, CH_3), 1.26 (d, *J* = 15.2 Hz, 9H, CH_3), 1.16 (d, *J* = 8.5 Hz, 3H, CH_3) ppm. ^{13}C NMR (126 MHz, chloroform-*d*) δ 154.10 (dd, *J* = 103.6, 23.3 Hz, C), 152.93 (dd, *J* = 28.7, 18.7 Hz, C), 141.35 (dd, *J* = 7.0, 2.4 Hz, C), 140.19 (C), 140.00 (dd, *J* = 15.8, 1.7 Hz, C), 138.03 (d, *J* = 3.3 Hz, CH), 136.60 (d, *J* = 5.3 Hz, CH), 133.14 (CH), 132.75 (CH), 129.87 (CH), 129.48 (CH), 127.15 (d, *J* = 2.3 Hz, CH), 126.69 (CH), 122.22 (CH), 36.08 (d, *J* = 21.6 Hz, C), 34.73 (d, *J* = 64.6 Hz, C), 27.60 (d, *J* = 5.2 Hz, CH_3), 25.38 (CH_3), 10.85 (d, *J* = 71.7 Hz, CH_3), 5.05 (d, *J* = 31.5 Hz, CH_3) ppm. ^{31}P NMR (202 MHz, chloroform-*d*) δ 62.5 (d, *J* = 3.8 Hz), 22.5 (d, *J* = 3.7 Hz) ppm. HRMS-ESI *m/z* calcd. for $C_{24}H_{33}N_2OP_2Pd^+$: 531.1109, found 531.1105 $[M\text{-I}]^+$.

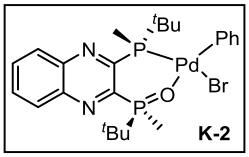

The residue was purified by flash chromatography (ethyl acetate/hexanes: 2/1) to give light yellow solid (0.25 g, 68% yield): ^{1}H NMR (500 MHz, chloroform-*d*) δ 8.24 – 8.13 (m, 2H, CH), 8.01 – 7.93 (m, 2H, CH), 7.38 (d, *J* = 7.7 Hz, 1H, CH), 7.16 (d, *J* = 5.9 Hz, 1H, CH), 6.94 (t, *J* = 7.6 Hz, 1H, CH), 6.90 (t, *J* = 7.5 Hz, 1H,

CH), 6.80 (tt, *J* = 7.2, 1.2 Hz, 1H, CH), 1.98 (d, *J* = 12.9 Hz, 3H, CH_3), 1.60 (d, *J* = 16.0 Hz, 9H, CH_3), 1.26 (d, *J* = 15.4 Hz, 9H, CH_3), 1.24 (d, *J* = 8.7 Hz, 3H, CH_3) ppm. ^{13}C NMR (126 MHz, chloroform-*d*) *δ* 154.19 (dd, *J* = 104.1, 23.3 Hz, C), 152.87 (dd, *J* = 32.7, 18.9 Hz, C), 143.22 (C), 141.28 (dd, *J* = 7.4, 2.4 Hz, C), 140.00 (dd, *J* = 15.7, 1.7 Hz, C), 136.73 (CH), 136.11 (d, *J* = 4.8 Hz, CH), 133.17 (CH), 132.79 (CH), 129.89 (CH), 129.49 (CH), 127.43 (CH), 126.96 (CH), 122.49 (CH), 35.88 (d, *J* = 23.6 Hz, C), 34.65 (d, *J* = 64.5 Hz, C), 27.55 (d, *J* = 5.0 Hz, CH_3), 25.33 (CH_3), 10.86 (d, *J* = 71.7 Hz, CH_3), 5.99 (d, *J* = 33.3 Hz, CH_3) ppm. ^{31}P NMR (202 MHz, chloroform-*d*) *δ* 62.5 (d, *J* = 4.2 Hz), 26.8 (d, *J* = 4.2 Hz) ppm. HRMS-ESI *m/z* calcd. for $C_{24}H_{33}N_2OP_2Pd^+$: 531.1109, found 531.1105 $[M\text{-Br}]^+$.

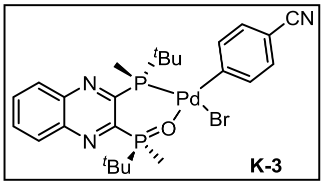

A 1 liter 3-neck flask under nitrogen was charged with 4-bromobenzonitrile (3.40 g, 18.69 mmol), $Pd(OAc)_2$ (3.36 g, 14.95 mmol), (*R,R*)-QuinoxP* (5.0 g, 14.95 mmol), and degassed toluene (500 mL, water content ~5 ppm). The mixture was then stirred at room temperature for 1 h to afford a dark brown solution (solution A). To another 1 liter 3-neck flask equipped with overhead stirrer was charged K_3PO_4 (31.7 g, 150 mmol) and toluene (100 mL). The reaction flask was degassed by vacuum/nitrogen refill cycles (3x) followed by addition of H_2O (1.35 mL, 5 equiv). Then solution A was transferred to the suspension *via* cannula. At this point another 5 equiv of H_2O and 9 g of K_3PO_4 was added into the suspension, which was left to stir overnight at ambient temperature. The reaction mixture was then filtered and the inorganic salt was washed with toluene (2 x 50 mL). The filtrate was concentrated to ~400 mL, and charged with seed. The resulting suspension was stirred at room temperature for 4 h. The solid was filtered and washed with toluene and dried at under vacuum with N_2 flow to give 7.40 g of **K-3** as a light yellow solid. The same crystallization protocol was applied to the filtrate to recover the second crop (0.84 g), providing a combined yield of 86%: ^{1}H NMR (500 MHz, chloroform-*d*) *δ* 8.25 – 8.13 (m, 2H, CH), 8.04 – 7.95 (m, 2H, CH), 7.58 (d, *J* = 8.0 Hz, 1H, CH), 7.36 (d, *J* = 7.5 Hz, 1H, CH), 7.18 (d, *J* = 8.0 Hz, 1H, CH), 7.14 (d, *J* = 7.9 Hz, 1H, CH), 1.99 (d, *J* = 12.8 Hz, 3H, CH_3), 1.58 (d, *J* = 16.0 Hz, 9H, CH_3), 1.25 (d, *J* = 15.7 Hz, 9H, CH_3). 1.24 (d, *J* = 8.5 Hz, 3H, CH_3) ppm. ^{13}C NMR (126 MHz, chloroform-*d*) *δ* 154.73 (C), 153.66 (dd, *J* = 104.7, 23.3 Hz, C),

151.59 (dd, J = 35.9, 18.7 Hz, C), 141.26 (dd, J = 7.9, 2.4 Hz, C), 140.09 (dd, J = 16.0, 1.7 Hz, C), 137.59 (C), 137.09 (d, J = 4.9 Hz, C), 133.48 (CH), 133.15 (CH), 129.86 (CH), 119.96 (CH), 105.88 (CH), 36.02 (d, J = 23.8 Hz, C), 34.58 (d, J = 64.2 Hz, C), 27.39 (d, J = 4.9 Hz, CH_3), 25.17 (CH_3), 10.70 (d, J = 71.8 Hz, CH_3), 5.99 (d, J = 33.4 Hz, CH_3) ppm. ^{31}P NMR (202 MHz, chloroform-*d*) δ 63.5 (d, J = 4.1 Hz), 25.3 (d, J = 4.1 Hz) ppm. HRMS-ESI *m/z* calcd. for $C_{25}H_{32}N_3OP_2Pd^+$: 556.1061, found 556.1068 $[M\text{-Br}]^+$.

13. ^{1}H and ^{13}C Spectra

^{1}H NMR ($CDCl_3$)

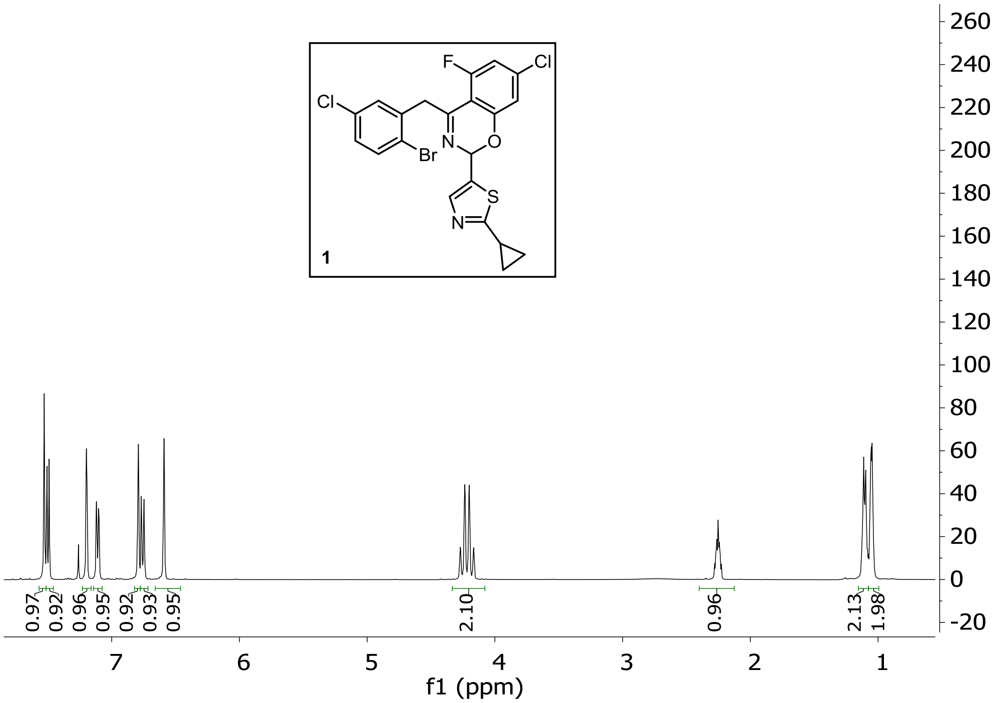

^{13}C NMR ($CDCl_3$)

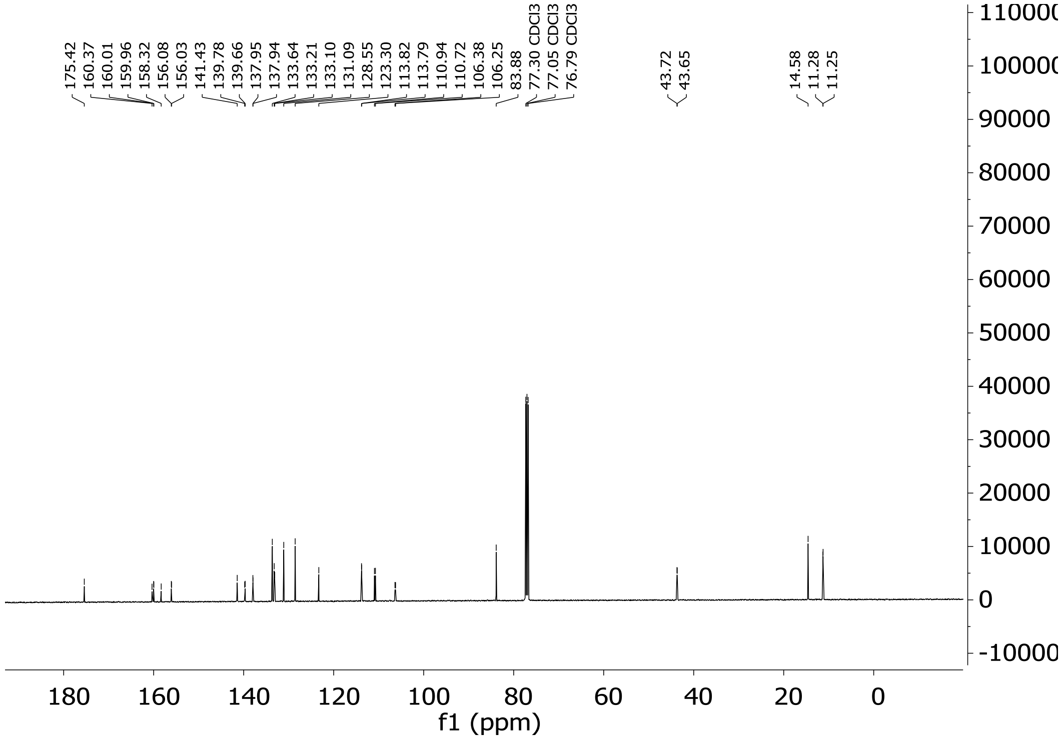

^{1}H NMR (DMSO-d_6)

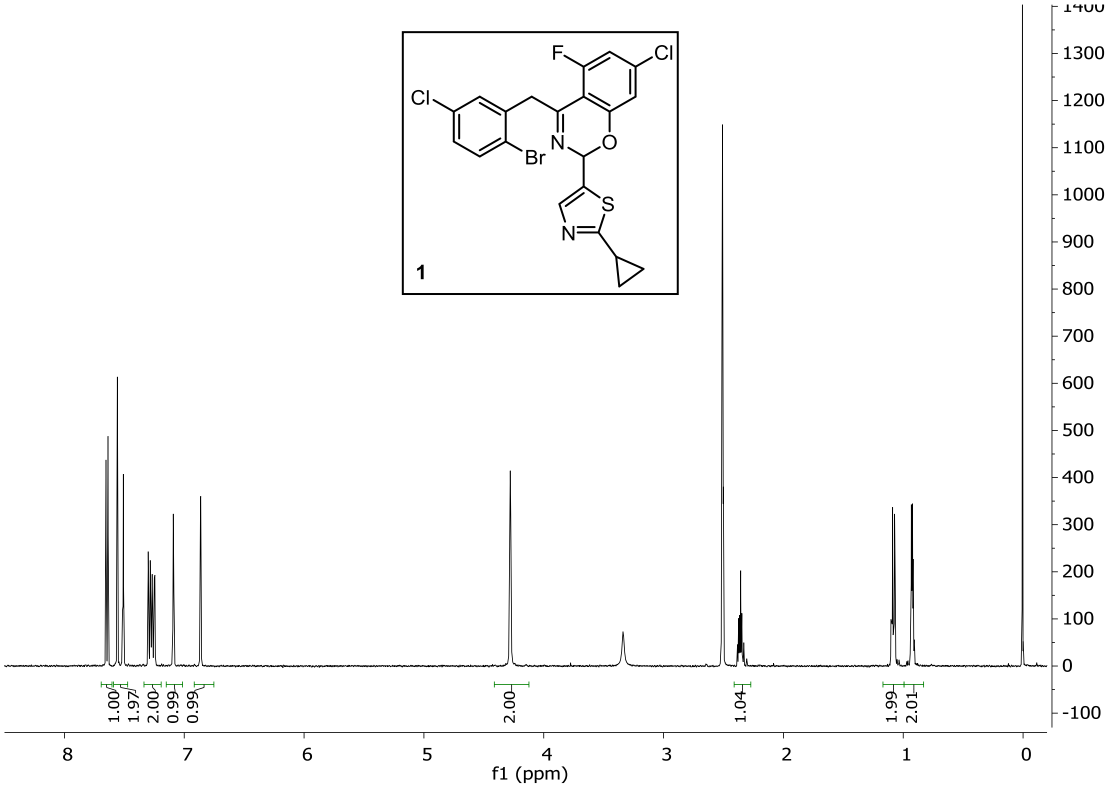

^{1}H NMR (DMSO-d_6)

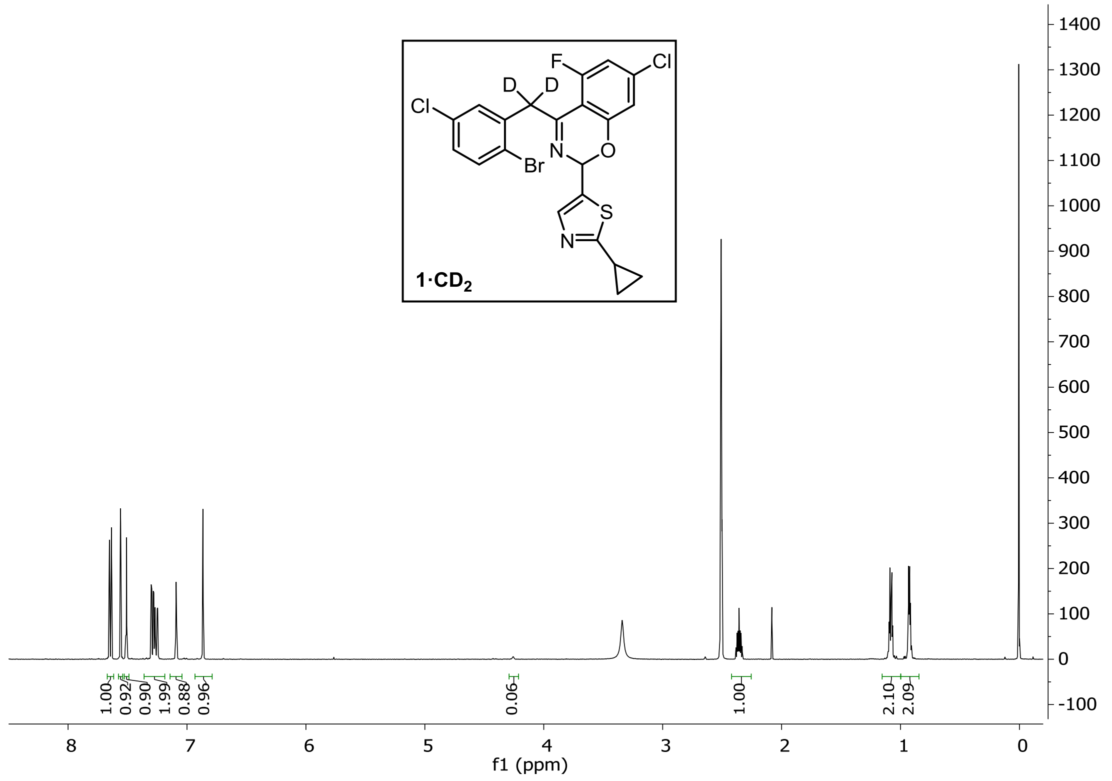

^{1}H NMR (DMSO-d_6)

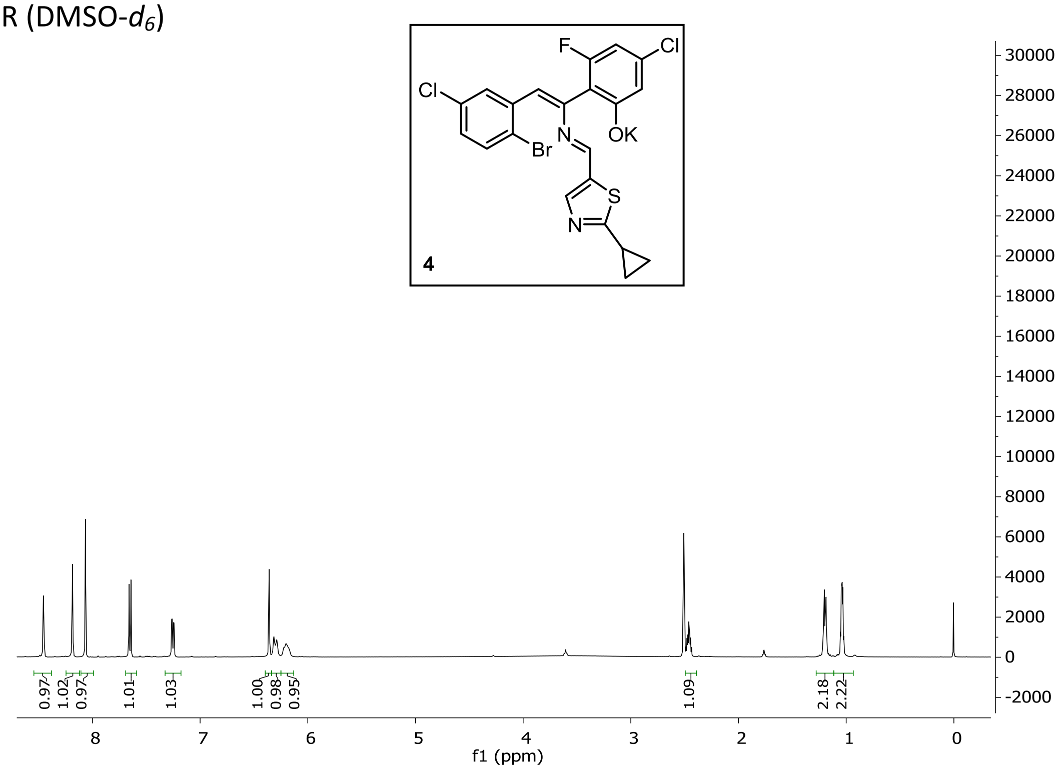

^{13}C NMR (DMSO-d_6)

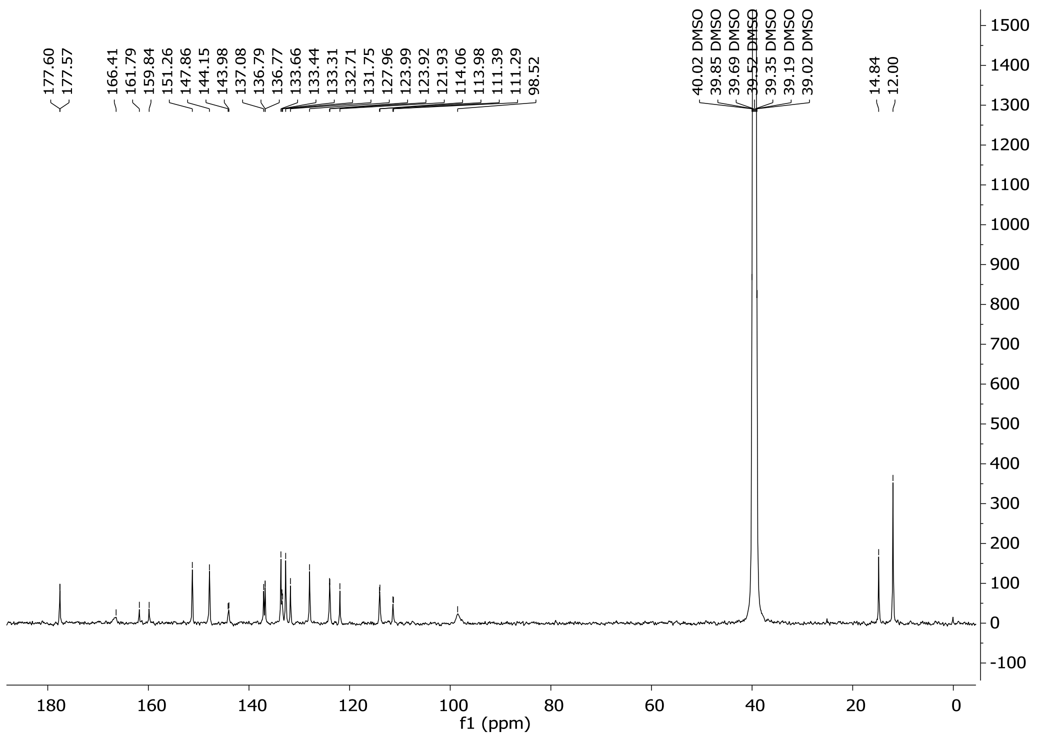

^{1}H NMR ($CDCl_3$)

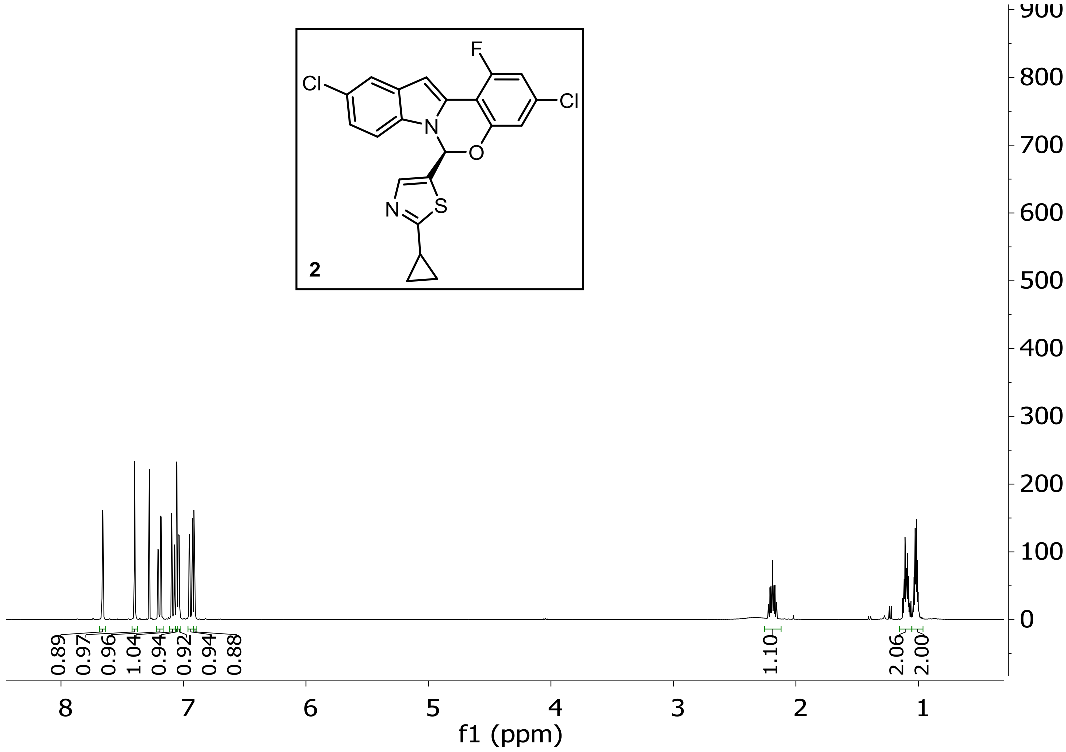

^{13}C NMR ($CDCl_3$)

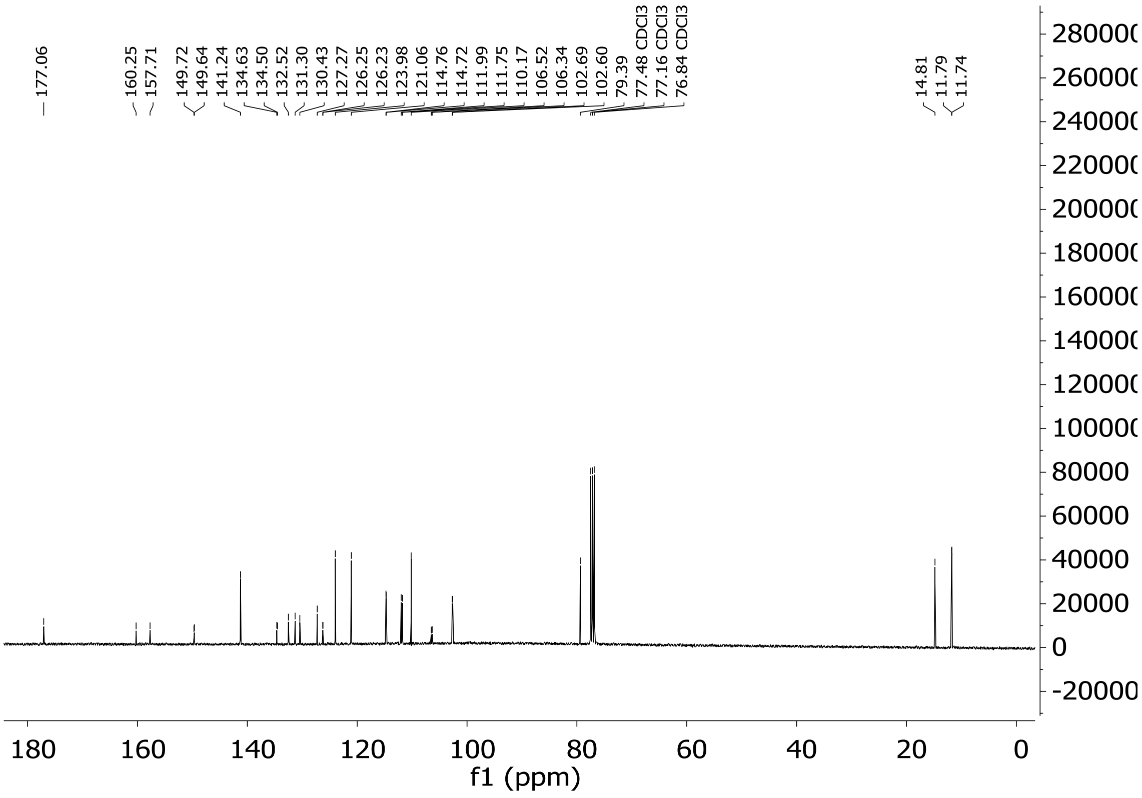

^{1}H NMR ($CDCl_3$)

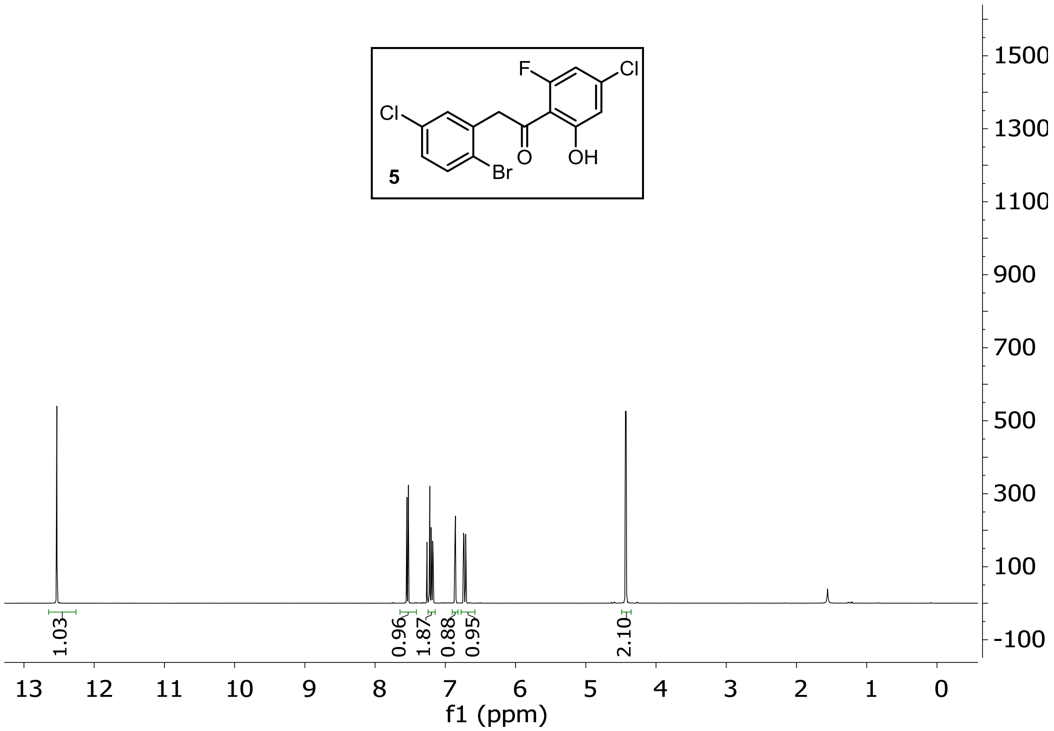

^{13}C NMR ($CDCl_3$)

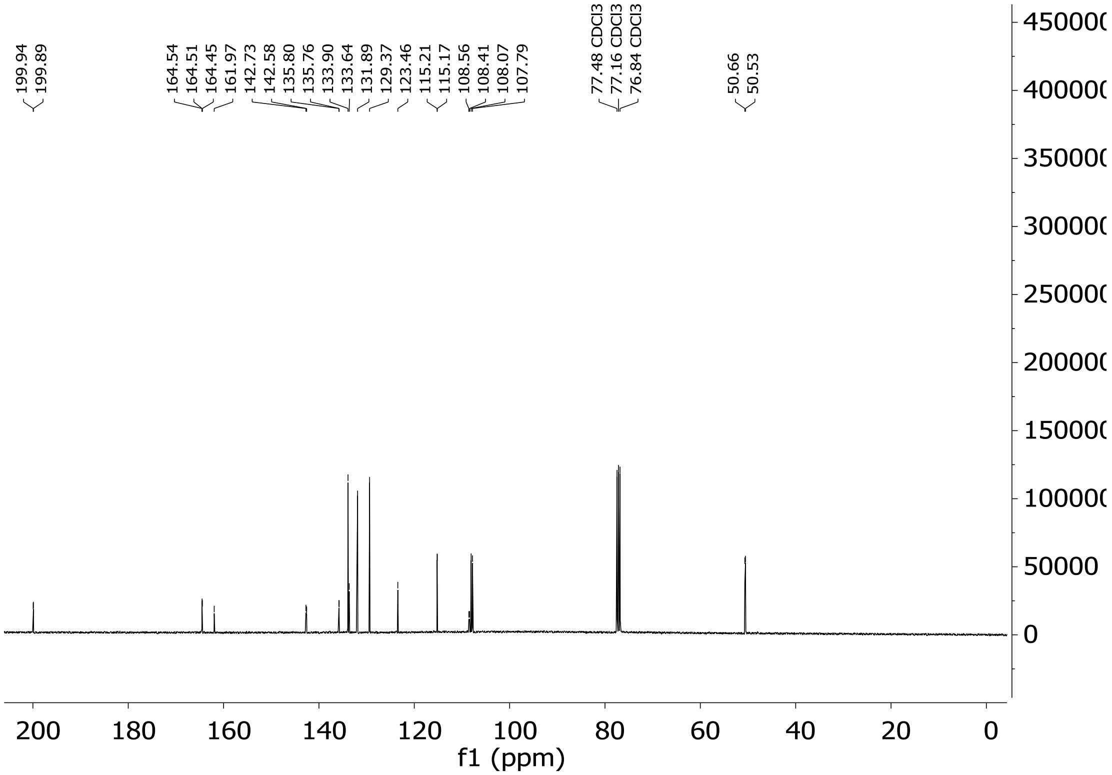

^{1}H NMR ($CDCl_3$)

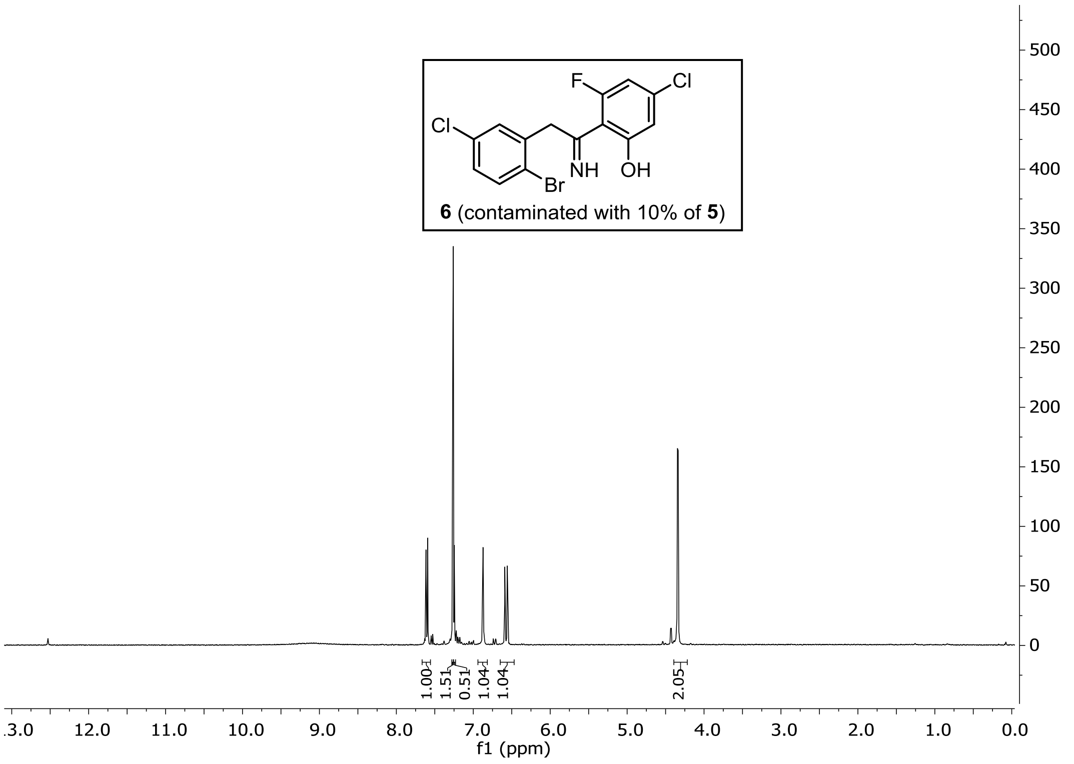

^{13}C NMR ($CDCl_3$)

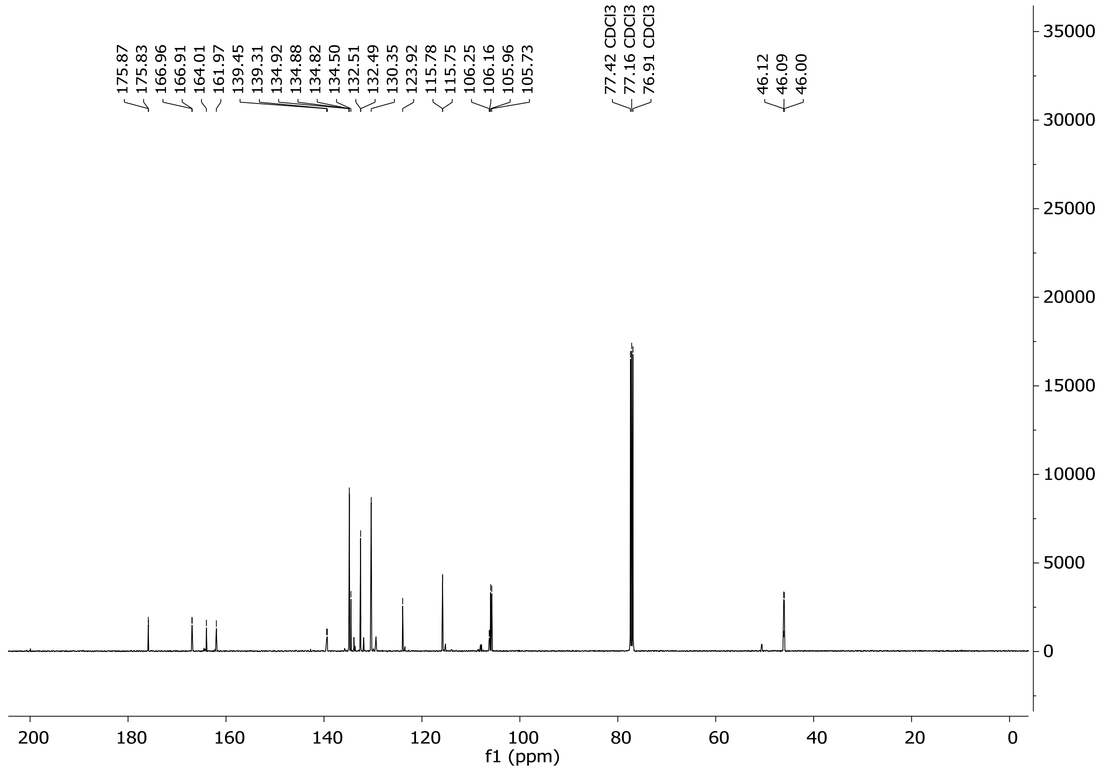

^{1}H NMR (toluene-d_8)

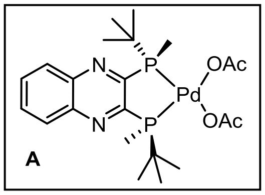

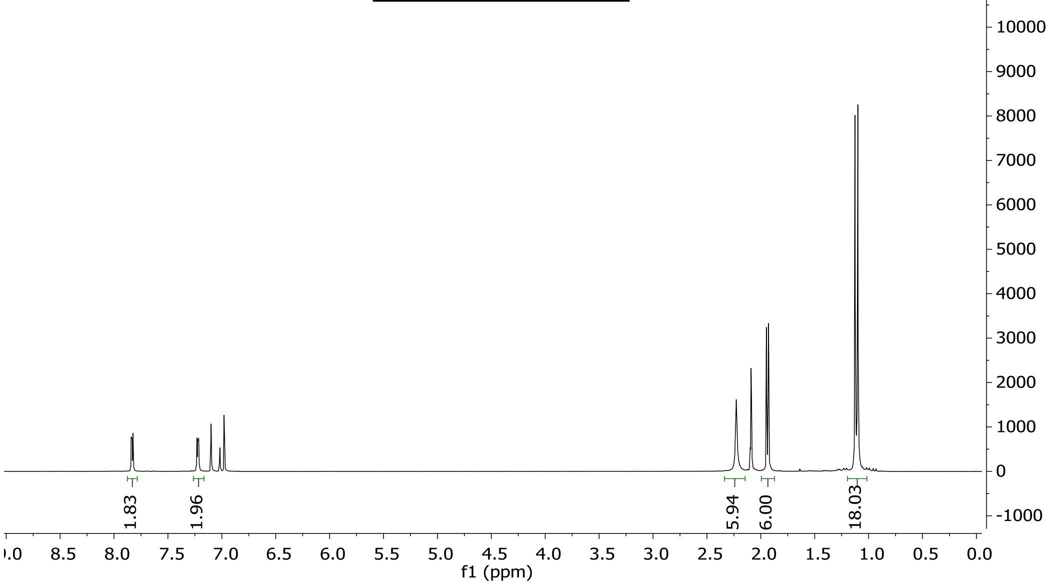

^{13}C NMR (toluene-d_8)

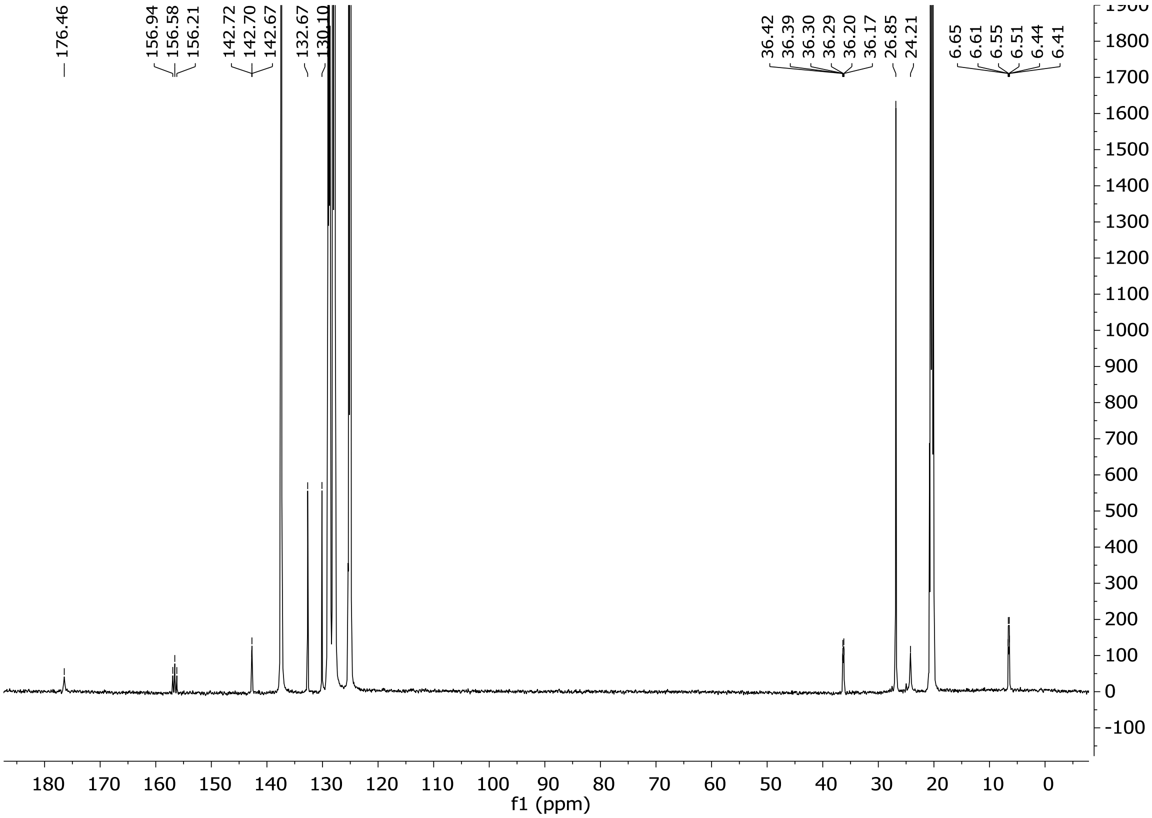

^{1}H NMR ($CDCl_3$):

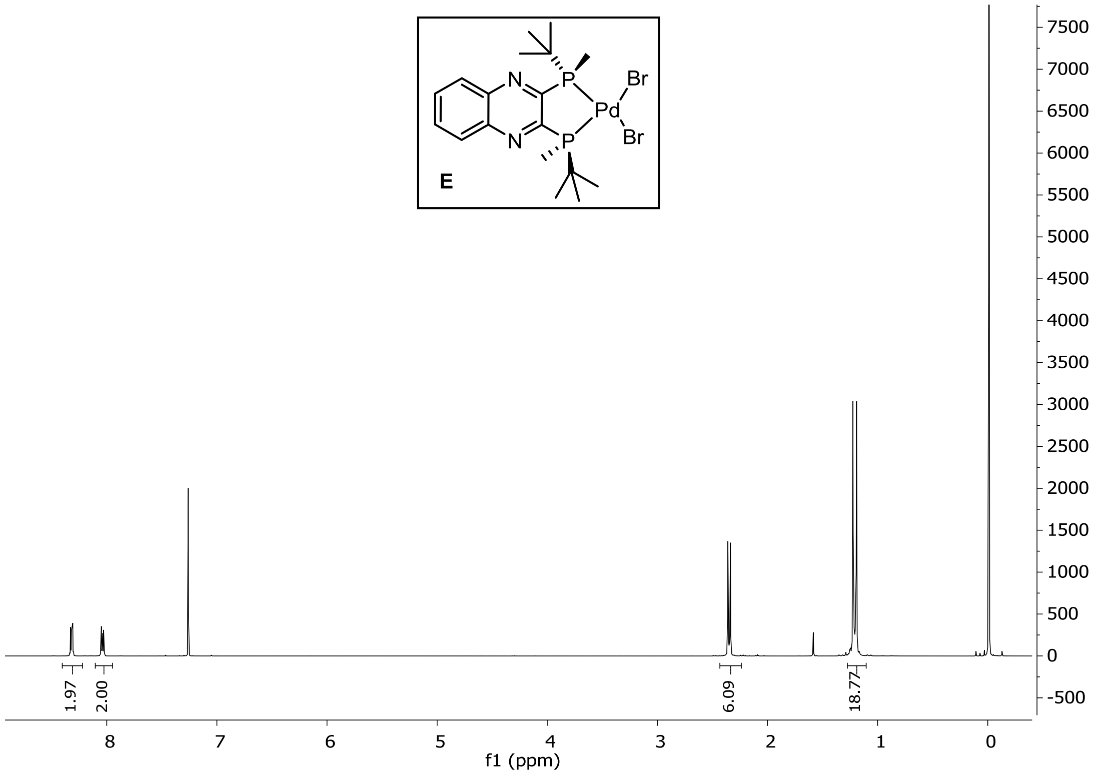

^{13}C NMR ($CDCl_3$):

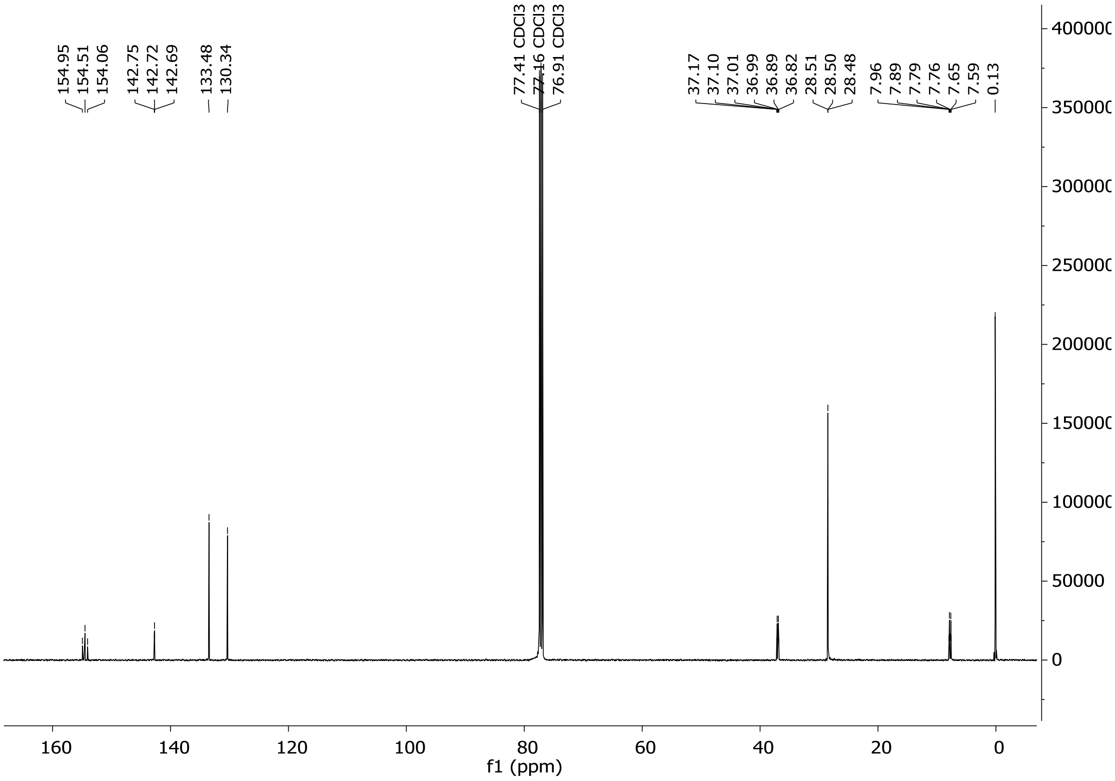

^{1}H NMR ($CDCl_3$):

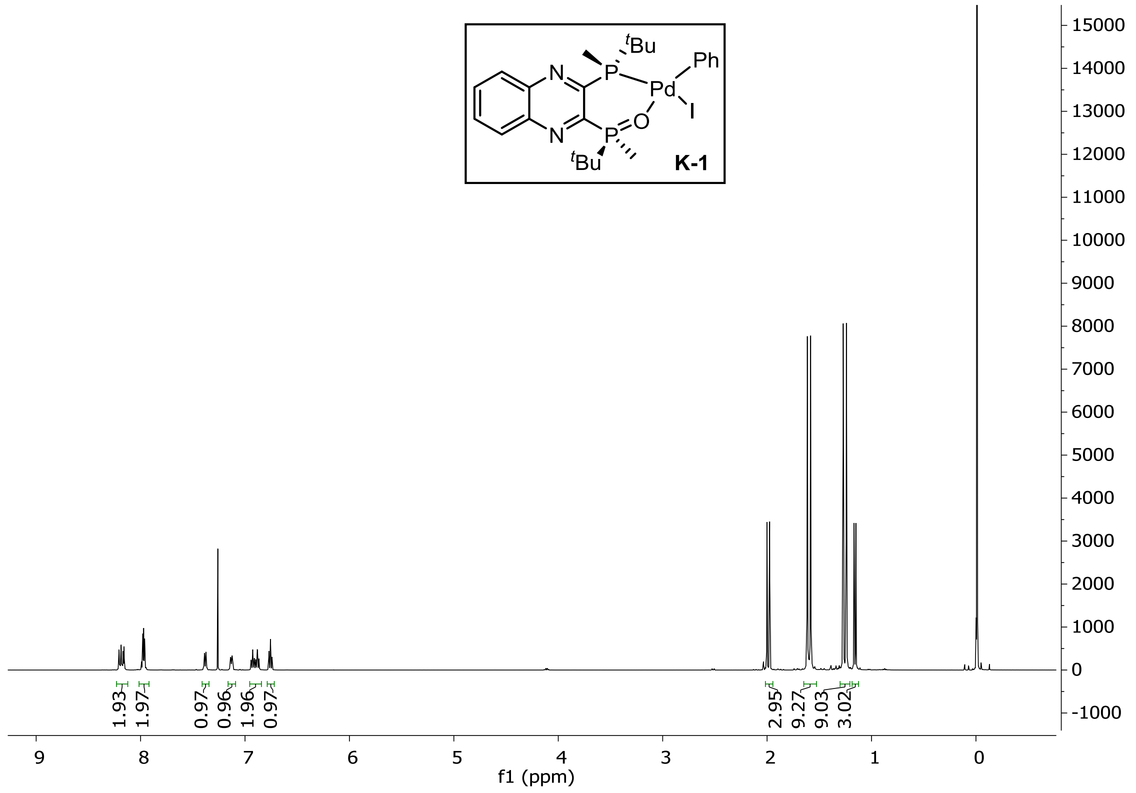

^{13}C NMR ($CDCl_3$):

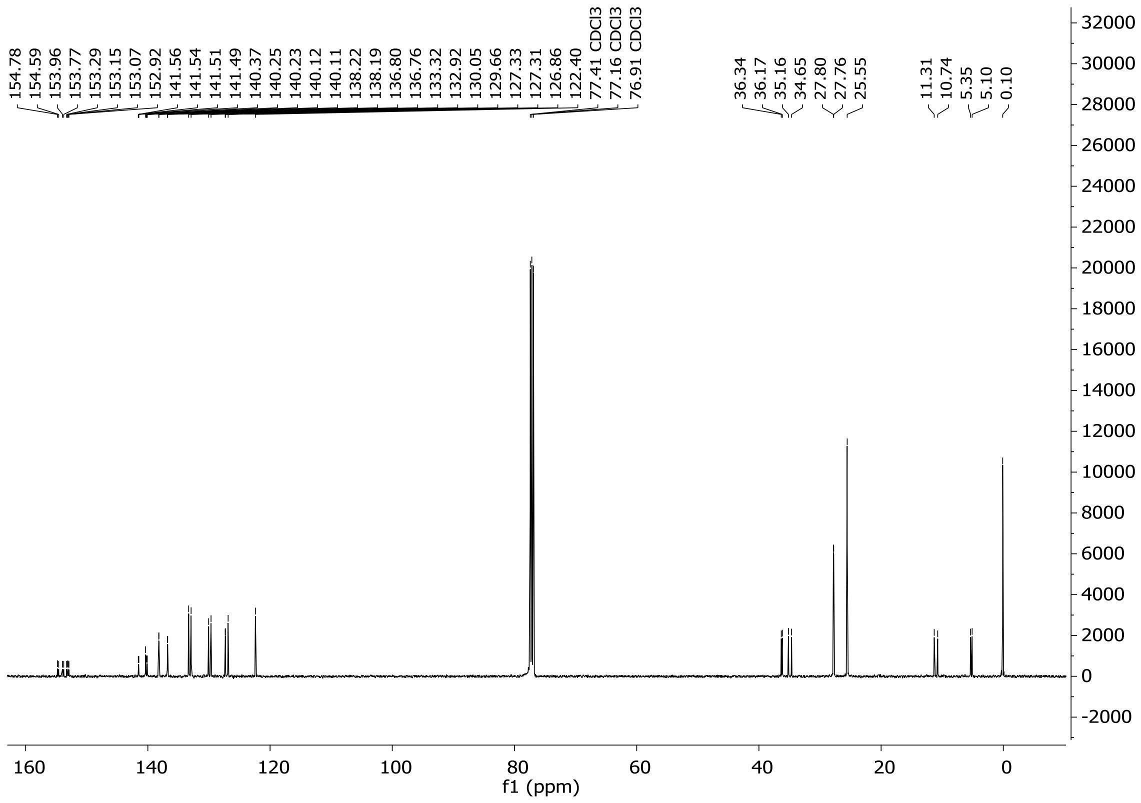

^{1}H NMR ($CDCl_3$):

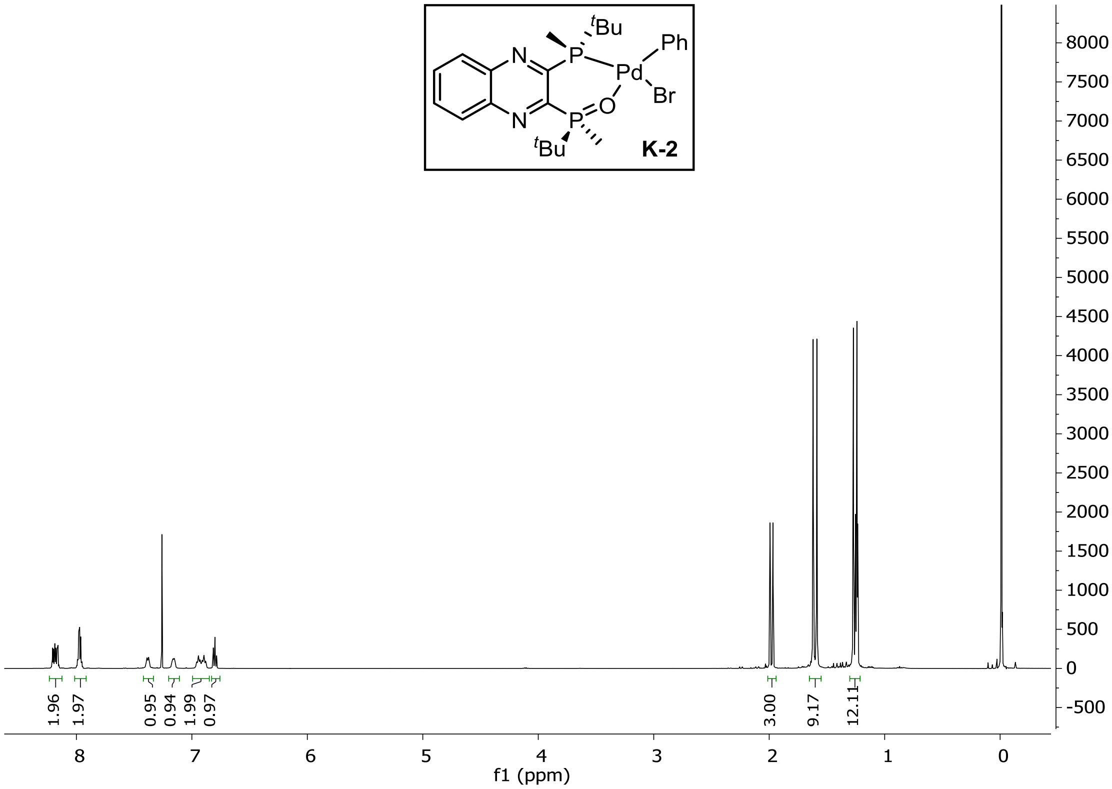

^{13}C NMR ($CDCl_3$):

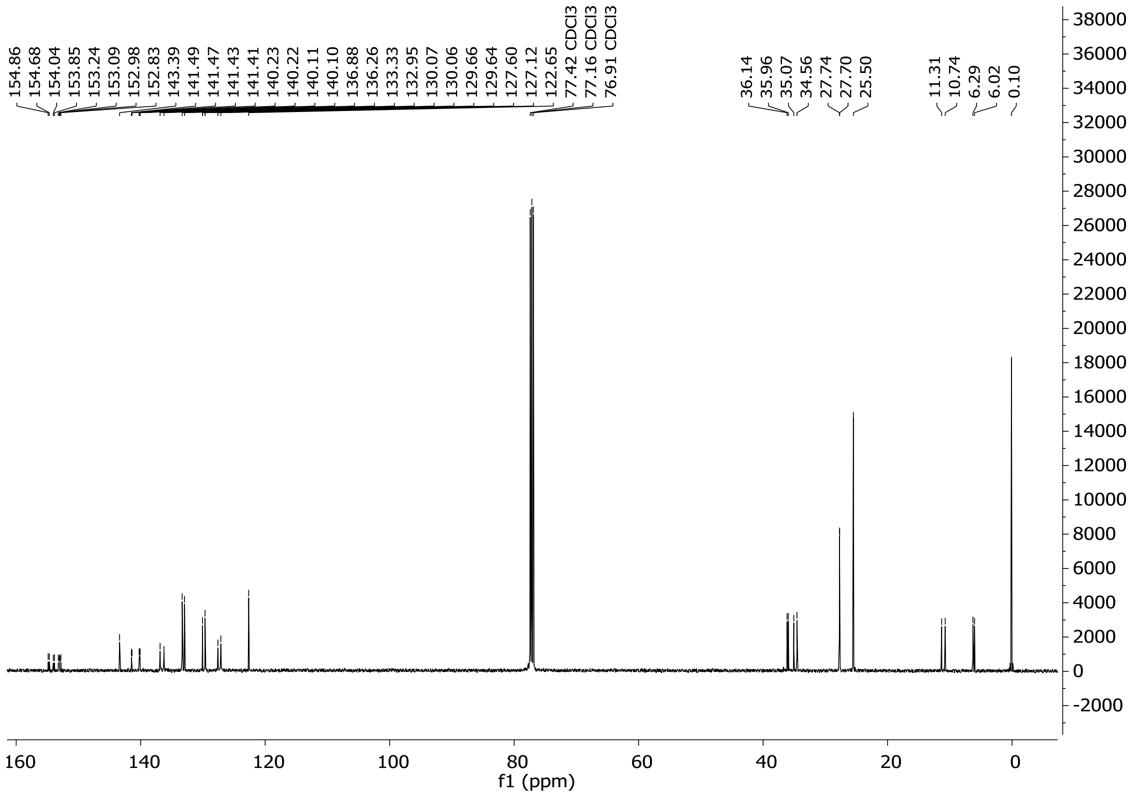

^{1}H NMR ($CDCl_3$):

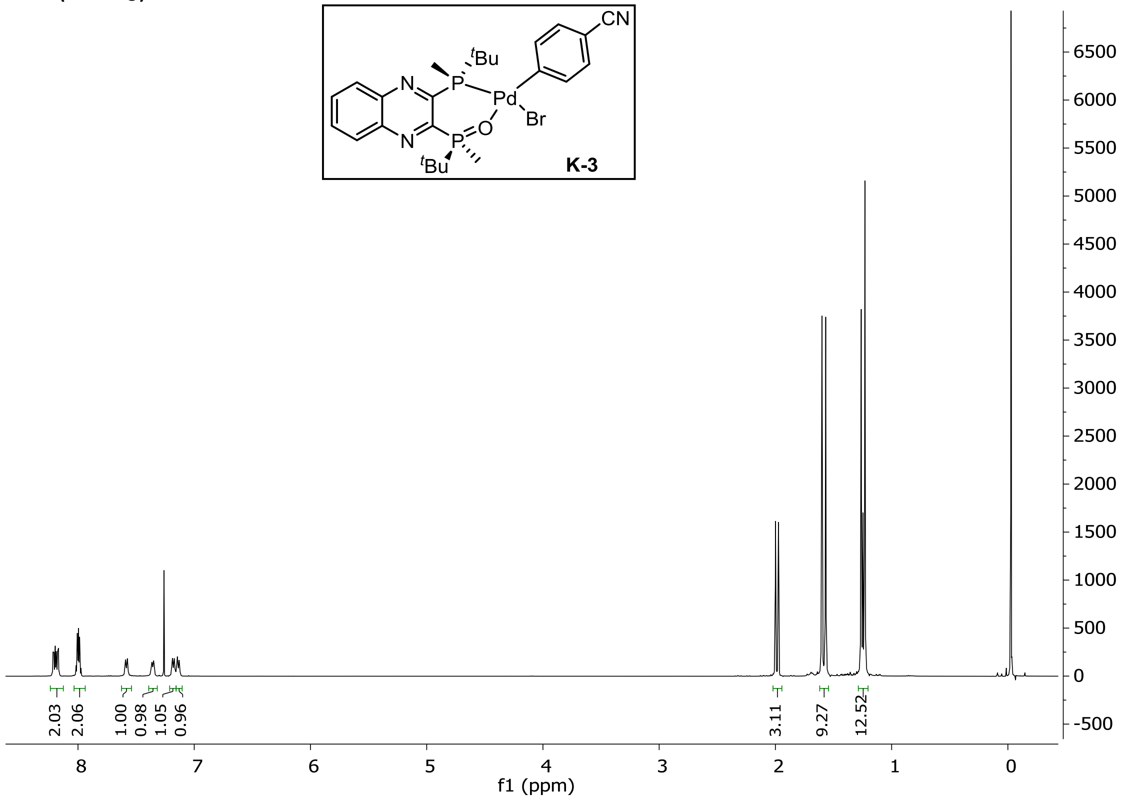

^{13}C NMR ($CDCl_3$):

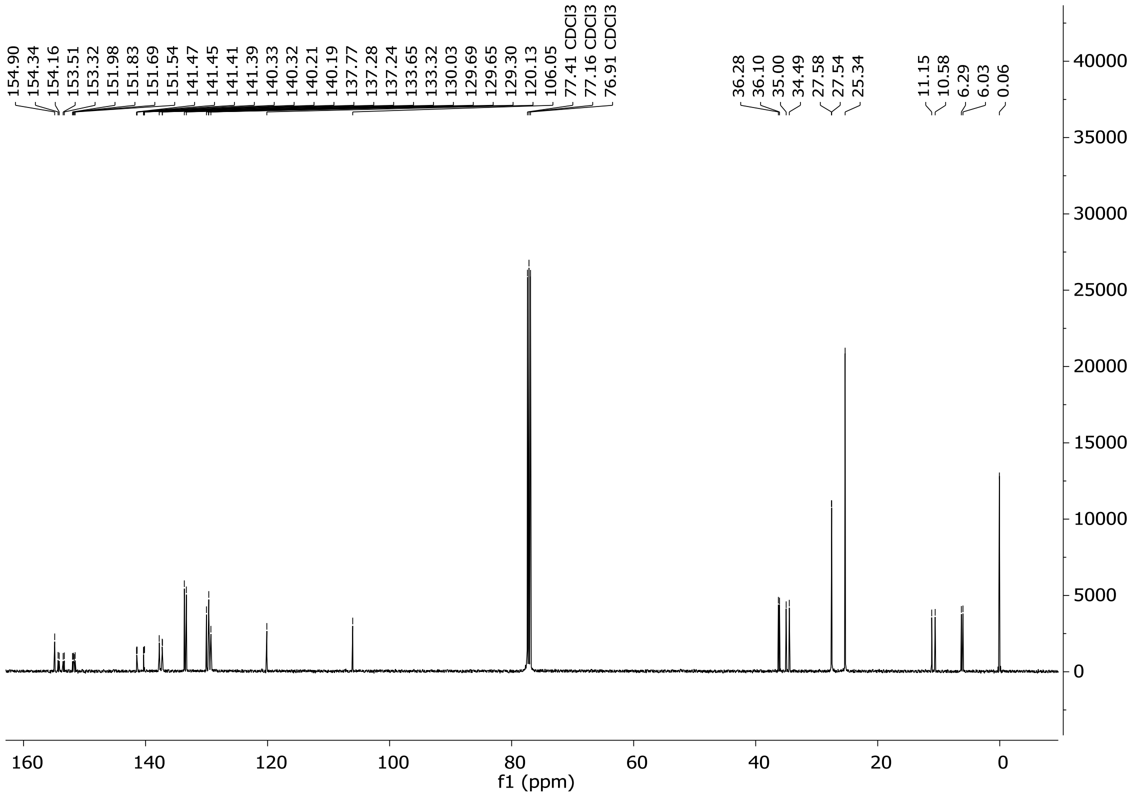

^{1}H NMR (toluene-d_8):

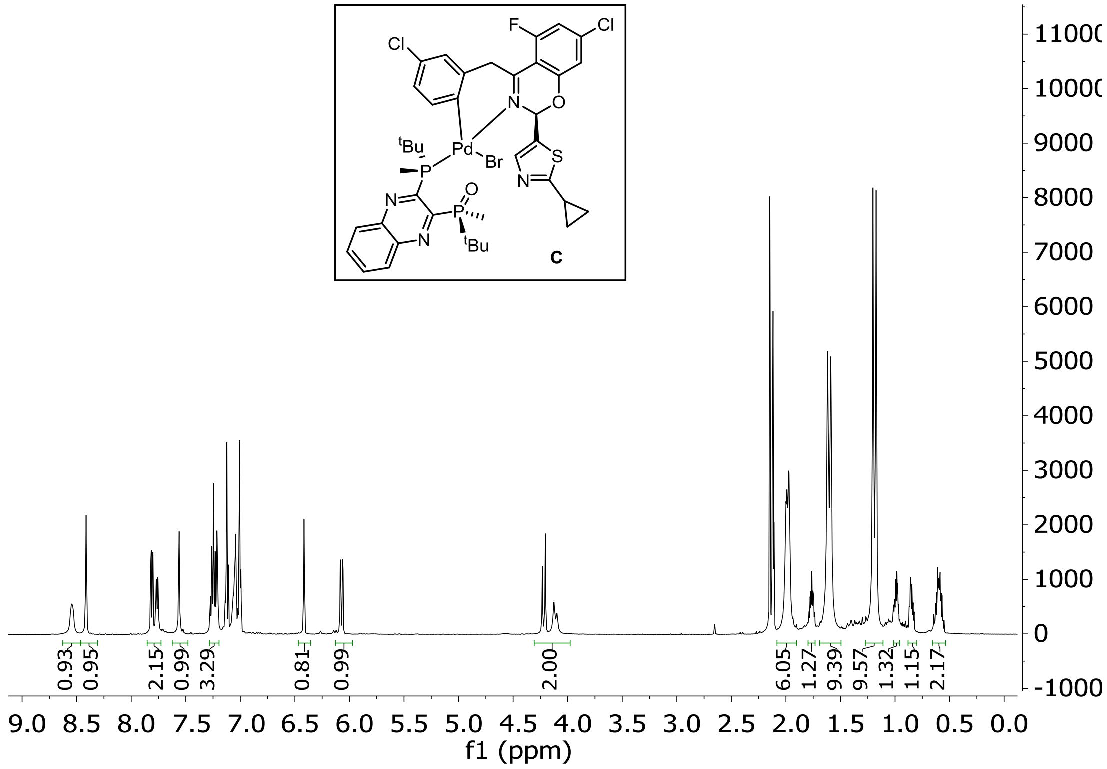

^{13}C NMR (toluene-d_8):

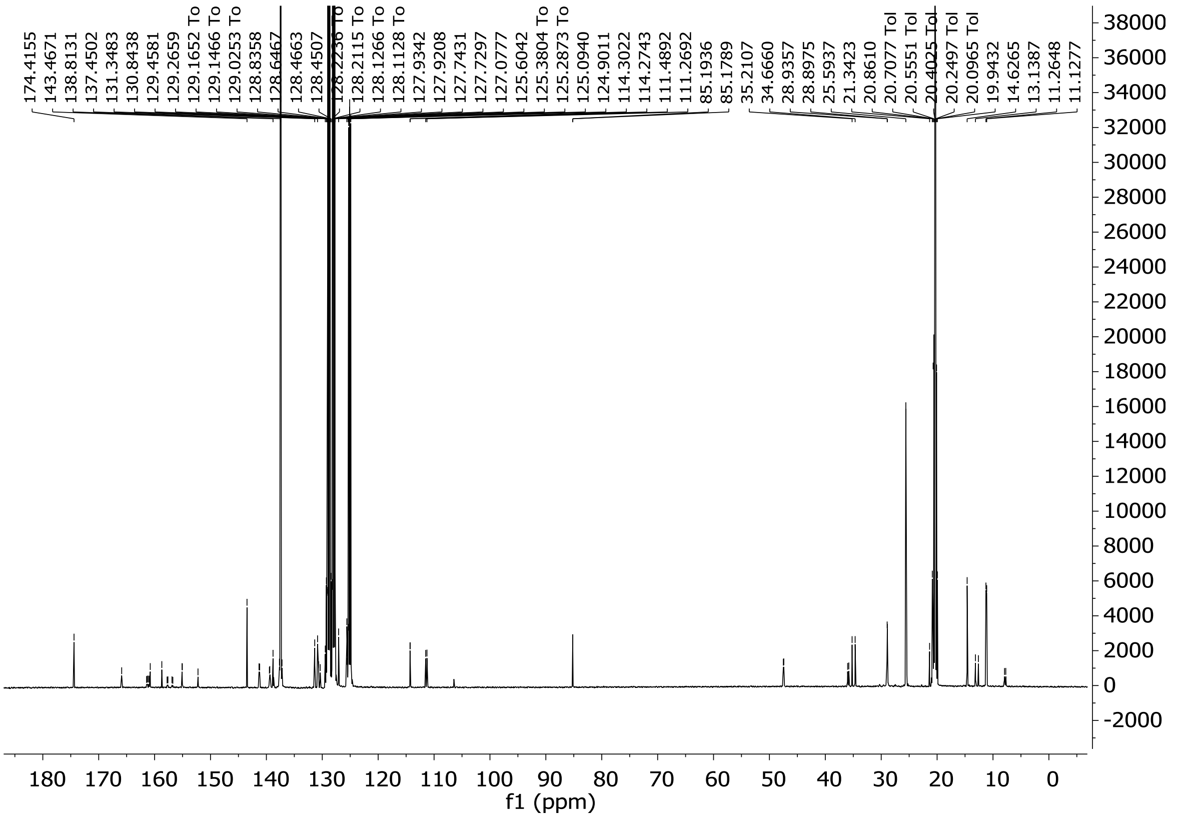

^{1}H NMR (toluene-d_8):

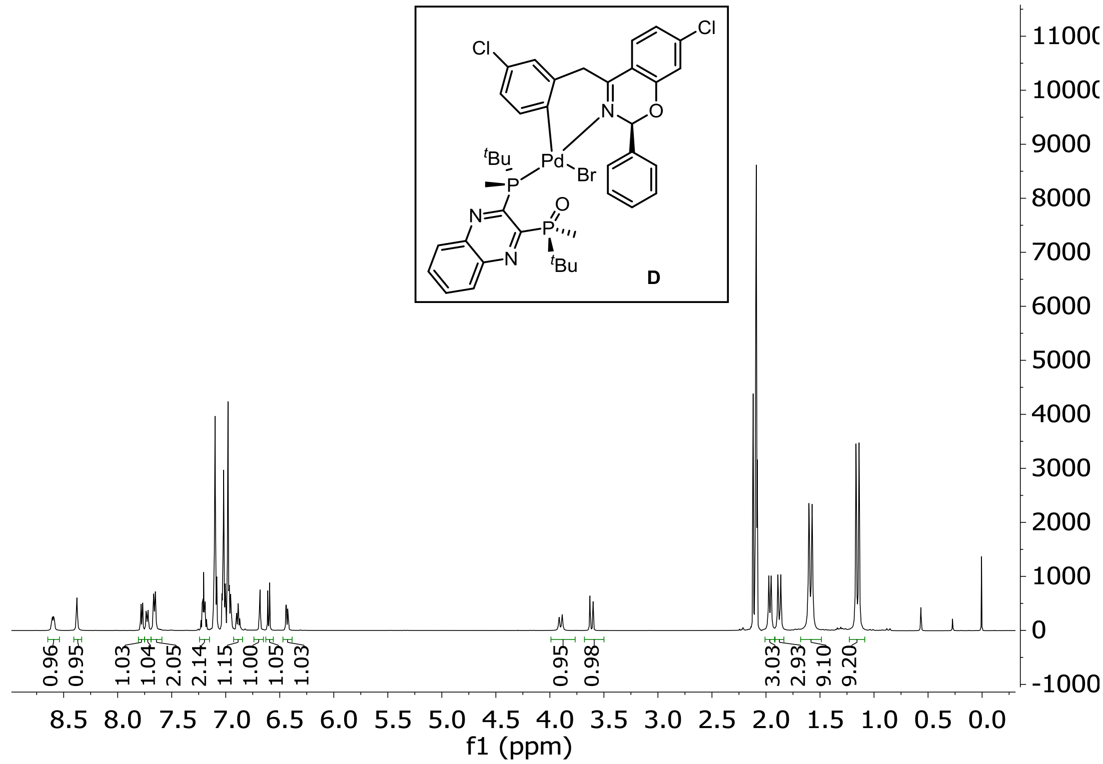

^{13}C NMR (toluene-d_8):

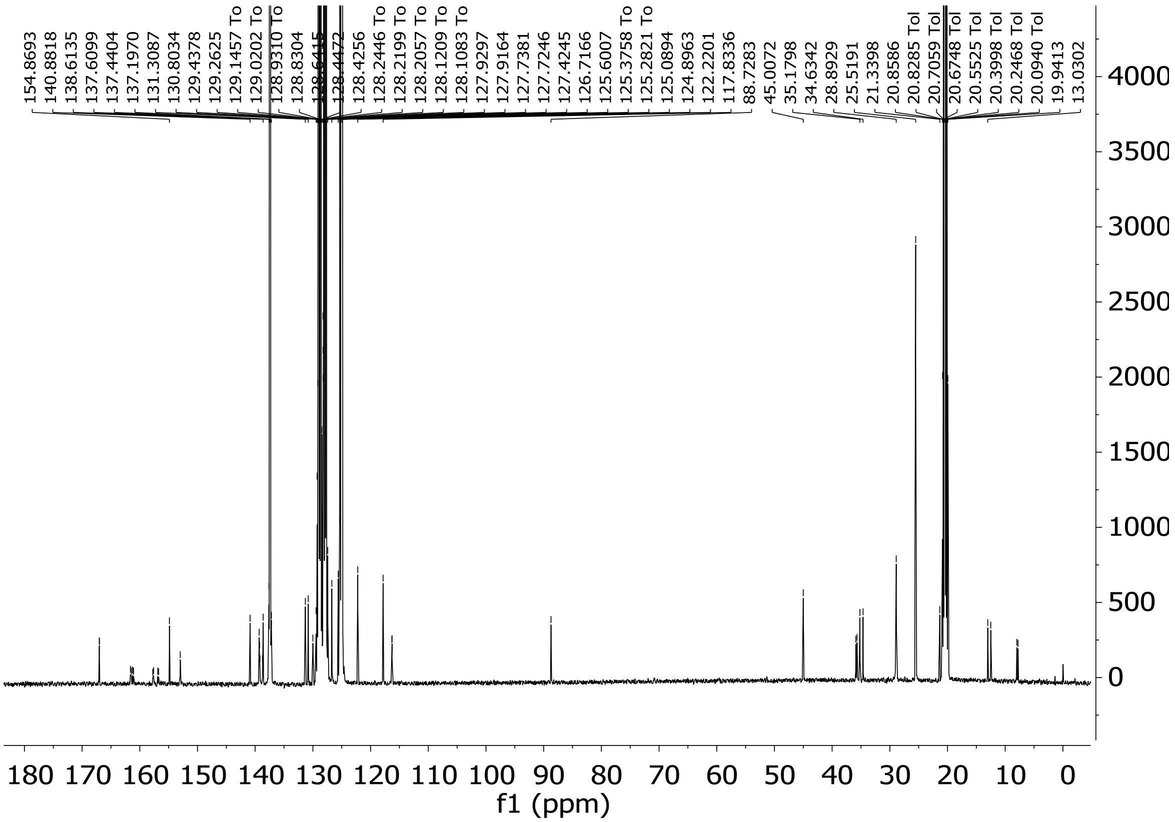

14. X-ray crystallography data

14.1 Crystal data and structure refinement for complex D (CCDC 1512973)

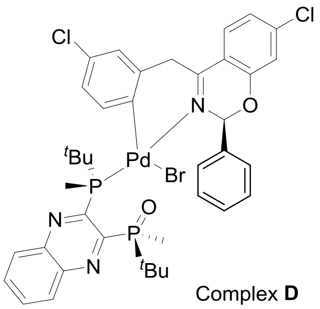

Complex **D**

A single crystal grown from toluene by slow evaporation was selected for single crystal X-ray analysis. The crystal was a small yellow plate with dimensions of 0.20 mm x 0.15 mm x 0.04 mm. Data collection was performed on a Bruker Apex II system at 100 K. The unit cell was determined to be orthorhombic in space group $P2_12_12_1$. The complex crystallized as a monotoluene solvate, with the toluene solvate found to be positionally disordered, with two orientations observed (56:44 occupancy ratio). Crystallographic data is summarized in Table 1. Absolute configuration was established by anomalous-dispersion effects in diffraction measurements on the crystal and confirmed that the stereochemistry was as shown in the scheme below. Fig. S16 shows a thermal ellipsoid representation of complex **D** toluene solvate with thermal ellipsoids set at the 50% probability level. Fig. S17 shows complex **D** with the toluene of solvation removed for clarity.

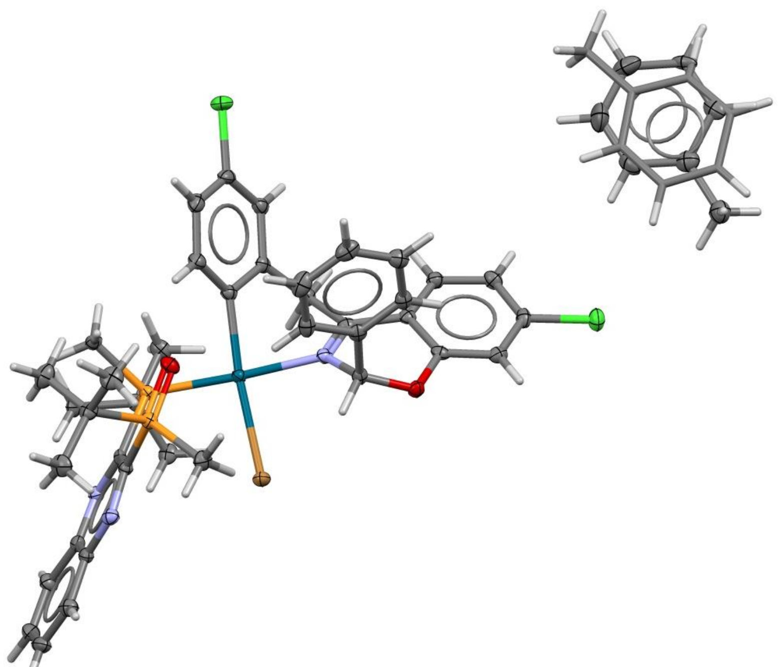

Fig. S16. Thermal ellipsoid representation of complex **D** with thermal ellipsoids set at the 50% probability level.

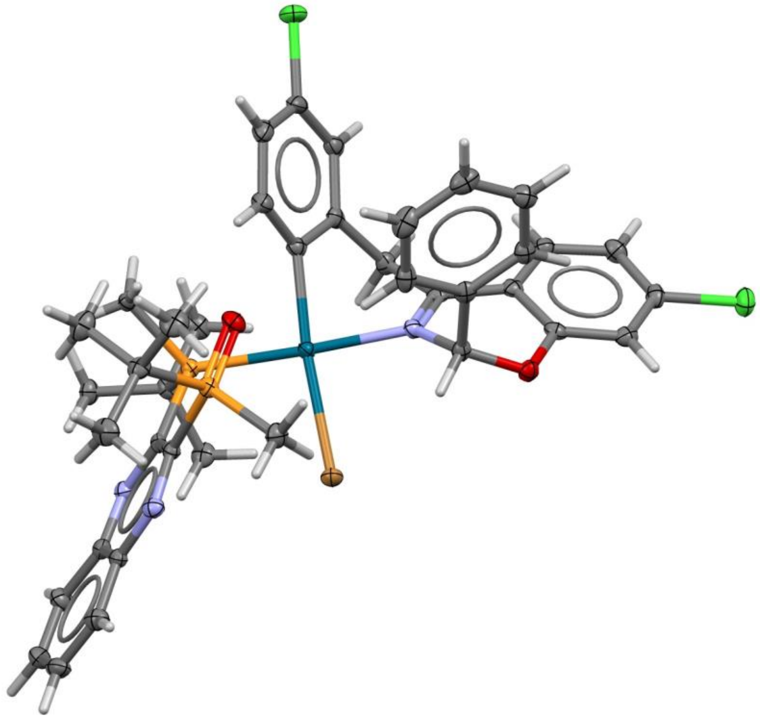

Fig. S17. Thermal ellipsoid representation of complex **D** with thermal ellipsoids set at the 50% probability level. The toluene of solvation has been removed for clarity

Table S1. Crystal Data and Structure Refinement for Complex **D** (CCDC 1512973)

Identification code	mdk052	
Empirical formula	$C_{39}H_{42}BrCl_2N_3O_2P_2Pd{\cdot}C_7H_8$	
Formula weight	996.04	
Temperature	100(2) K	
Wavelength	1.54178 Å	
Crystal system	Orthorhombic	
Space group	$P2_12_12_1$	
Unit cell dimensions	a = 12.7416(3) Å	α= 90°.
	b = 15.3629(4) Å	β= 90°.
	c = 22.5851(6) Å	γ = 90°.
Volume	4420.99(19) $Å^3$	
Z	4	
Density (calculated)	1.496 Mg/m^3	
Absorption coefficient	6.563 mm^{-1}	
F(000)	2032	
Crystal size	0.20 x 0.15 x 0.04 mm^3	
Theta range for data collection	3.479 to 67.195°.	
Index ranges	-14<=h<=15, -18<=k<=15, -19<=l<=26	
Reflections collected	28240	
Independent reflections	7828 [R(int) = 0.0379]	
Completeness to theta = 67.195°	99.5 %	
Absorption correction	Semi-empirical from equivalents	
Max. and min. transmission	0.7691 and 0.5841	
Refinement method	Full-matrix least-squares on F^2	
Data / restraints / parameters	7828 / 96 / 588	
Goodness-of-fit on F^2	1.044	
Final R indices [I>2sigma(I)]	R1 = 0.0205, wR2 = 0.0506	
R indices (all data)	R1 = 0.0214, wR2 = 0.0511	
Absolute structure parameter	-0.021(3)	
Largest diff. peak and hole	0.307 and -0.231 e.$Å^{-3}$	

14.2 Crystal data and structure refinement for complex E (CCDC 1512974)

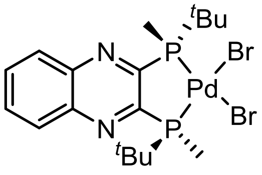

Complex **E**

A single crystal grown from dichloromethane and toluene by slow evaporation was selected for single crystal X-ray analysis. The crystal was a small yellow plate with dimensions of 0.05 mm x 0.05 mm x 0.01 mm. Data collection was performed on a Bruker Apex II system at 100 K. The unit cell was determined to be orthorhombic in space group $P2_12_12_1$. The complex crystallized as an anhydrous form with one molecule in the crystallographic asymmetric unit. Crystallographic data is summarized in Table 2. Absolute configuration was established by anomalous-dispersion effects in diffraction measurements on the crystal and confirmed that the stereochemistry was as shown in the scheme below. Fig. S18 shows a thermal ellipsoid representation of complex **E**, with thermal ellipsoids set at the 50% probability level.

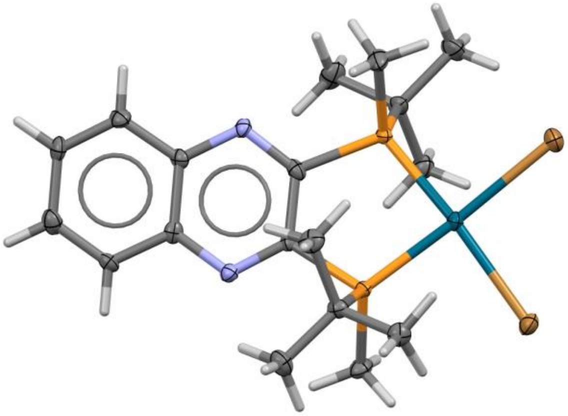

Fig. S18. Thermal ellipsoid representation of Complex **E**, with thermal ellipsoids set at the 50% probability level.

Table S2. Crystal Data and Structure Refinement for Complex **E** (CCDC 1512974)

Identification code	mdk104	
Empirical formula	$C_{18}H_{28}Br_2N_2P_2Pd$	
Formula weight	600.58	
Temperature	100(2) K	
Wavelength	1.54178 Å	
Crystal system	Orthorhombic	
Space group	$P2_12_12_1$	
Unit cell dimensions	a = 7.6802(4) Å	α= 90°.
	b = 15.4523(7) Å	β= 90°.
	c = 18.3830(9) Å	γ = 90°.
Volume	2181.63(18) $Å^3$	
Z	4	
Density (calculated)	1.829 Mg/m^3	
Absorption coefficient	12.567 mm^{-1}	
F(000)	1184	
Crystal size	0.050 x 0.050 x 0.010 mm^3	
Theta range for data collection	3.737 to 66.603°.	
Index ranges	-8<=h<=9, -18<=k<=17, -21<=l<=21	
Reflections collected	13372	
Independent reflections	3816 [R(int) = 0.0415]	
Completeness to theta = 66.500°	99.7 %	
Absorption correction	Semi-empirical from equivalents	
Max. and min. transmission	0.882 and 0.665	
Refinement method	Full-matrix least-squares on F^2	
Data / restraints / parameters	3816 / 0 / 234	
Goodness-of-fit on F^2	1.085	
Final R indices [I>2sigma(I)]	R1 = 0.0246, wR2 = 0.0556	
R indices (all data)	R1 = 0.0263, wR2 = 0.0570	
Absolute structure parameter	-0.013(6)	
Largest diff. peak and hole	0.419 and -0.422 e.$Å^{-3}$	

14.3 Crystal data and structure refinement for pre-cataylst K3 (CCDC 1512975)

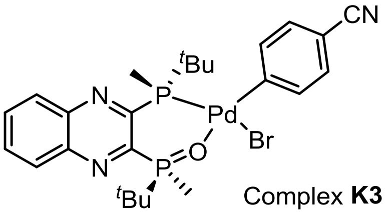

A single crystal grown from toluene by slow evaporation was selected for single crystal X-ray analysis. The crystal was a pale yellow block with dimensions of 0.3 mm x 0.2 mm x 0.1 mm. Data collection was performed on a Bruker Apex II system at 100 K. The unit cell was determined to be monoclinic in space group C2. The complex crystallized as an anhydrous form with one molecule in the asymmetric unit. Crystallographic data is summarized in Table 3. Absolute configuration was established by anomalous-dispersion effects in diffraction measurements on the crystal and confirmed that the stereochemistry was as shown in the scheme below. Fig. S19 shows a thermal ellipsoid representation of pre-catalyst **J3**, with thermal ellipsoids set at the 50% probability level.

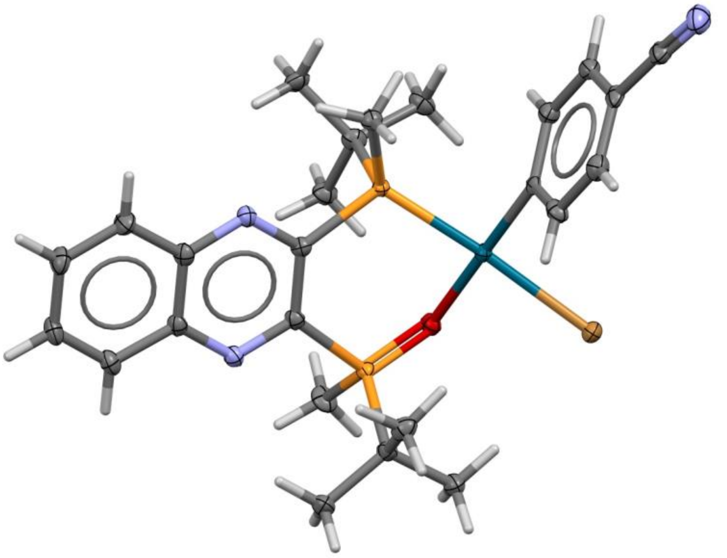

Fig. S19. Thermal ellipsoid representation of pre-catalyst **K3** with thermal ellipsoids set at the 50% probability level.

Table S3. Crystal Data and Structure Refinement for Pre-catalyst **K3** (CCDC 1512975)

Identification code	mdk105	
Empirical formula	$C_{25}H_{32}BrN_3OP_2Pd$	
Formula weight	638.78	
Temperature	100(2) K	
Wavelength	1.54178 Å	
Crystal system	Monoclinic	
Space group	C2	
Unit cell dimensions	a = 23.4340(11) Å	α= 90°.
	b = 10.7208(5) Å	β= 95.3448(14)°.
	c = 10.6091(5) Å	γ = 90°.
Volume	2653.7(2) Å^3	
Z	4	
Density (calculated)	1.599 Mg/m^3	
Absorption coefficient	8.720 mm^{-1}	
F(000)	1288	
Crystal size	0.300 x 0.200 x 0.100 mm^3	
Theta range for data collection	3.789 to 66.577°.	
Index ranges	-27<=h<=27, -12<=k<=12, -11<=l<=12	
Reflections collected	23472	
Independent reflections	4648 [R(int) = 0.0414]	
Completeness to theta = 66.577°	98.9 %	
Absorption correction	Semi-empirical from equivalents	
Max. and min. transmission	0.418 and 0.289	
Refinement method	Full-matrix least-squares on F^2	
Data / restraints / parameters	4648 / 1 / 306	
Goodness-of-fit on F^2	1.076	
Final R indices [I>2sigma(I)]	R1 = 0.0229, wR2 = 0.0562	
R indices (all data)	R1 = 0.0234, wR2 = 0.0566	
Absolute structure parameter	-0.016(4)	
Largest diff. peak and hole	0.778 and -0.427 e.Å^{-3}	

15. Structural Determination of complex C and D by NMR

15.1 NMR data for complex **C**

^{1}H NMR (toluene-d_8):

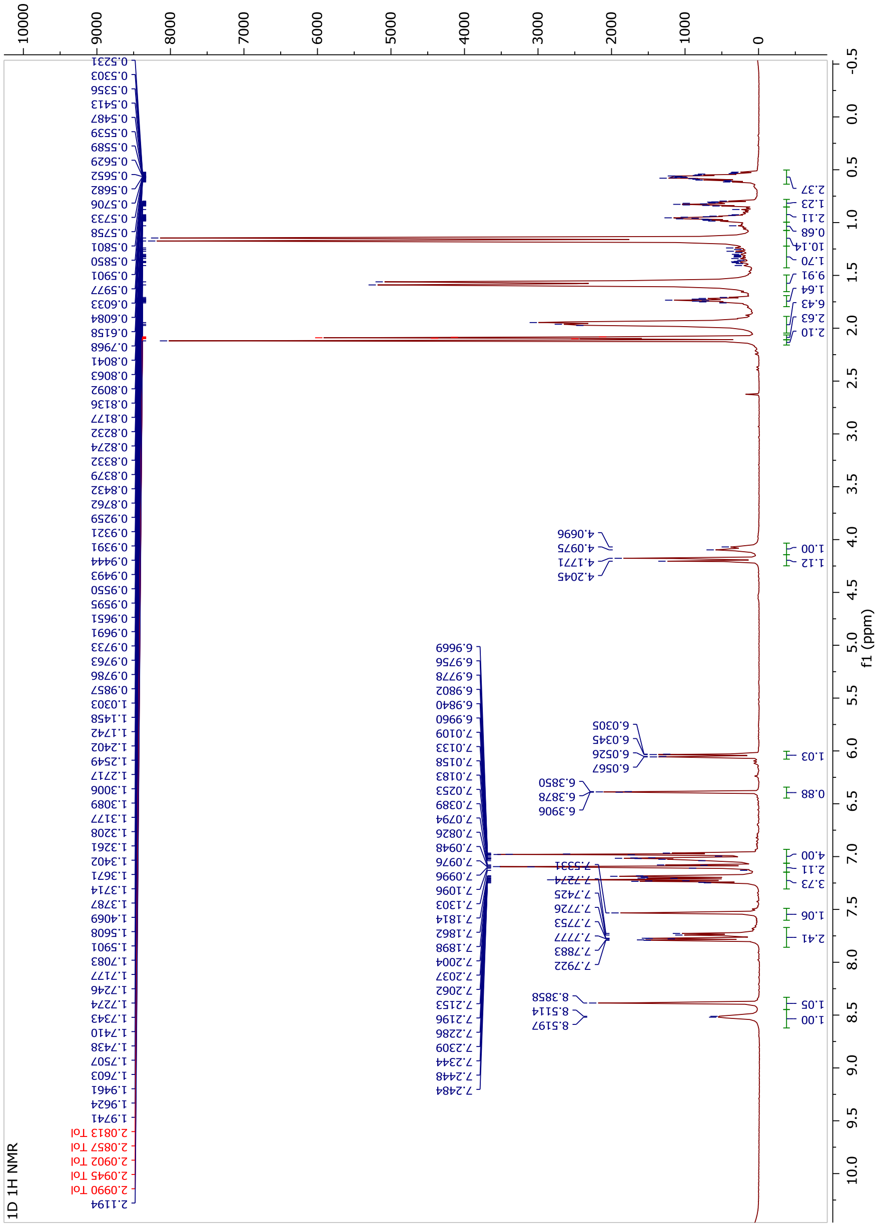

Fig. S20. ^{1}H NMR spectrum of oxidative addition complex **C** acquired at 500 MHz using the pulse program *zg30* from the Bruker pulse sequence library. A spectral width of 5498.53 Hz was acquired with 64K data points.

^{13}C NMR (toluene-d_8):

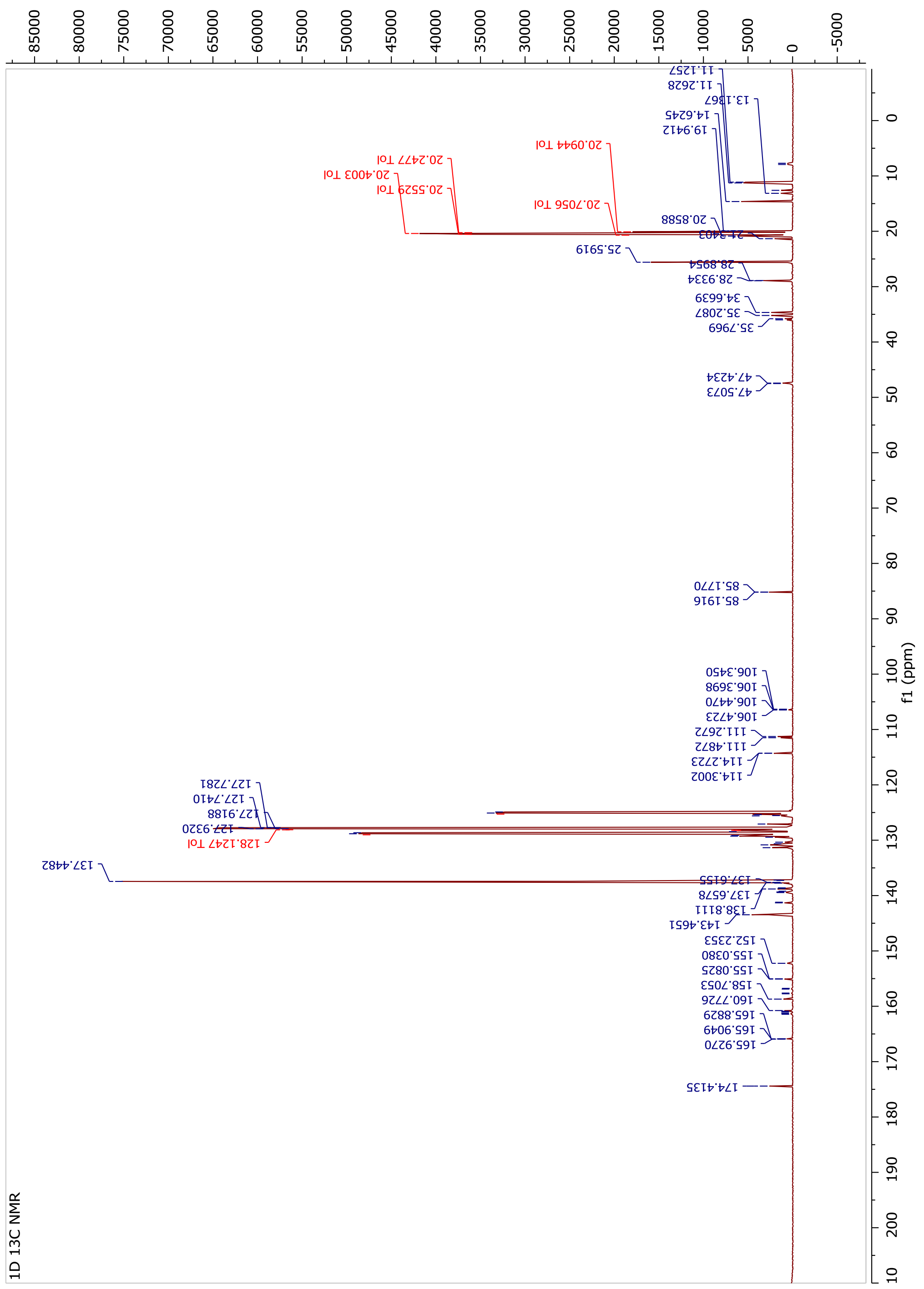

Fig. S21. ^{13}C NMR spectrum of oxidative addition complex **C** acquired at 126 MHz using the pulse program *zgpg30* from the Bruker pulse sequence library. A spectral width of 27,573.53 Hz was acquired with 128K data points.

^{1}H-^{1}H COSY (toluene-d_8):

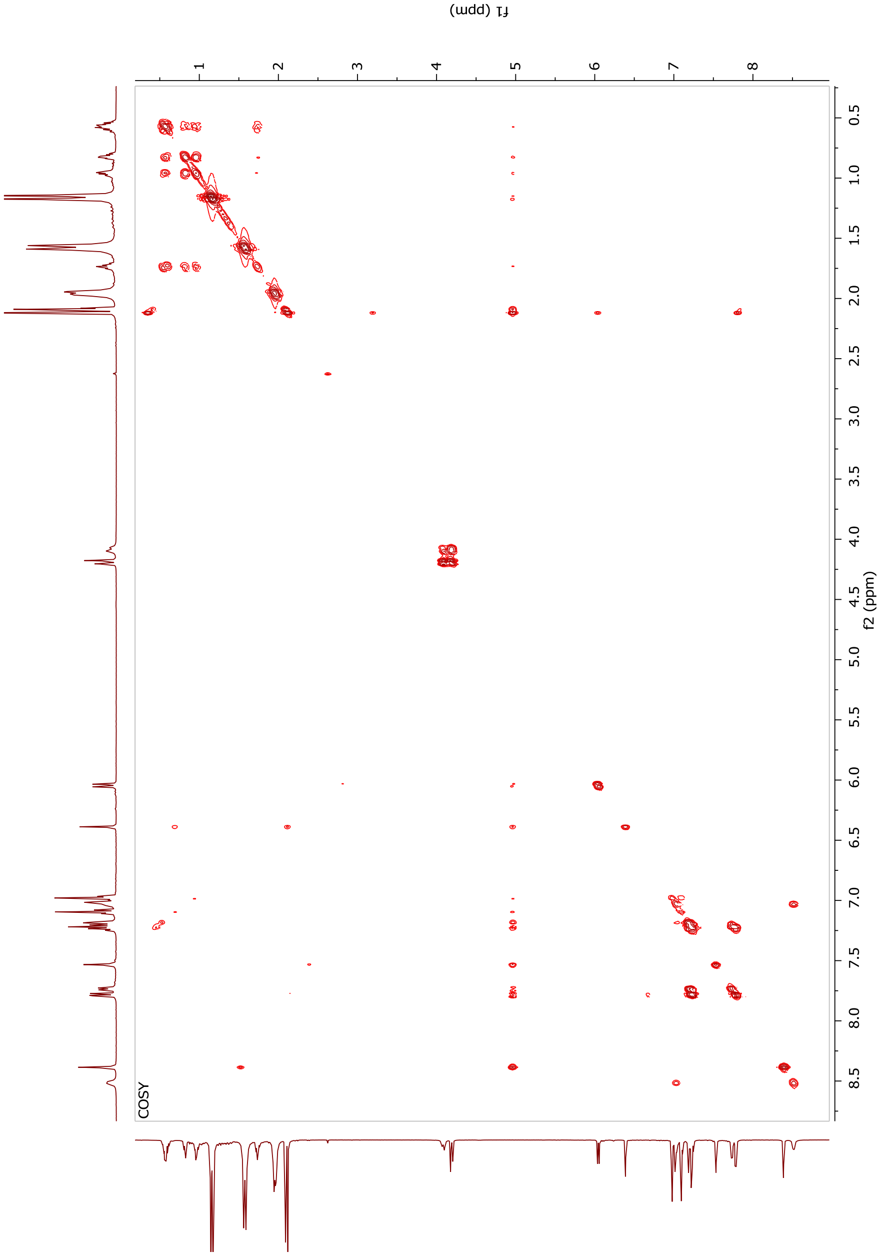

Fig. S22. 2D COSY spectrum of oxidative addition complex **C** acquired using the pulse program *cosygpqf* from the Bruker pulse sequence library. Data were acquired at 600 MHz using 2024 × 256 points with 4 transients accumulated per t_1 increment. A delay of 1 s was employed between transients, giving an overall acquisition time of 38 min 12 s. Spectral windows in both F2 and F1 were 5,151.099 Hz. Data were processed by linear prediction to 512 in F1. Prior to Fourier transform sine apodization and zero-filling to 4096 points in F2 and 1024 points in F1 were applied in both dimensions.

^{1}H-^{13}C Pure Shift-Multiplicity Edited-HSQC (toluene-*d*$_8$):

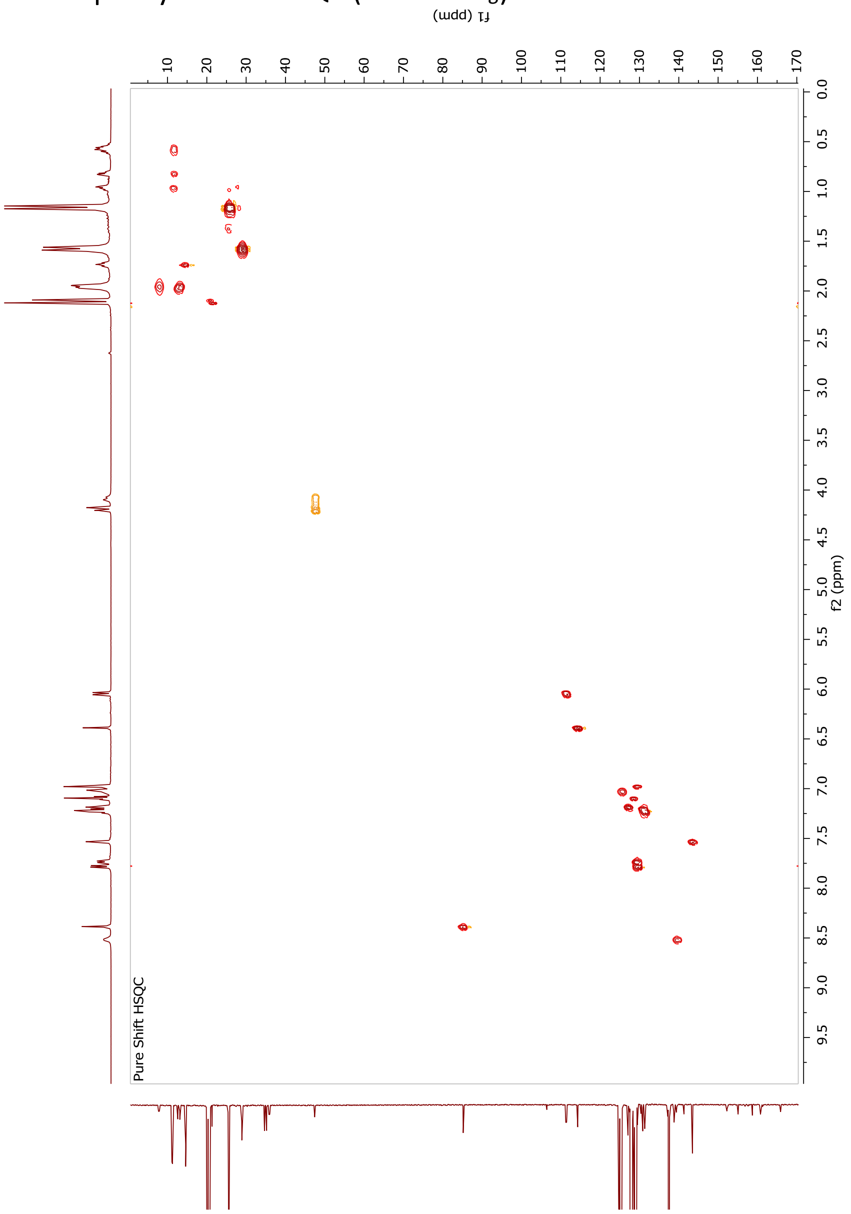

Fig. S23. 2D Pure Shift-Multiplicity Edited-HSQC spectrum of oxidative addition complex **C**. Methine and methyl resonances are shown in red; methylenes are shown in orange. Data were acquired at 500 MHz using 2048 × 256 points with 4 transients accumulated per t_1 increment. A delay of 1 s was employed between transients, giving an overall acquisition time of 23 min 43 s. The $^{1}J_{CH}$ delay was optimized for 145 Hz. Acquisition t_2 time was 0.204 s and the number of loops for homonuclear, BIRD-based acquisition was set to 8. Spectral windows in both F2 and F1 were 5,000.0 Hz and 21,369.4 Hz, respectively. Prior to Fourier transform sine squared apodization and zero-filling to 4096 points in F2 and 1024 points in F1 were applied in both dimensions.

^{1}H-^{13}C HMBC (toluene-d_8):

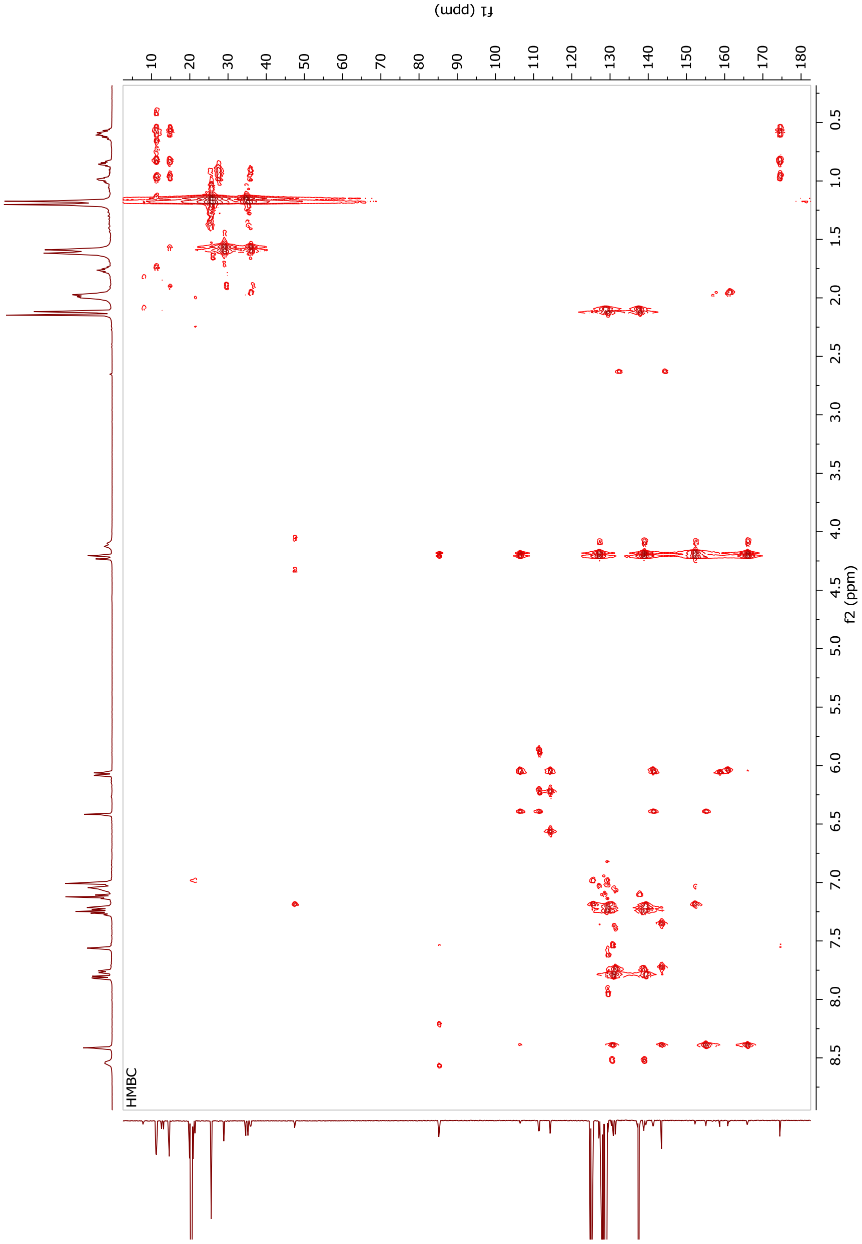

Fig. S24. 2D HMBC (8 Hz optimized) spectrum of oxidative addition complex **C** acquired using the pulse program *hmbcgplpndqf* from the Bruker pulse sequence library. Data were acquired at 500 MHz using 4K × 512 points with 8 transients accumulated per t_1 increment. A delay of 1 s was employed between transients, giving an overall acquisition time of 1 h 35 min. The $^nJ_{CH}$ delay was optimized for 8 Hz. The low-pass *J*-filter was optimized for 160 Hz. Spectral windows in both F2 and F1 were 5,000.0 Hz and 23,883.9 Hz, respectively. Data were processed by linear prediction to 768 in F1. Prior to Fourier transform sine squared apodization and zero-filling to 4096 points in F2 and 1024 points in F1 were applied in both dimensions.

$^1H-^1H$ ROESY (toluene-d_8):

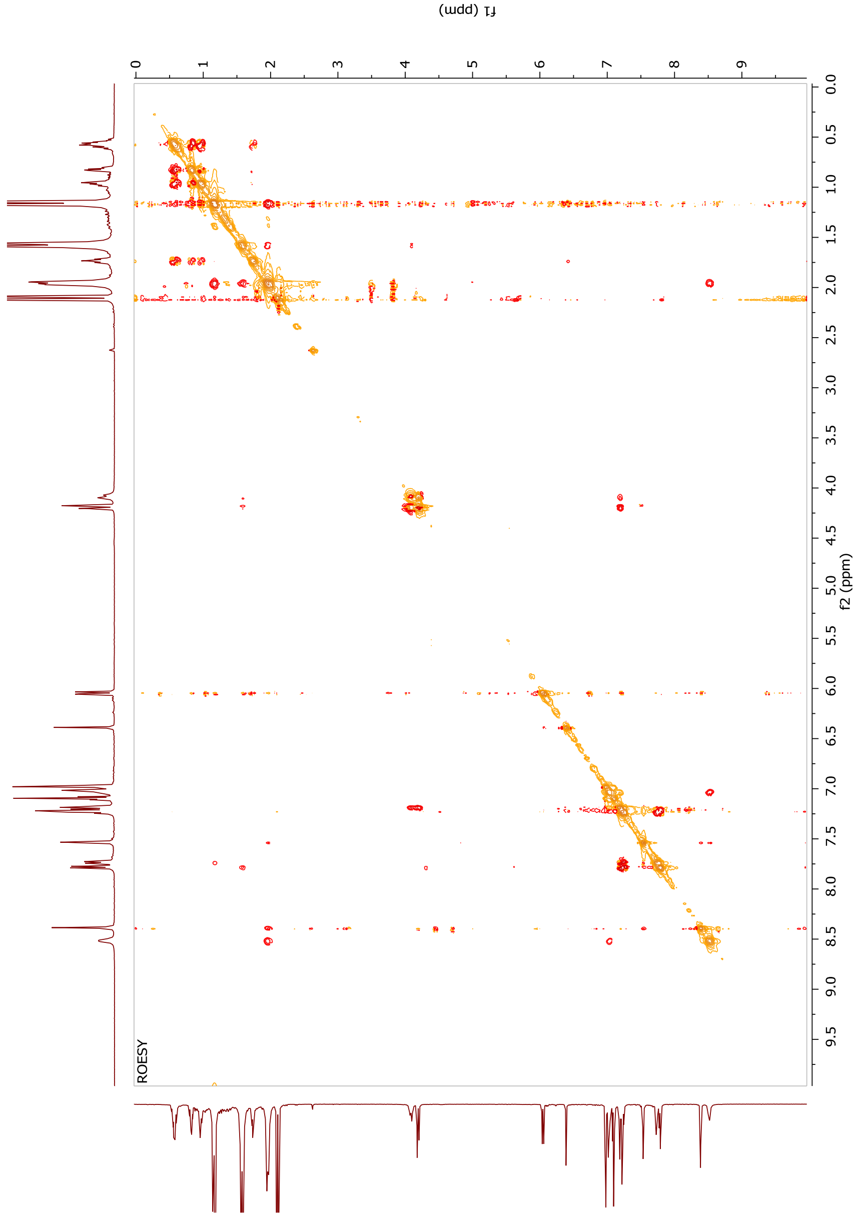

Fig. S25. 2D ROESY spectrum of oxidative addition complex **C** acquired using the pulse program *roesyph.2* from the Bruker pulse sequence library. Data were acquired at 600 MHz using 2048 × 400 points with 16 transients accumulated per t_1 increment. A delay of 2 s was employed between transients, giving an overall acquisition time of 4 h 35 min. The ROESY spinlock mixing time was set to 350 ms. Spectral windows in both F2 and F1 were 5,000.0 Hz. Data were processed by linear prediction to 512 in F1. Prior to Fourier transform sine squared apodization and zero-filling to 4096 points in F2 and 1024 points in F1 were applied in both dimensions.

Key ROESY correlations establishing configuration of oxidative addition complex C

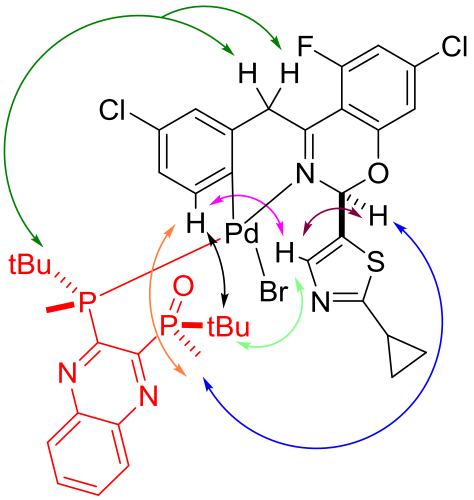

^{1}H-^{1}H ROESY (toluene-d_8):

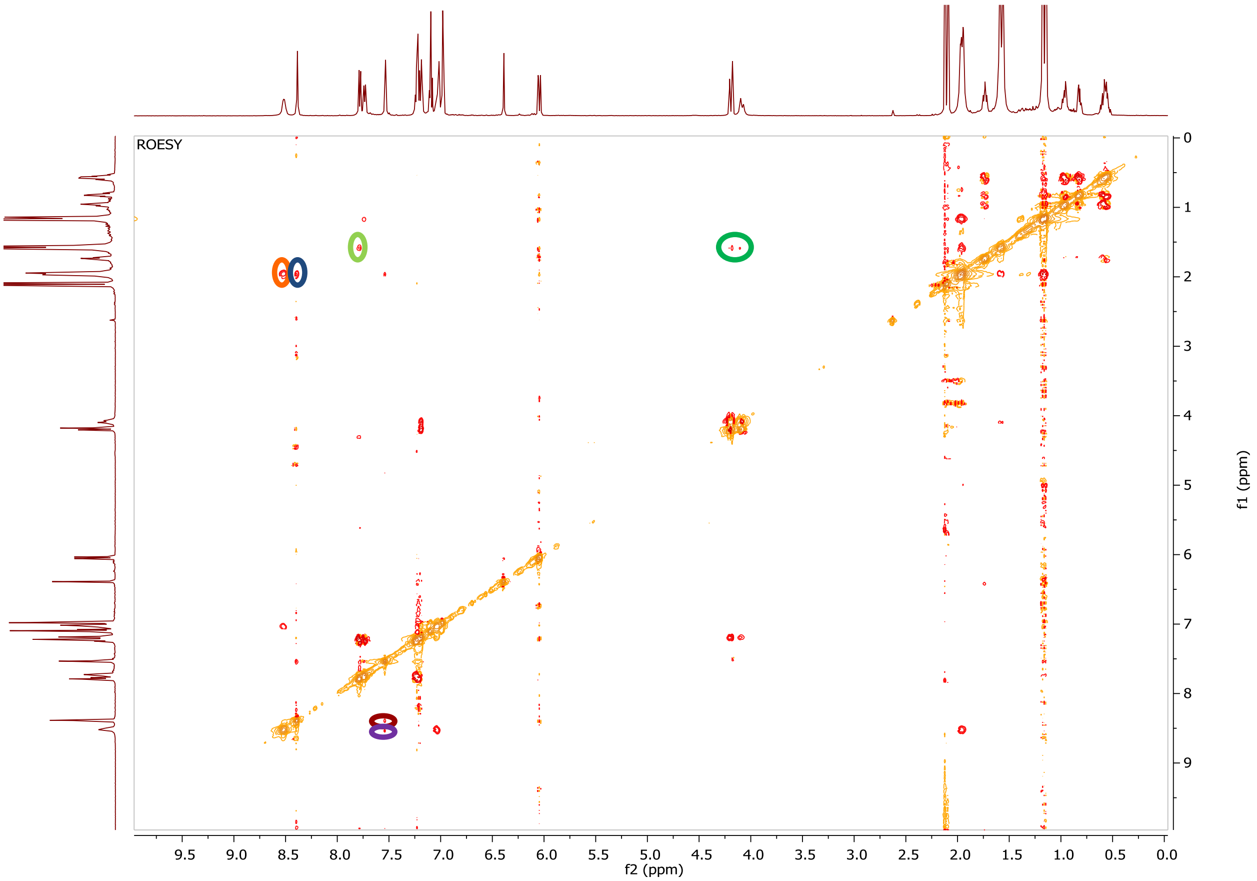

15.2 NMR data for complex **D**

^{1}H NMR (toluene-d_8):

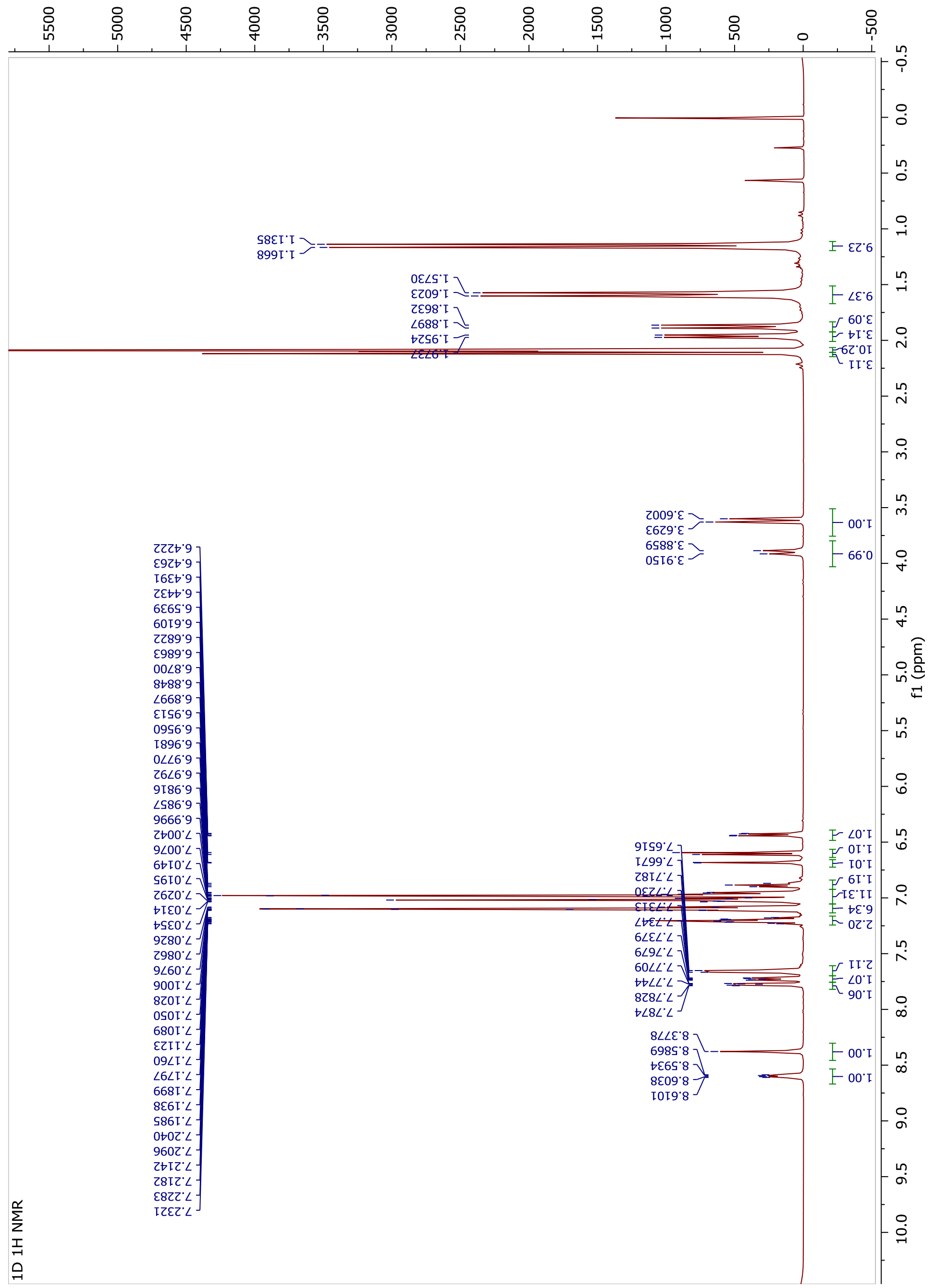

Fig. S26. ^{1}H NMR spectrum of oxidative addition complex **D** acquired at 500 MHz using the pulse program zg30 from the Bruker pulse sequence library. A spectral width of 5498.53 Hz was acquired with 64K data points.

^{13}C NMR (toluene-d_8):

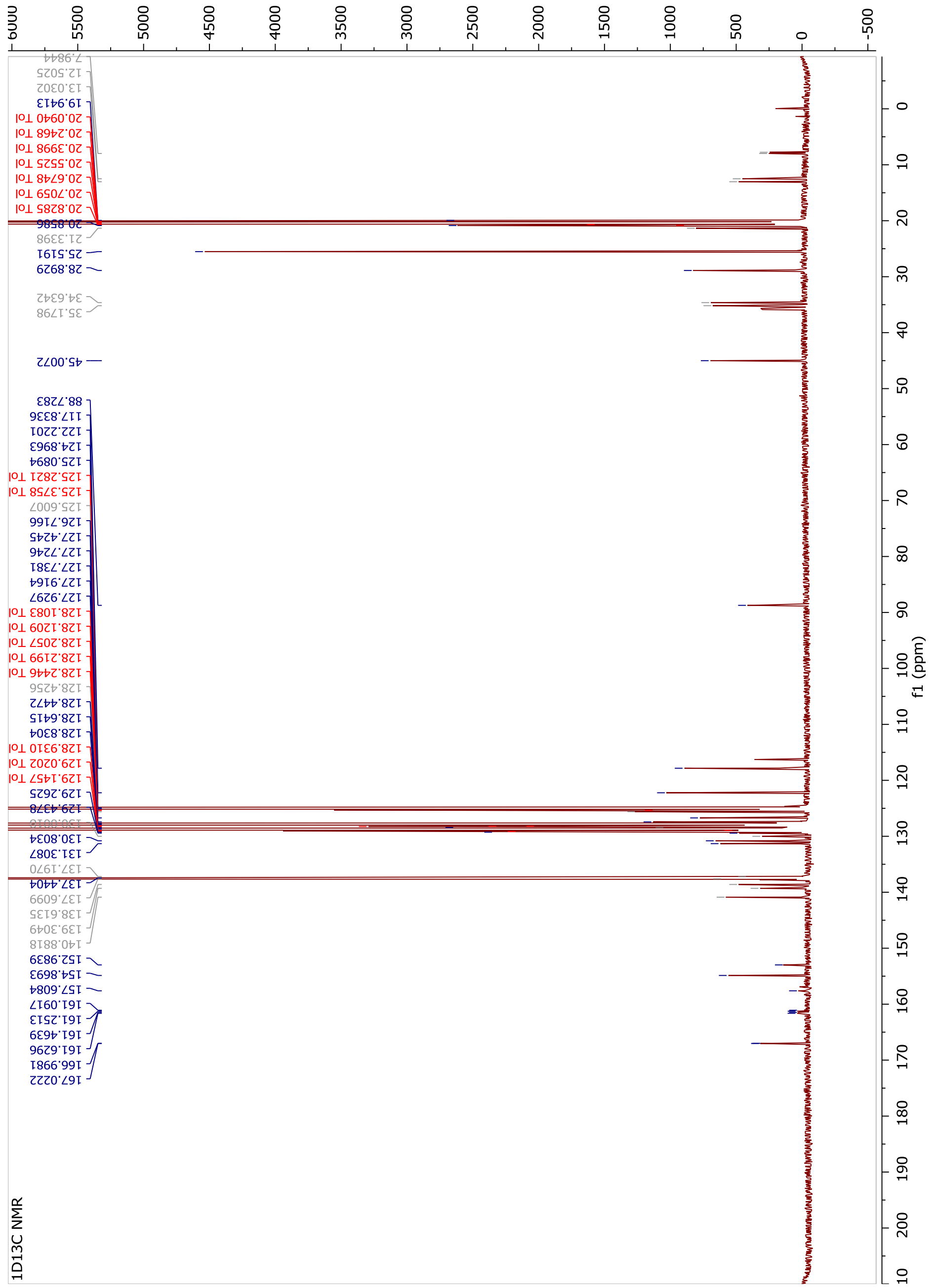

Fig. S27. ^{13}C NMR spectrum of oxidative addition complex **D** acquired at 500 MHz and using the pulse program *zgpg30* from the Bruker pulse sequence library. A spectral width of 27,573.53 Hz was acquired with 128K data points.

^{1}H-^{1}H COSY (toluene-d_8):

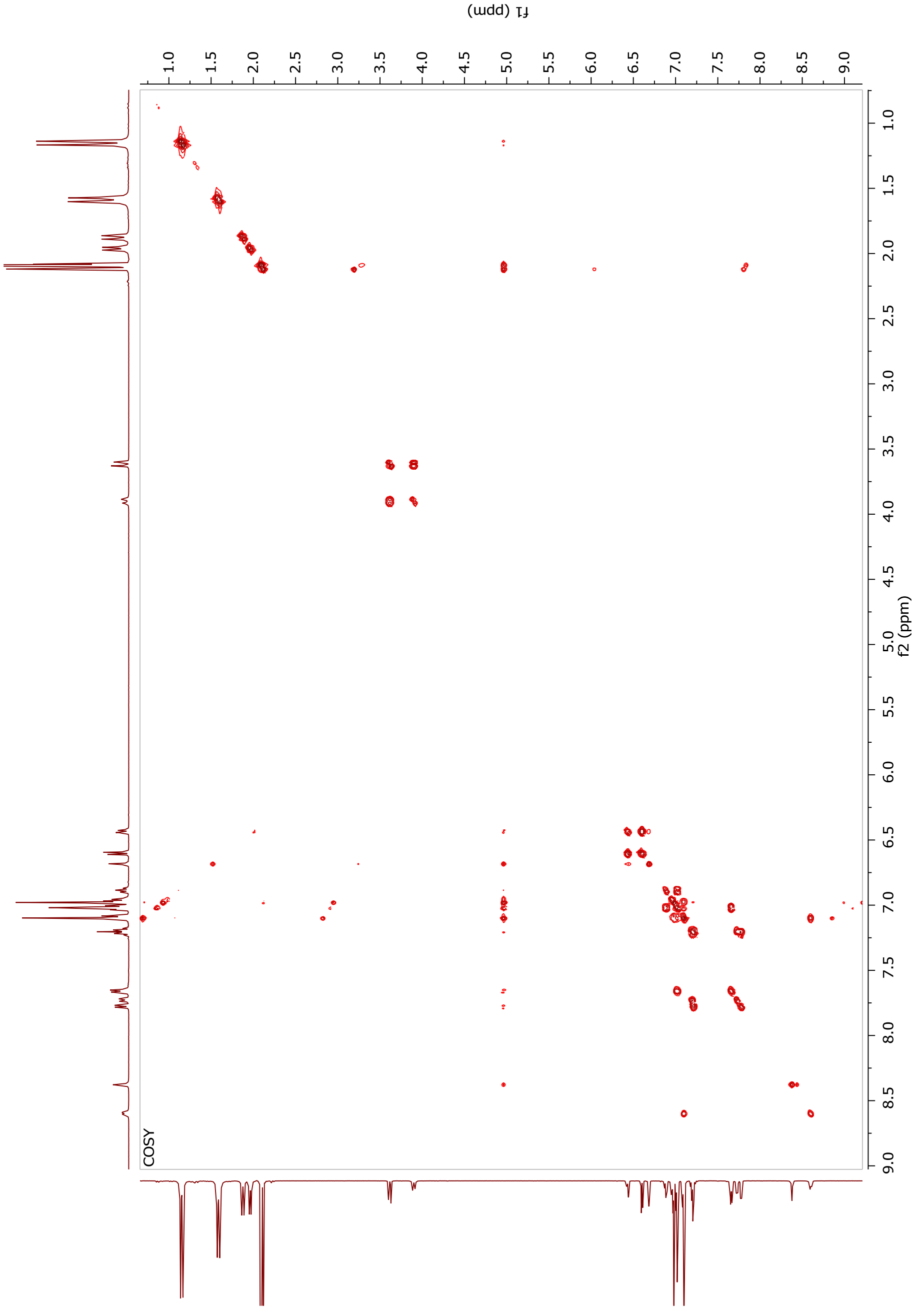

Fig. S28. 2D COSY spectrum of oxidative addition complex **D** acquired using the pulse program *cosygpqf* from the Bruker pulse sequence library. Data were acquired at 600 MHz using 2024 × 256 points with 4 transients accumulated per t_1 increment. A delay of 1 s was employed between transients, giving an overall acquisition time of 38 min 12s. Spectral windows in both F2 and F1 were 5,151.099 Hz. Data were processed by linear prediction to 512 in F1. Prior to Fourier transform sine apodization and zero-filling to 4096 points in F2 and 1024 points in F1 were applied in both dimensions.

^{1}H-^{13}C Pure Shift-Multiplicity Edited-HSQC (toluene-d_8):

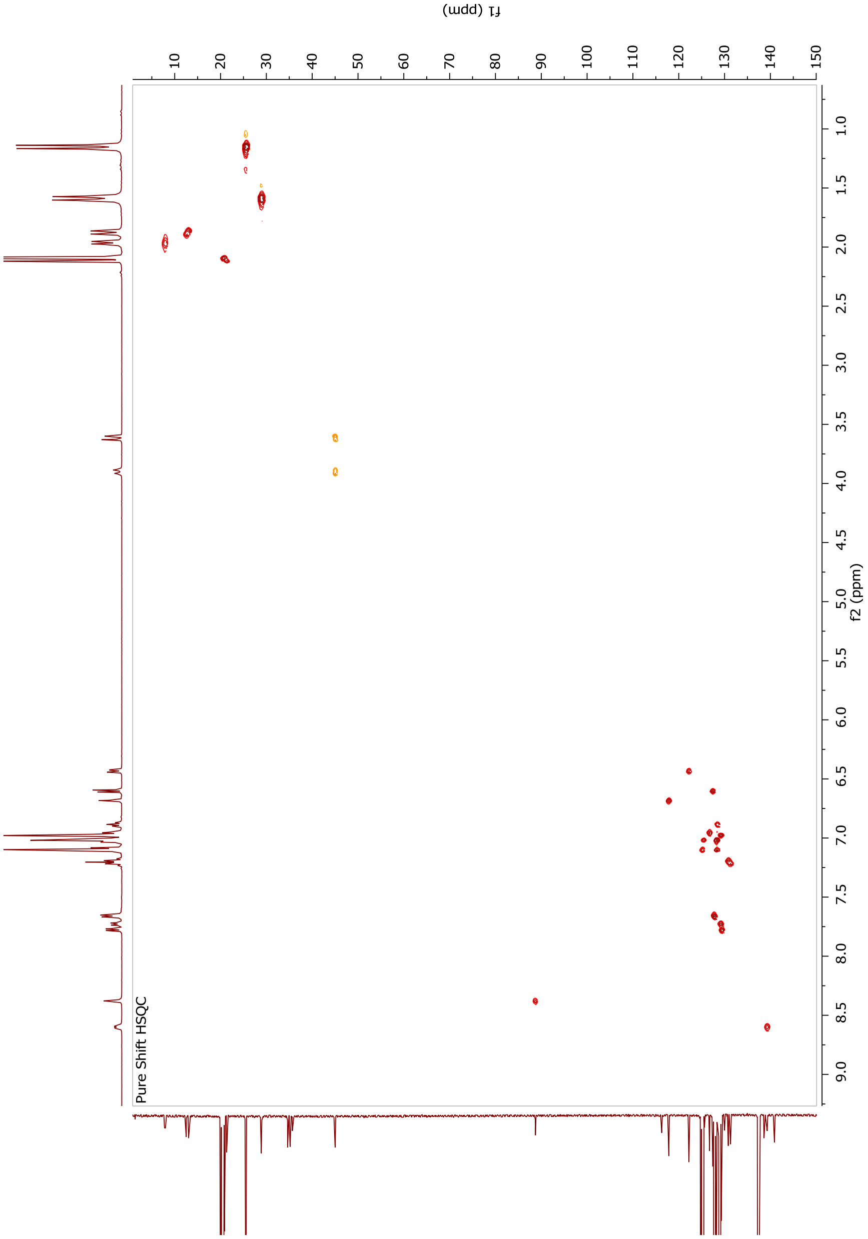

Fig. S29. 2D PS-ME-HSQC spectrum of oxidative addition complex **D**. Methine and methyl resonances are shown in red; methylenes are shown in orange. Data were acquired at 500 MHz using 2048 × 256 points with 4 transients accumulated per t_1 increment. A delay of 1 s was employed between transients, giving an overall acquisition time of 23 min 43 s. The $^{1}J_{CH}$ delay was optimized for 145 Hz. Acquisition t_2 time was 0.204 s and the number of loops for homonuclear, BIRD-based acquisition was set to 8. Spectral windows in both F2 and F1 were 5,000.0 Hz and 21,369.4 Hz, respectively. Prior to Fourier transform sine squared apodization and zero-filling to 4096 points in F2 and 1024 points in F1 were applied in both dimensions.

^{1}H-^{13}C HMBC (toluene-d_8):

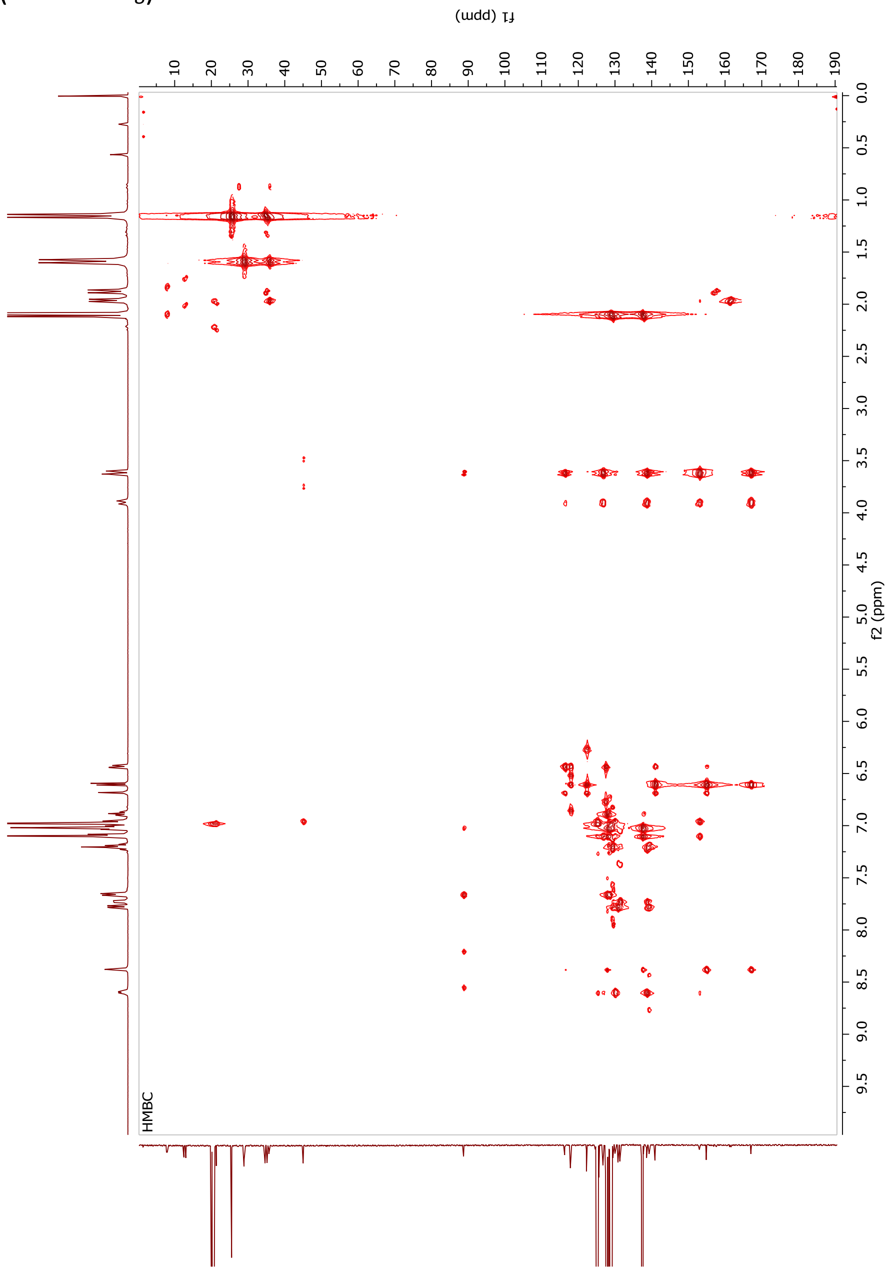

Fig. S30. 2D HMBC (8 Hz optimized) spectrum of oxidative addition complex **D** acquired using the pulse program *hmbcgplpndqf* from the Bruker pulse sequence library. Data were acquired at 500 MHz using 4K × 512 points with 8 transients accumulated per t_1 increment. A delay of 1 s was employed between transients, giving an overall acquisition time of 1h 35m. The $^{n}J_{CH}$ delay was optimized for 8 Hz. The low-pass *J*-filter was optimized for 160 Hz. Spectral windows in both F2 and F1 were 5,000.0 Hz and 23,883.9 Hz, respectively. Data were processed by linear prediction to 768 in F1. Prior to Fourier transform sine squared apodization and zero-filling to 4096 points in F2 and 1024 points in F1 were applied in both dimensions.

^1H-^1H ROESY (toluene-d_8):

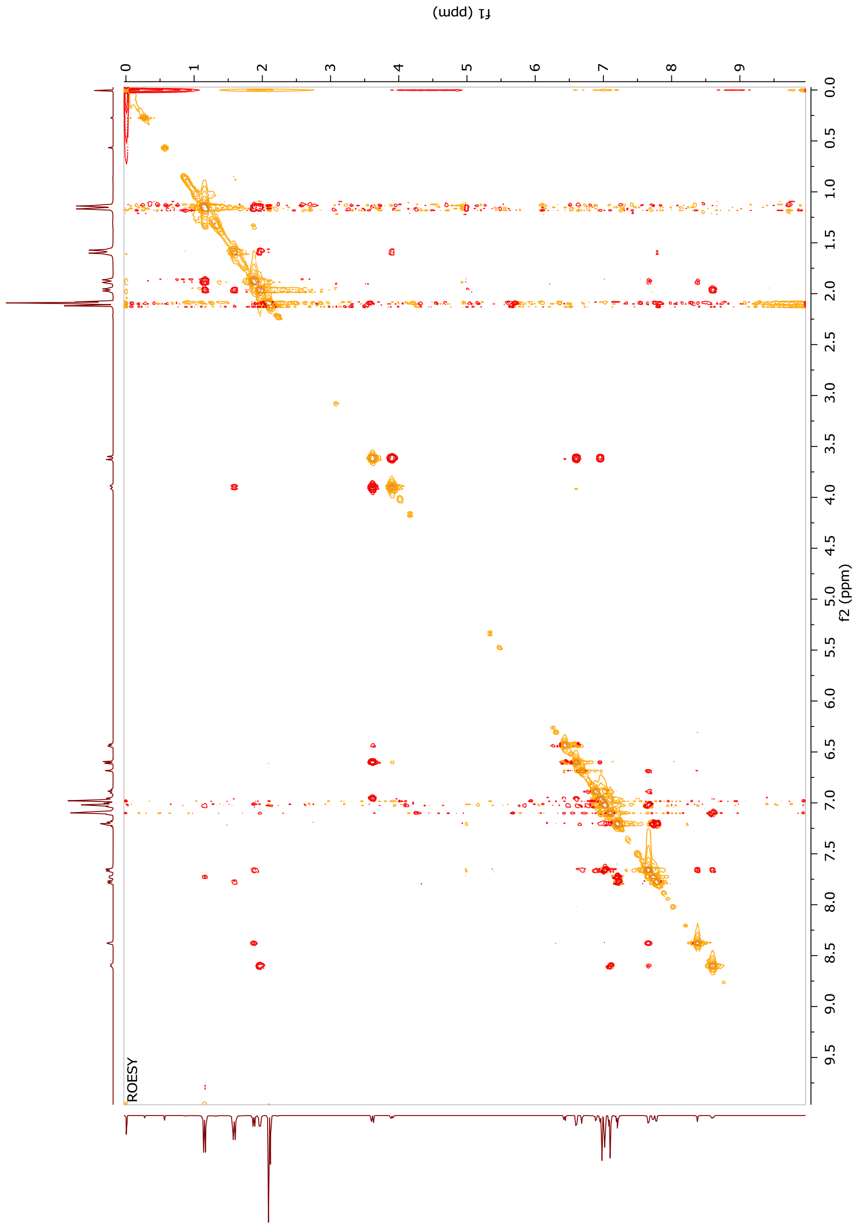

Fig. S31. 2D ROESY spectrum of oxidative addition complex **C** acquired using the pulse program *roesyph.2* from the Bruker pulse sequence library. Data were acquired at 600 MHz using 2048 × 400 points with 16 transients accumulated per t_1 increment. A delay of 2 s was employed between transients, giving an overall acquisition time of 4 h 35 min. The ROESY spinlock mixing time was set to 350 ms. Spectral windows in both F2 and F1 were 5,000.0 Hz. Data were processed by linear prediction to 512 in F1. Prior to Fourier transform sine squared apodization and zero-filling to 4096 points in F2 and 1024 points in F1 were applied in both dimensions.

Key ROESY correlations establishing configuration of oxidative addition complex D

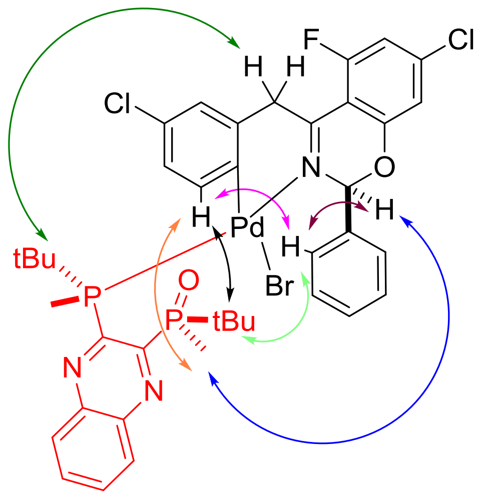

^1H-^1H ROESY (toluene-d_8):

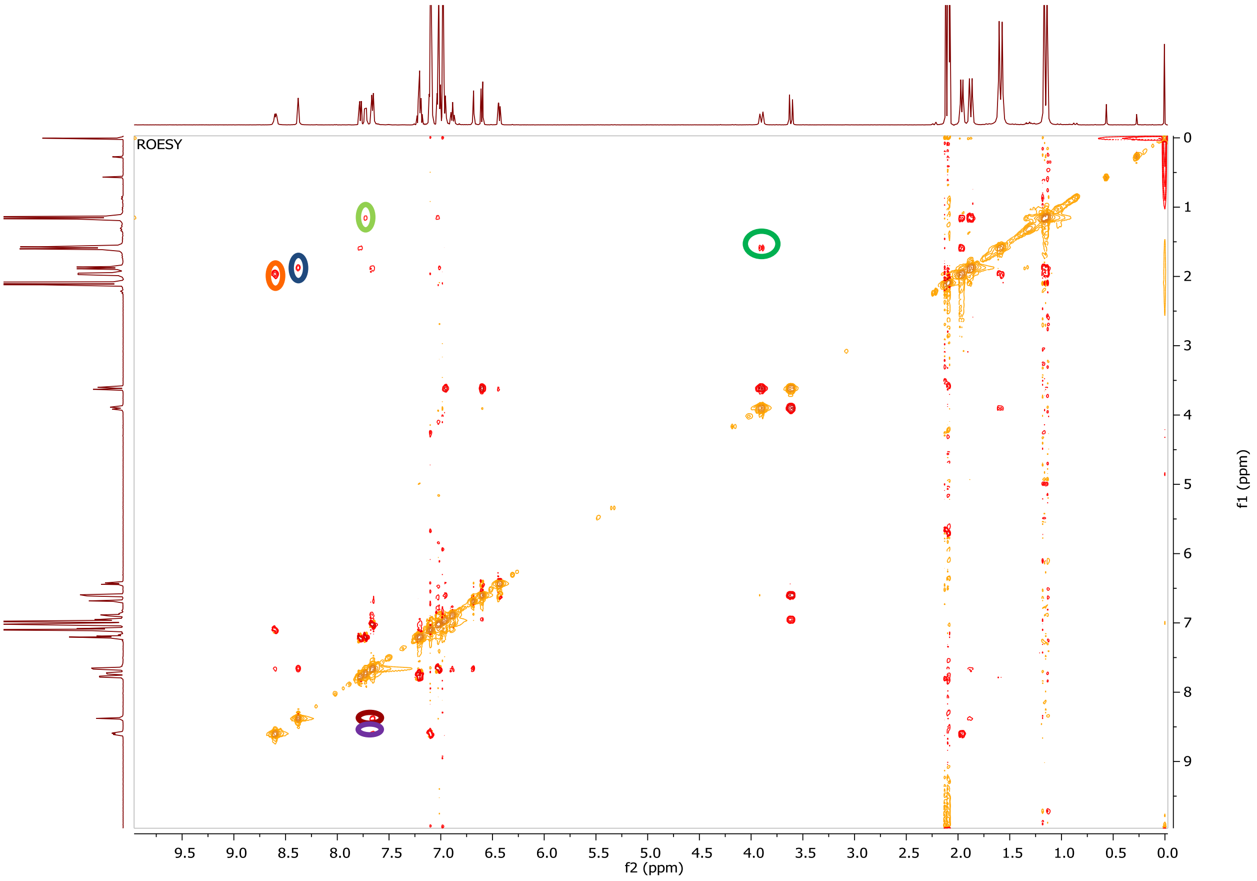

16. DFT calculations

16.1 Structural determination of complex **C** and **D**

Input conformations of **C** and **D** were built and starting conformations were minimized using the D2 corrected B3LYP[2,3,4] or M06-2X[5] density functionals in combination with the 6-31G** basis set[6,7] on all atoms except palladium, which was modeled using the Lanl2DZ basis set[8,9] and Lanl2DZ effective core potential (ECP). Relative electronic energies and free energies were compared to identify preferred conformations and diastereomeric/regioisomeric preferences (while many conformers were investigated; only lowest energy conformers for any complex are provided below). All stationary points were confirmed as minima by running frequency calculations. Density functional calculations were run using Gaussian g09.d01.[10]

Variation in the coordination connectivity of the phosphine ligand and bromine to the central Pd metal was sampled (effectively a 180° rotation of these two groups placing them on opposite sides of Pd in the two possible coordination models (see Fig. S32 **D**-I versus **D**-III). The largest conformational degree of freedom was noted in the ability of the methylene linker of the substrate to repucker driving a large conformational change of the substrate coordination to Pd (see Fig. S32 **D**-I versus **D**-II indicating different ring puckering for *R* vs *S*). A series of conformations for each diastereomer catalyst complex (both epimers at the phenyl carbon) were sampled and the lowest energy complexes were selected for further analysis. Fig. S32 provides the lowest energy conformers of various catalyst complexes. The lowest energy configuration/conformation of the catalyst complex is to the (*S*) enantiomer of the substrate **D**-I (Table S4). The lowest energy conformation of the (*R*) enantiomer of the substrate is ~1-3

[2] Becke, A. D. *J. Chem. Phys.* **1993**, *98*, 5648.
[3] Lee, C. T.; Yang, W. T.; Parr, R. G. *Physical Review* B **1988**, *37*, 785.
[4] Miehlich, B.; Savin, A.; Stoll, H.; Preuss, H. *Chemical Physics Letters* **1989**, *157*, 200.
[5] Zhao, Y.; Truhlar, D. G. *Theoretical Chemistry Accounts* **2008**, *120*, 215.
[6] Hehre, W. J.; Ditchfield, R.; Pople, J. A. *J. Chem. Phys* **1972**, *56*, 2257.
[7] Ditchfield, R.; Hehre, W. J.; Pople, J. A. *J. Chem. Phys*. **1971**, *54*, 724.
[8] Hay, P. J.; Wadt, W. R. *J. Chem. Phys.* **1985**, *82*, 299.
[9] Wadt, W. R.; Hay, P. J. *J. Chem. Phys.* **1985**, 82, 284.
[10] Frisch, M. J.; Trucks, G. W.; Schlegel, H. B.; Scuseria, G. E.; Robb, M. A.; Cheeseman, J. R.; Scalmani, G.; Barone, V.; Mennucci, B.; Petersson, G. A. and others. Gaussian 09, Revision D.01. Wallingford, CT: Gaussian, Inc.; **2009**.

kcal/mol less favorable and is only made possible after concerted inversion/repuckering of the methylene linker since inversion of the phenyl substituent of the lowest energy complex **D**-I is sterically not allowed due to a clash with bromine. Inverting the position of the bromine and phosphine ligand was predicted to give less stable complexes by at least 11-13 kcal/mol. Removal of the phenyl substituent leaving only a dihydro in its place indicates that the global conformation of the complex shown in **D**-I is the lowest energy configuration and conformation (data not shown).

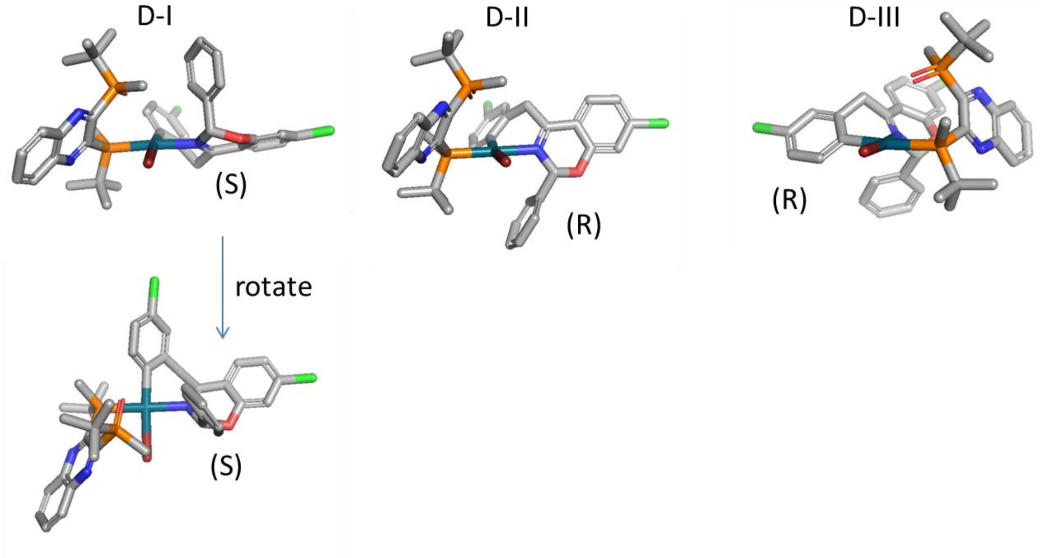

Fig. S32. Minima for three complexes of the M06-2X **D**: **D**-I is the lowest energy orientation of ligands coordinating the Pd with the methylene linker of the (*S*) substrate pointed 'down' below the coordination plane; **D**-II is the lowest energy complex of the substrate enantiomer (*R*) bound to catalyst; **D**-III is the lowest energy conformation of the altered coordination of the bromine and phosphine ligands. Hydrogens are omitted for clarity, coloring of C,N,O,P,Cl,Br and Pd: grey, blue, red, orange, green, brown, and teal, respectively.

After determination of the favorable conformation and configuration of **D**-I, the phenyl group was replaced with a 2-cyclopropylthiazole (**C**). Again, the relative energy of the (*R*) configuration of the substrate was more favorable compared to the complex binding the (*S*) configuration for all substitution patterns (Fig. S33). The configuration of the 2-cyclopropylthiazole corresponding to **C**-III was not pursued. The crystal structure of **D** was determined (MDK052) and is depicted in Fig. S34. Upon minimization the observed crystal conformation of the phenyl ring is calculated

to be ~0.5 kcal/mol higher in energy than the lowest energy conformation seen in conformer type A. This slight variation in computationally modeled rotamer and crystallographically observed rotamer is attributed to stacking interactions in the crystal lattice (Fig. S35).

Table S4: Relative energies for catalyst complexes

	B3LYP-D2[1] ΔE	B3LYP-D2[1] ΔG[3]	M06-2X[1] ΔE	M06-2X[1] ΔG[3]
Phenyl Derivative				
**D-I**	0.0	0.0	0.0	0.0
**D-II**	3.6	2.6	3.3	1.6
**D-III**	11.1	11.3	12.4	11.0
**MDK052**[4]	0.6	0.8	not min[5]	not min
**MDK052-rotamer**[6]	0.0	0.0	0.0	0.0
Thiazole Derivative				
**C-I**	0.0[2]	0.0	0.0	0.0
**C-II**	3.4	3.2	3.3	3.9
**C-III**	N/A	N/A	N/A	N/A

[1] Basis set = 6-31G** on all atoms accept Pd = Lanl2DZ + ECP
[2] Relative energy in kcals/mol
[3] Frequencies were not scaled
[4] crystal structure minimized
[5] phenyl rotamer observed crystallographically not a minima on the M06-2X surface
[6] crystal structure minimized with alternate phenyl rotamer which equates to conformer **D-I**

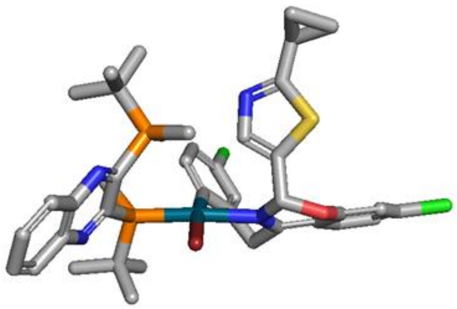

Fig. S33. Minima for the catalyst complex **C-I** of the (*R*) enantiomer of the 2-cyclopropylthiazole substrate.

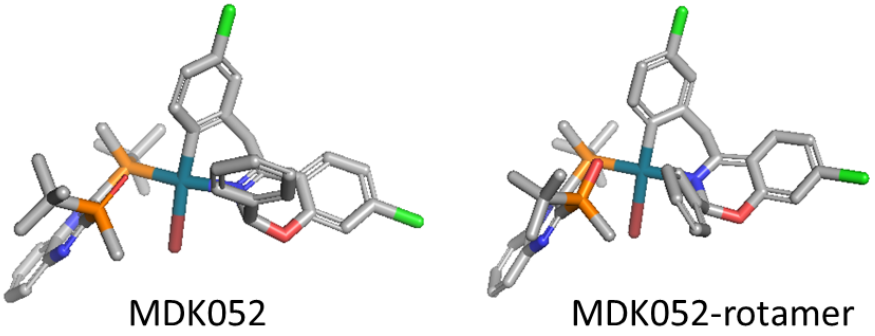

Fig. S34. Crystal structure of **C** on the left, and the minimized structure with the phenyl ring rotated at right. The complex is of the (*R*) enantiomer of the phenyl substrate.

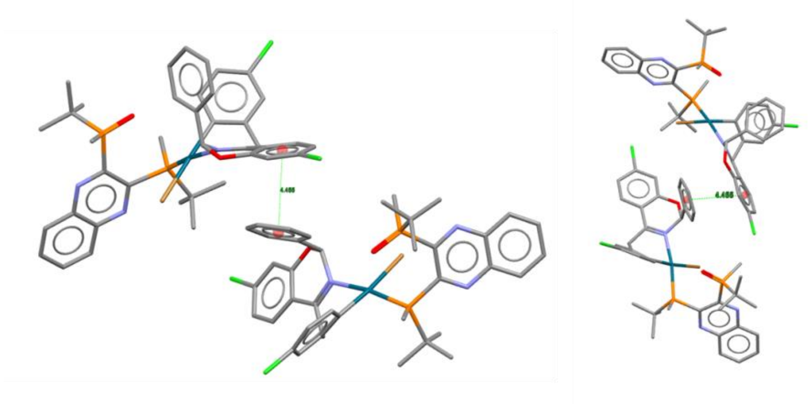

Fig. S35. Crystal structure of MDK052 showing close contacts in the crystal lattice. A stacking interaction amongst adjacent complexes shown as a green dotted line (4.6 Å apart) influences the phenyl ring to rotate away from the minimum energy conformation.

16.2 Calculations on oxidative addition transition states and deprotonation pathways

Choice of the optimal density functional and basis set for modeling this reaction pathway was made by comparing results from geometry optimization of catalytic complex **D** with the X-ray crystal structure. Minimal variation was observed for the functionals and basis sets tested, with M06/6-31G*:SDD[11],[12] demonstrating good correlation to the bond distances and dihedrals observed experimentally (Table S5). Coordinates of minima are provided in the SI zip file.

Table S5: Relative bond distances between geometry optimized **D** and crystal structure. Lower cumulative relative distance indicates better correspondence between the two poses.

			P–Pd	P(=0)–Pd	Br–Pd	C–Pd	N–Pd	Cumulative
**cam-B3LYP-D2**	6-31G**	LANL2DZ	0.05	-0.45	0.14	0.03	0.05	0.72
**cam-B3LYP-D3**	6-31G**	LANL2DZ	0.04	-0.49	0.10	0.02	0.03	0.69
**B3LYP-D3**	6-31G**	LANL2DZ	0.05	-0.46	0.14	0.04	0.05	0.74
	6-31G**	SDD	0.04	-0.46	0.10	0.03	0.04	0.68
**B3LYP-D2**	6-31G**	LANL2DZ	0.02	-0.51	0.13	0.04	0.06	0.77
	6-31G**	SDD	0.02	-0.51	0.10	0.04	0.04	0.71
	6-31+G**	LANL2DZ	0.04	-0.53	0.14	0.04	0.05	0.80
**M06**	6-31G**	SDD	0.04	-0.46	0.09	0.04	0.04	0.67
	6-31G**	LANL2DZ	0.05	-0.47	0.11	0.04	0.06	0.74
	6-31G*	SDD	0.05	-0.46	0.08	0.04	0.04	0.67
	6-31G*	LANL2DZ	0.05	-0.47	0.12	0.04	0.06	0.75
	6-31+G*	SDD	0.05	-0.56	0.10	0.04	0.05	0.80
	6-311+G**	SDD	0.05	-0.46	0.08	0.04	0.04	0.67
**M06L**	6-31G*	SDD	0.03	-0.53	0.12	0.03	0.07	0.78
	6-31+G*	SDD	0.05	-0.54	0.13	0.03	0.05	0.80
**M062X**	6-31G*	LANL2DZ	0.06	-0.53	0.15	0.01	0.09	0.84
	6-31G**	LANL2DZ	0.06	-0.53	0.15	0.01	0.09	0.84
	6-31G**	SDD	0.04	-0.54	0.13	0.00	0.08	0.80
	6-311+G**	SDD	0.05	-0.54	0.12	0.01	0.08	0.80

[11] Fuenteabla, P.; Preuss, H.; Stoll, H.; v. Szentpály, L. *Chem. Phys. Lett.*, **1982**, *89*, 418.
[12] Andrae, D.; Haeussermann, U.; Dolg, M.; Stoll, H.; Preuss, H. *Theor. Chem. Acc.*, **1990,** *77,* 123.

Transition state calculations were undertaken with M06/6-31G*:SDD from these low-energy reactant, product, and intermediate states by freezing the bond-forming or bond-breaking of interest and allowing the rest of the complex to optimize. The presence of a single imaginary frequency identified a transition state, which was further confirmed through intrinsic reaction coordinate scans in the reactant and product directions. Influence of solvent was captured through calculation of single point energies in toluene with the Minnesota model[13] and dispersion corrections, using the M06 functional[4] and 6-31+G*:SDD basis sets. Coordinates for all transition states and intermediates discussed are provided in the SI zip file.

Transition states for oxidative addition

Computational studies of the formation of complex **C** were carried out using M06/6-31+G(d) with SDD for Pd and Br in Gaussian 09. Solvation in toluene was accounted for with the SMD solvent model. The $\Delta\Delta G^{\ddagger}$ (ca. 1.5 kcal/mol) favors the formation of the desired complex **C** (*S* configuration at the hemiaminal position) over the undesired diastereomer (*R*-**C**). Complex **C** is also favored thermodynamically over the undesired diastereomer *R*-**C** by 3.67 kcal/mol. The *R*-**C** substrate must undergo re-puckering at the benzylic methylene in order to alleviate steric clashes during formation of the oxidative addition complex. As a result, the TS-*R* (Fig. S36) is a later transition state than TS-*S*, and can be described by longer distances and wider angles between the reacting atoms. The Pd-C bond being formed is 2.14 Å in the TS-*R* and 2.05 Å in TS-*S*, and the Pd-Br bond being formed is 3.58 Å in TS-*R* and only 3.07 Å in TS-*S*. Also, the Br-C-Pd angle is sharper in the TS-*S* than the TS-*R*, with angles of 90.9° and 109.2° respectively.

[13] Marenich, A. V.; Cramer, C. J.; Truhlar, D. G. *J. Phys. Chem. B.* **2009**, *113*, 6378.

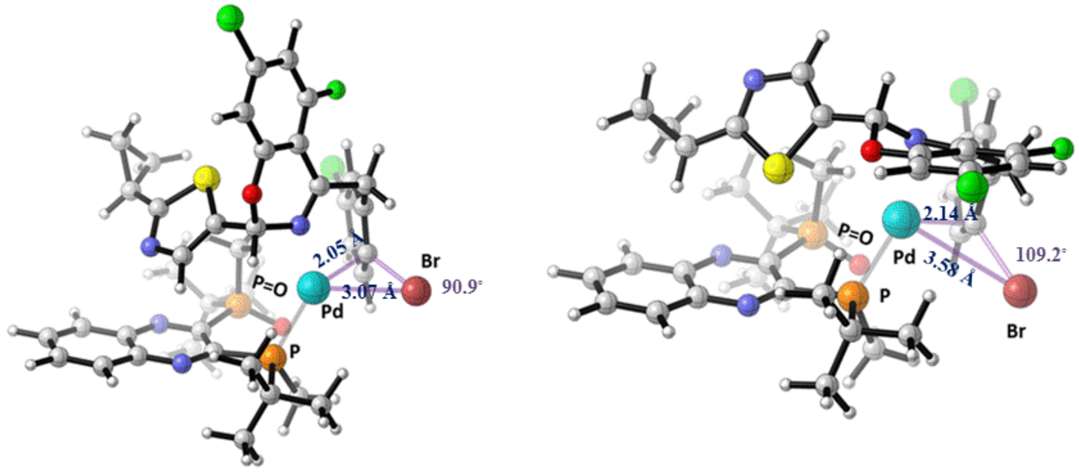

TS-*S* TS-*R*

Fig. S36. Transition states for the oxidative addition step in formation of **C** (TS-*S*) and *R*-**C** (TS-*R*).

<u>Deprotonation Intermediates</u>

Computational calculations focused on determining whether interconversion between *R*-**C** and **C** occurred through a zwitterionic open form **J** or anionic form **G**. The zwitterion **J** undergoes a massive conformational rearrangement to stabilize the charge, resulting in a high barrier to ring closure. However, a ring-open intermediate **G** is stabilized by the reaction solvent and competent to undergo closure to form **H**. These conformational differences between the energetically-accessible ring-open intermediate **G** relative to the higher-energy zwitterion **J** are displayed in Fig. S37.

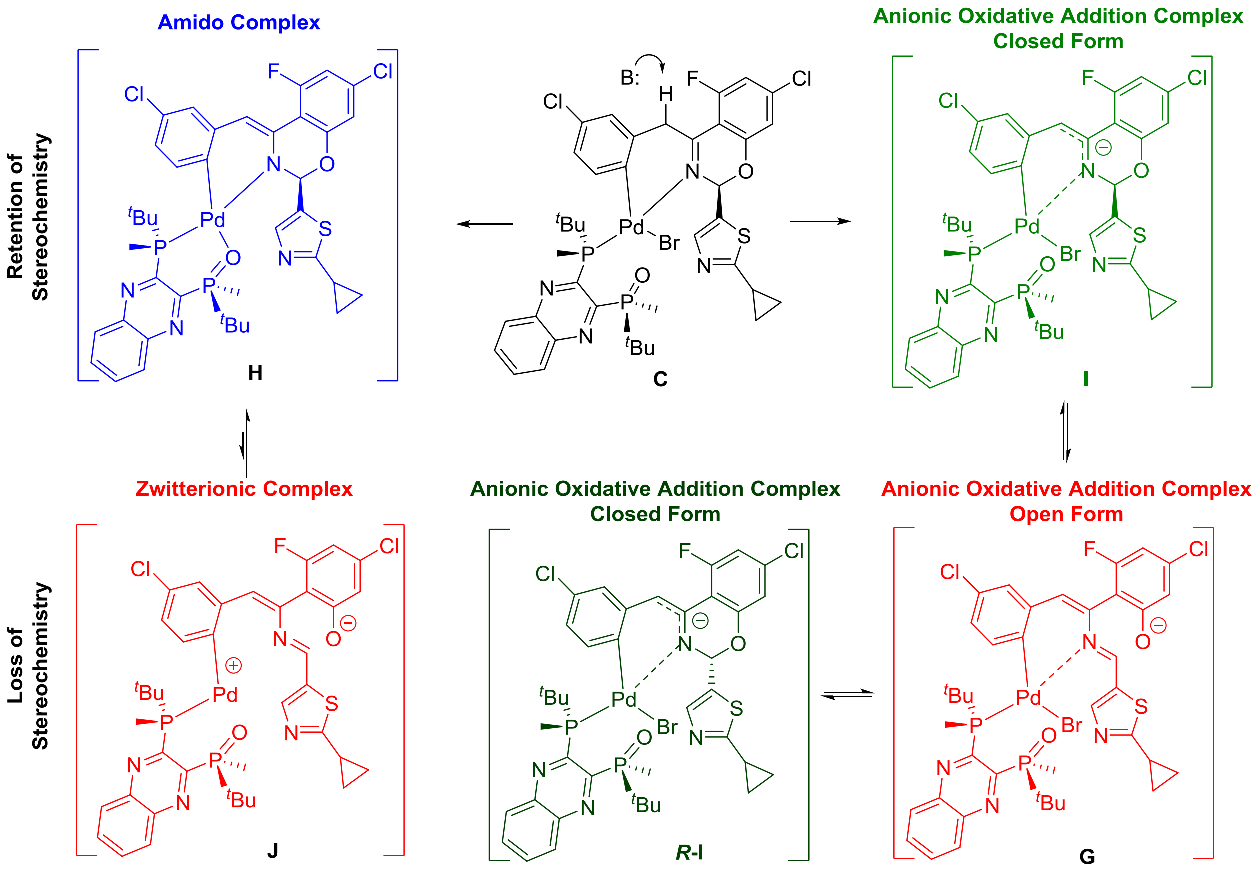

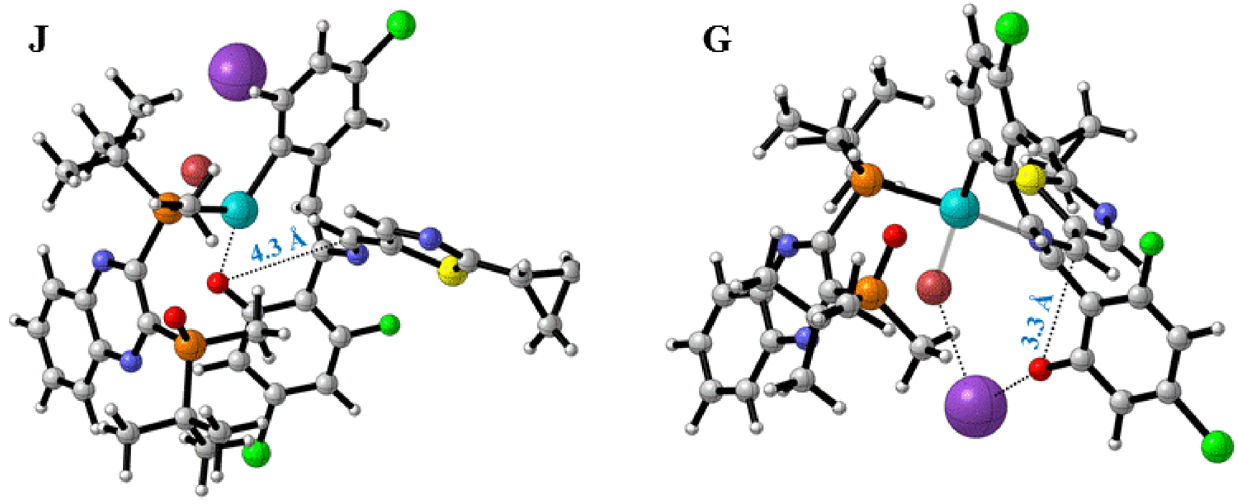

Fig. S37. Low energy conformation in toluene of the ring-open deprotonated intermediate **G** compared with the zwitterion species **J**. The massive structural rearrangement adopted to stabilize the lowest-energy zwitterionic ring-open species results in a conformation that is much less thermodynamically preferred than the closed conformation **H**. The deprotonated intermediate **G** is stabilized in the presence of KBr and is thermodynamically accessible from **I**.
